# A novel approach for T7 bacteriophage genome integration of exogenous DNA

**DOI:** 10.1186/s13036-019-0224-x

**Published:** 2020-01-16

**Authors:** Ying Liu, Hongxing Huang, Hua Wang, Yan Zhang

**Affiliations:** 1 State Key Laboratory of Biocontrol, MOE Key Laboratory of Gene Function and Regulation, School of Life Sciences, Sun Yat-sen University, Guangzhou, 510006 Guangdong People’s Republic of China; 2 Department of Oral and Maxillofacial Surgery, Guanghua School of Stomatology, Hospital of Stomatology and Guangdong, Provincial Key Laboratory of Stomatology, SunYat-sen University, Guangzhou, 510055 People’s Republic of China

**Keywords:** T7 bacteriophage, ΦC31 integrase, Integration, Gene knock-in

## Abstract

**Background:**

The comparatively small genome, well elucidated functional genomics and rapid life cycle confer T7 bacteriophage with great advantages for bio-application. Genetic manipulation of T7 genome plays a key role in T7 related applications. As one of the important aspects in T7 phage genetic modification, gene knock-in refers to two main approaches including direct genetic manipulation in vitro and recombineering. Neither of these available methods are efficient enough to support the development of innovative applications capitalizing on T7 bio-system and thus there is room for novel strategies that address this issue. Integration mediated by the ΦC31 integrase is one of the most robust site-specific recombination systems. ΦC31 integrases with enhanced activity and specificity have been developed such that it is ideal to effectuate exogenous DNA knock-in of T7 phage with advanced ΦC31 integrase.

**Methods:**

Plasmid construction was conducted by routine molecular cloning technology. The engineered T7 bacteriophages were constructed through homologous recombination with corresponding plasmids and the functional T7 phage was designated as T7∆G10G11-attB. In the integration reaction, hosts with both executive plasmids (pEXM4) and donor plasmids (pMCBK) were lysed by T7∆G10G11-attB. Progenies of T7 phages that integrated with pMCBK were isolated in restrict hosts and validated by sequencing. T7∆G10G11-attB capacity limit was explored by another integration reactions with donor plasmids that contain exogenous DNA of various lengths.

**Results:**

T7∆G10G11-attB exhibits abortive growth in restrictive hosts, and a bacterial attachment site recognized by ΦC31 integrase (attB) was confirmed to be present in the T7∆G10G11-attB genome via sequencing. The integration reaction demonstrated that plasmids containing the corresponding phage attachment site (attP) could be integrated into the T7∆G10G11-attB genome. The candidate recombinant phage was isolated and validated to have integrated exogenous DNA. The maximum capacity of T7∆G10G11-attB was explored, and it’s found that insertion of exogenous DNA sequences longer than 2 kbp long can be accommodated stably.

**Conclusion:**

We advanced and established a novel approach for gene knock-in into the T7 genome using ΦC31 integrase.

## Background

T7 bacteriophage was first systematically investigated by Studier et al. [1], who initiated the seminal work for the following research and bio-application development. T7 phage embraces excellent biological properties, including the rapid growth cycle as a lytic bacteriophage and the tough capsid that encapsulates a genome of approximately 40 kbp [2]. In addition to these advantages, the well elucidated functional genomics makes T7 phage one of the most popular biosystems for a wide range of applications. For instance, the T7 system serves as an alternative robust peptide display platform for targeted drug screening with high specificity and affinity [3]. Moreover, a T7-based vector has been developed for the delivery of DNA vaccines into mammalian cells [4]. However, limit in T7-related applications exists owning to the inconvenience genetically modifying its genome. The T7 biosystem deserves further development as a promising tool in widespread fields.

No applications with T7 phage can be carried out without genetic modification. To our knowledge, mutagenesis of lytic bacteriophages like T7, mostly relies on recombineering, a technology in which an exogenous fragment flanked by homologous regions replace the wild-type allele followed by appropriate screening steps [5]. Higher recombination efficiency has been achieved by exploiting phage-derived proteins that mediate recombination events, e.g., the lambda Red system [6]. The conventional method used for recombinant screening is the double-layer bacterial lawn technique, which allows researchers to distinguish defective recombinants from thousands of phages on a restrictive bacterial lawn. The low screening efficiency of double-layer bacterial lawn technique has spurred researchers to seek substitute approaches. Until now, recombinant screening via selectable markers occurs to be a smarter method, expediting the genetic modification of bacteriophage. However, available markers for T7 phage are rare and generally the helper hosts are also required. For example, the engineered *Escherichia coli* strain BW25113ΔtrxA was employed to screen T7 recombinants containing *trxA* by Kiro et al. [7]. Thioredoxin (TrxA), as a subunit of T7 DNA polymerase, is the obligatory host factor for T7 growth [8]. In principle, recombinants that produce TrxA by itself can form the plagues on BW25113ΔtrxA lawns. Therefore, *trxA* represents a useful select marker for T7 mutant screening, which remarkably accelerates the process of identifying recombinants.

In addition to recombineering, direct genetic manipulation of the T7 genome via either electroporation [9] or in vitro packaging technique [10] has been attempted. Initially, the 40 kbp T7 genome is subjected to a series of genetic manipulations via molecular cloning technology. Subsequently, the modified T7 DNA is recovered by either direct transformation into host competent cells or by in vitro packaging into viable virions via commercial packaging kits. However, both genetic manipulation and electroporation of T7 genome are sophisticated technologies that unavailable for normal laboratories. These inconveniences have significantly hampered the widespread use of T7 phage.

Practically, the application of T7 biosystem involves knock-in of gene of interest (GOI) along with a compatible host system [11–13]. For example, distinct binders can be panned from T7 display libraries in which DNA fragments encoding the randomized peptides have been inserted into the T7 genome [14]. Hence, an efficient method to effectuate exogenous DNA knock-in of T7 genome plays a crucial role in T7 bio-application.

Here, we aim to simplify gene knock-in of T7 system, and we introduce a novel strategy for addressing this issue. Site-specific integration has been widely used in a variety of species, including bacterium, fly, zebrafish and mammals [15–19]. Among the numerous integration systems, ΦC31 integrase has emerged as an outstanding one in many fields; thus, we were inspired to develop an in vivo T7 integration system mediated by ΦC31 integrase. Furthermore, researchers have developed engineered ΦC31 integrases with higher activity and specificity [20, 21]. These advanced ΦC31 integrases contribute to establish the T7 in vivo integration system with high activity.

In our design, exogenous DNA is integrated into the T7 genome at a specific site via plasmids that serve as the donor vector. Functional ΦC31 integrases can be produced by another plasmid in the host. To achieve these outcomes, the bacterial attachment site (attB) recognized by ΦC31 integrases must be present within the engineered T7 genome and the corresponding bacteriophage attachment site (attP) must be cloned into the donor plasmid. As T7 phages grow in its hosts, an attP-containing donor plasmid would be integrated into an attB-containing T7 genome through site-specific recombination mediated by ΦC31 integrase [22]. To isolate the recombinants that contain the desired donor plasmid, the gene encoding tail tubular protein of T7 phage (*gene 11*) is deleted from the engineered T7. In consequence, the T7 *gene 11* present in the donor plasmid as the positive select marker enables the recombinants to survive in restrictive hosts that provide no gp11 product. Via this integration system, knock-in of any desired exogenous DNA with acceptable length (the genome packaging limit) [23] into the T7 genome can be quickly achieved due to the high efficiency of ΦC31 integrases. As far as we are concerned, there is no available approach for T7 genome insertion with comparable simplicity and convenience. We expect that development of innovative T7 phage-based applications will benefit from this in vivo integration system in the future.

## Methods and materials

In this work, we constructed pRFG9, pCDG9 for T7∆G9 recombineering. Next, we constructed pRFG10G11, pCDG10G11 for T7∆G10G11-attB recombineering in the base of the yielded T7∆G9. To build up the integration system, we constructed pEXM3, pEXM4 and pMCBK. In practice, pEXM4 was exploited to the following experiments. To explore the capacity of this integration system, we constructed a series of pMCBK derivatives including pMCBK-CE1, pMCBK-CE2, pMCBK-CE3 and pMCBK-CE4. These plasmids contain inserts of diverse length from 800 bp to 3.0 kbp. Sequence information of constructed plasmids and engineered T7 phages as well as the primer information was documented in Additional file 1. Important results referring to development of this integration system and details of plasmids construction were documented in Additional file 2. The sequencing results of the mentioned plasmids and engineered T7 phages were documented in Additional file 3.

### Plasmid construction

Linearized DNA fragments including the inserts and original vectors were prepared through polymerase chain reaction (PCR) amplification or digestion with restriction enzyme(s). DNA polymerase KOD -plus- (TOYOBO, Japan) was used to amplify the expected products according to manufacturer’s instructions. Briefly, 10× buffer, dNTP mix, template, a pair of primers and KOD –plus- polymerase were mixed and brought to a final volume of 50-μl with deionized water. The PCR protocol was as follows: the pre-denaturing at 95 °C for 2 min, followed by 35 cycles including denaturation at 95 °C for 15 s, annealing at 55 °C for 20 s and elongation at 68 °C for a suitable time. The samples were then maintained at 16 °C. For the restriction enzyme(s) digestion, FastDigest enzymes (Thermo scientific, the USA) were used to yield linearized vectors according to the manufacturer’s instructions. Briefly, 10× FastDigest buffer, restriction enzyme(s) and plasmids were mixed, and the mixture was incubated at 37 °C for 2 h.

Electrophoresis of samples containing 1× loading buffer was conducted in 1% agarose gels made with 1× TAE buffer and containing 0.25 μg/ml ethidium bromide (Thermo Scientific, USA) at 120 V and 150 mA for 30 min. The PCR products and restriction enzyme digestion products were purified with the AxyPrep™ DNA Gel Extraction Kit (Axygen, the USA) according to manufacturer’s instructions. By using of the ClonExpress II One Step Cloning Kit (Vazyme Biotech, China), 0.015-pmol vectors and 0.03-pmol inserts were recombined following the manufacturer’s instructions. Briefly, linearized vectors, inserts, 5× CE II buffer and Exnase II enzyme were mixed in a final volume of 10-μl with deionized water, and the mixture was incubated at 37 °C for 30 min followed by cooling down to 4 °C for 5 min.

Cloning of DNA fragments was conducted with the M5 HiPer pTOPO-Blunt Cloning Kit (Mei5 Biotechnology, China) according to manufacturer’s instructions. Briefly, appropriate amounts of purified PCR-generated DNA fragments were mixed with 1-μl of M5 HiPer pTOPO-Blunt vector and 10× Enhancer, and the 10-μl reaction mixture was incubated at RT for 10 min.

The recombination product or cloning product were directly used to transform DH5α (K-12, F^−^ λ^−^ F80d *lacZ∆M15* Δ (*lacZYA*-*argF*) U169 deoR *recA1 endA1 hsdR17* (rK^−^ mK^+^) *pho*A *supE44 thi-1 gyrA96 relA1*) competent cells as described by Inore et al. [24]. Briefly, the 10-μl of the recombination product or cloning product was added to 100-μl of DH5α competent cells, and the mixture was incubated on ice for 30 min, followed by incubation at 42 °C for 30 s and cooling on ice cold for 2 min. The transformation mixtures were immediately supplemented with 900-μl of 37 °C LB medium, and the mixture was then incubated at 37 °C with shaking at 220 rpm for 1 h to resuscitate the cells. The resuscitated cells were then centrifuged at RT at 2500 g for 1 min, and 900-μl of the supernatant was discarded. The remaining culture was resuspended and spread on LB plates with the corresponding antibiotics. The spread plates were incubated overnight at 37 °C. The next day, candidate clones were verified by colony PCR and confirmed via sequencing. Colony PCR was carried out with a PCR kit (DongSheng biotech, China) according to the manufacturer’s instructions. Briefly, 2x PCR reaction mix, a pair of primers and 1-μl of culture were mixed and brought to a final volume of 10-μl with deionized water. The PCR procedure was as follows: pre-denaturing at 95 °C for 4 min, 30 cycles including denaturing at 95 °C for 15 s, annealing at 55 °C for 15 s and elongation at 72 °C for a suitable time. The samples were then maintained at 16 °C.

Plasmids were purified with the HiPure Plasmid Micro Kit (Magen, China) according to manufacturer’s instructions. Briefly, verified clones were inoculated in 3-ml of LB medium with the corresponding antibiotic and cultured over night at 37 °C with shaking at 220 rpm. The plasmids were extracted from the full 3-ml culture the next day.

### Lysis of host bacterium and plaque formation assay

*Escherichia coli* strain BL21(B, F^−^ ompT hsdSB (rB^−^ mB^−^) *gal dcm*) was used for T7 phage infection and the plaque formation assay. BL21 competent cells were transformed with functional plasmids to yield the desired hosts. In general, log-phase bacterial hosts were infected with T7 phage at a multiplicity of infection (MOI) of between 0.1 to 1, while the MOI was increased to 10 for the integration reactions. The infected bacterial hosts were cultured at 37 °C with shaking at 220 rpm for approximately 3 h until the cultures became transparent. The absorbance at 600 nm (OD600) of the cultures was measured to monitor the progress of the host lysis.

The plaque formation assays were carried out by the double-layer bacterial lawn method. Briefly, T7 phage were diluted to a suitable concentration, and 100-μl of the diluted T7 phage was mixed with 200-μl of host culture and 3 ml of 46 °C, 0.7% agarose LB medium. The mixture was then poured onto plates coated with 1.2% agarose LB medium. The plates were then incubated at room temperature over-night.

### Construction of engineered T7 phage

**Fig. 1:**
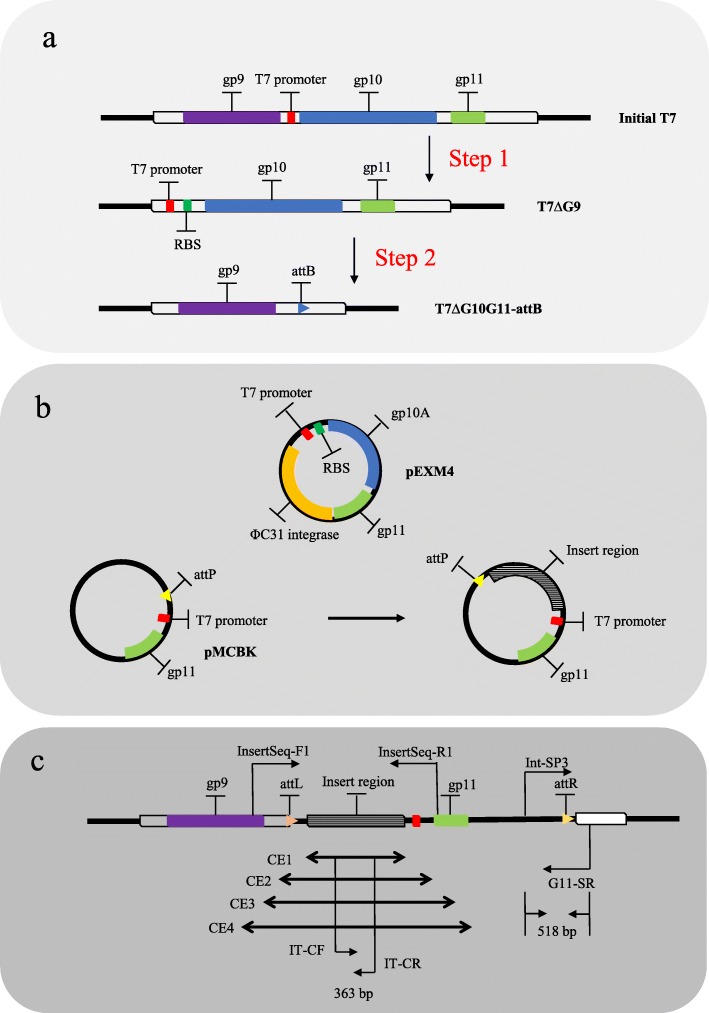
Overview of T7 phage in vivo integration system. **a**. T7∆G10G11-attB construction scheme and genetic structure. **b**. Plasmid profiles of executive plasmid and donor plasmid as well as the donor plasmid with exogenous DNA. Executive plasmid is designated as pEXM4. Donor plasmid is designated as pMCBK. **c**. Expected T7∆G10G11-attB genetic structure with exogenous DNA integration. CE represents insertion of capacity exploration. RBS represents Ribosome Binding Sequence

Homologous recombineering was carried out to construct the T7ΔG9 and T7ΔG10G11-attB engineered T7 phages (Fig. 1a). Initially, plasmids for recombination (pRFG9, pRFG10G11) and plasmids for maintaining the engineered T7 phage (pCDG9, pCDG10G11) were prepared as described above. The T7Select10-3b phage from the T7Select®10–3 Cloning Kit (Merch, Germany) was used to infect BL21-pRFG9 cells, and diluted lysates with approximately 10^4 phage formation unit (pfu) phages were displayed on BL21-C9 lawns. Candidate recombinants were verified by PCR and sequencing. Positive T7ΔG9 recombinants were purified by 3 rounds of isolation on BL21-C9 lawns. T7ΔG10G11-attB was derived from T7ΔG9 by analogous recombineering. Briefly, T7ΔG9 was used to infect BL21-pRFG10G11 cells, and diluted lysates with approximately 10^4 pfu phages were displayed on BL21-C10C11 lawns. Candidate recombinants were verified via PCR and sequencing (see Additional file 3). Positive T7ΔG10G11-attB recombinants were purified by 5 rounds of isolation.

### Integration reactions

*E.coli* strain MC1061F^−^ (*araD139 Δ (araA-leu)7697 Δ (lac)X74 gal*K16 *gal*E15*(GalS) lambda- e14- mcrA0 relA1 rpsL150* (Str^R^) *spoT1 mcrB1 hsdR2*) was used for the integration reactions, as its transformation efficiency is higher than that of BL21. MC1061F^−^ competent cells (Weidi Biotechnology, China) were co-transformed with pEXM4 (EPs, rendering them ampicillin resistant) and pMCBK (DPs, rendering them kanamycin resistant). The resuscitated cells were spread on LB culture plates containing ampicillin (100 μg/ml) and kanamycin (50 μg/ml). The plates were cultured at 37 °C over-night. The next day, candidate clones were verified via colony PCR. A volume of 300-μl of confirmed co-transformants growing in log phase were centrifuged at RT at 2500 g for 1 min to pellet the bacteria. Subsequently, the pellets were resuspended in 300-μl of LB medium containing only ampicillin (100 μg/ml), and T7ΔG10G11-attB was added at an MOI of 10. The infected transformants were cultured at 37 °C with shaking at 220 rpm for approximately 3 h until the cultures became transparent. Transformants containing only EP were subjected to the same reaction with T7ΔG10G11-attB (MOI = 10) as a negative control. After the cultures were completely lysed, they were centrifuged at 10000×g at RT for 5 min, and the supernatant was harvested to another tube. Recombinants were detected via PCR with 0.1x-diluted supernatant as the template using the PCR kit (DongSheng Biotech, China) as described above.

Recombinants were further isolated by plaque formation assays. Serial dilutions of the integration reaction products (i.e., the lysate supernatants) were produced, and the 10^-4x, 10^-5× or 10^-6× dilutions (100 μl each) were applied to lawns of BL21-C10. Dozens of plaques were randomly selected and verified by PCR and sequencing of specific regions in the recombinants. As shown in Fig. 1c, the universal primers Int-SP3 and G11-SR used for recombinant detection lied in DP and T7ΔG10G11-attB respectively, such that expected PCR products could only be produced from recombinants. Fragments containing the left attachment site (attL, the product of the integration reaction mediated by ΦC31 integrase) and the right attachment site (attR, the product of the integration reaction mediated by ΦC31 integrase) were amplified by PCR and sequenced to validate the integration of the exogenous DNA (see Additional file 3).

### Capacity limit exploration

Exogenous DNA fragments of 865 bp, 1296 bp, 2399 bp and 3058 bp were derived from the ORF of *SpCas9*(pEM-Cas9HF1-recA56 [25], which was purchased from Addgene (The nonprofit plasmid repository, http://www.addgene.org/). These inserts would not be translated into SpCas9 components due to frameshifts, despite having the same nucleotide sequences. DPs containing these 4 inserts (pMCBK-CE1, pMCBK-CE2, pMCBK-CE3, and pMCBK-CE4) were constructed, and integration reactions were carried out to obtain recombinants that harbor the expected inserts as described above. Two pairs of primers were used to identify the recombinants via PCR, i.e., the Int-SP3 and G11-SR primer pair and the specific primer pair IT-CF and IT-CR. The latter primer pair (IT-CF and IT-CR), was designed based on the common regions in the four inserts (as shown in Fig. 1c) such that expected PCR products could only be amplified from positive recombinants. In another identical integration assay exclusive of pMCBK-CE1 and pMCBK-CE2, an ensemble pair of primers including InsertSeq-F1 and InsertSeq-R1 was used to corroborate the integration event (Fig. 1c). This pair of primers covers the insert region as well as the flanking sequences in T7 genome. The candidate recombinants isolated from integration reactions were sequenced to validate the success of the integration and the presence of exogenous *SpCas9* DNA fragments (see Additional file 3).

### Data processing

**Fig. 2:**
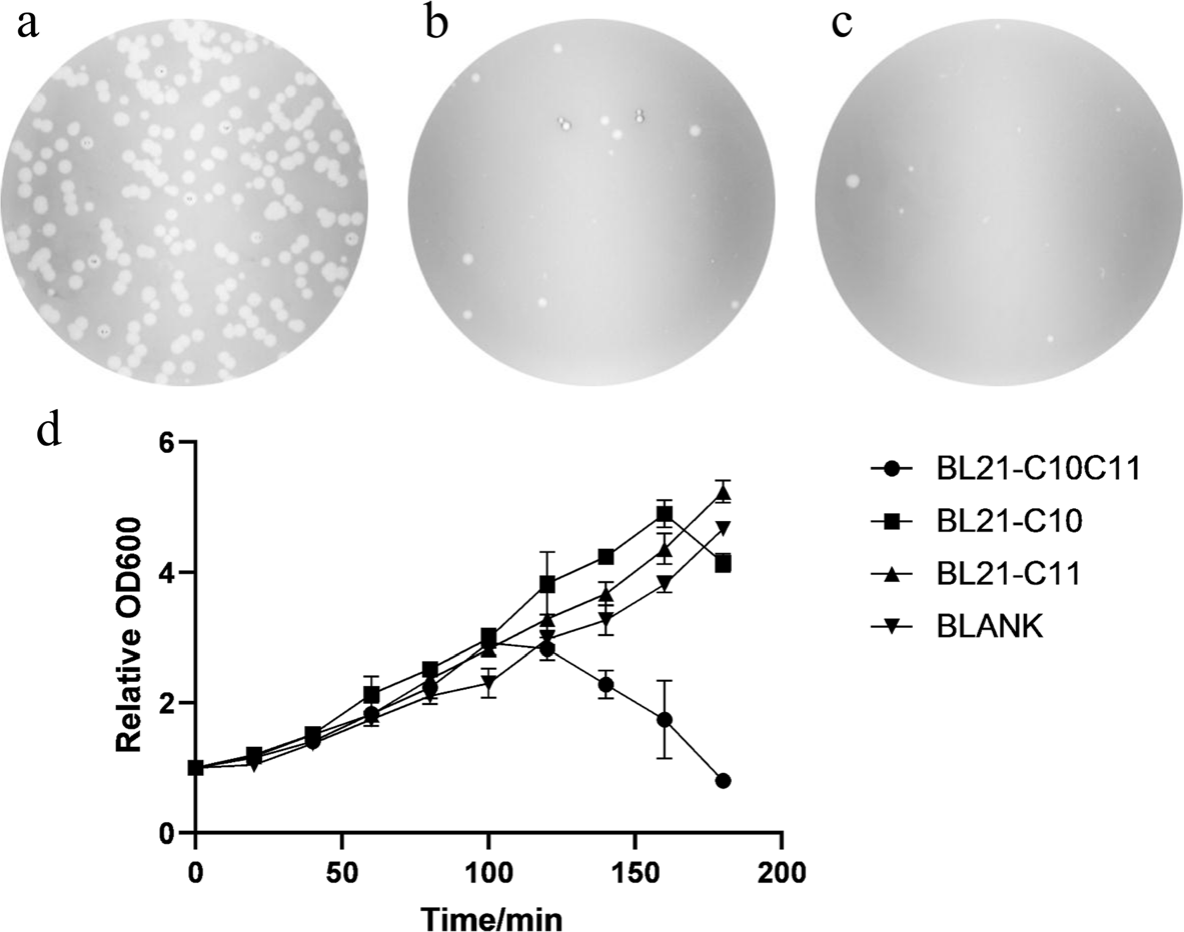
T7∆G10G11-attB plague formation assay and culture lysis assay. **a**. approximately 10^3 pfu T7∆G10G11-attB seeding to BL21-C10C11 lawn. **b**. approximately 10^4 pfu T7∆G10G11-attB seeding to BL21-C10 lawn. **c**. approximately 10^4 pfu T7∆G10G11-attB seeding to BL21-C11 lawn. **d**. Culture lysis curves. Blank represents BL21-C10C11 infected with no T7∆G10G11-attB

The absorbances at 600 nm of culture lysates in hosts lysis assay were put in GraphPad Prism Version 8.0.1 for creating the Fig. 2d graph.

## Results

### Abortive growth of engineered T7 phage in restrictive hosts

The engineered T7 phage T7ΔG10G11-attB was constructed via recombineering technology. Both *gene 10* (encoding the T7 capsid protein) and *gene 11* (encoding the T7 tail tubular protein) are deleted from T7ΔG10G11-attB, and T7ΔG10G11-attB contains an inserted attB site (the bacterial attachment site recognized by ΦC31 integrase) downstream *gene 9* (encoding the T7 scaffold protein) within its genome (Fig. 1a). The genotype of T7ΔG10G11-attB was validated by sequencing of the specific region see Additional file 3. Plaque formation and culture lysis assays were conducted to demonstrate that normal growth of T7ΔG10G11-attB depends on host-provided gp10A and gp11 proteins. Few plaques formed on BL21-C10 lawns (BL21 strain transformed with pCDG10) or BL21-C11 lawns (BL21 strain transformed with pCDG11) even when approximately 10^4 pfu phages were seeded onto the lawns (Fig. 2a to c). However, great amount of plaques formed on the BL21-C10C11 lawns (BL21 strain transformed with pCDG10G11) seeded with a total of 10^3 pfu phages. Furthermore, the lysis curves of the cultures showed that the OD600 values of the BL21-C10C11 culture started to decrease after 120 min and decreased below the initial level within 3 h, while the OD600 values of the other control groups continued to increase over 150 min (Fig. 2d).

### Successful integration of donor plasmids into the T7ΔG10G11-attB genome

**Fig. 3:**
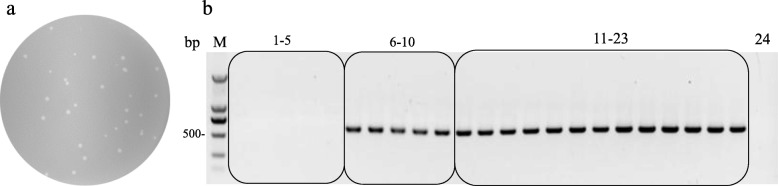
Integration assay. **a**. Plague formation assay with diluted lysate from integration product. **b**. Recombinants detect PCR with universal primer pair Int-SP3 and G11-SR. Expected product is 518 bp. Panel 1–5 indicate negative control group lysates in quintuplicates that contain no pMCBK but pEXM4. Panel 6–10 indicate experimental group lysates in quintuplicates that contain both pEXM4 and pMCBK. Panel 11–23 indicate 13 isolated candidate recombinants from plague formation assay. Panel 24 indicates Blank contral with deionized water as PCR template

A donor plasmid containing the attP site (pMCBK) was used in the integration reaction. Expected recombinants containing pMCBK could survive in BL21-C10 cells due to the pMCBK-supplied gp11. Diluted reaction lysates containing approximately 10^3 pfu mixed phages were subjected to plaque formation assays on BL21-C10 lawns. Dozens of plaques formed (Fig. 3a), and 13 candidate recombinants were randomly selected for polymerase chain reaction (PCR) analysis using the Int-SP3 and G11-SR universal primer pair. As shown in Fig. 3b, the 518-bp expected PCR product was detected from all the candidate recombinants and in the reaction lysates after the integration reaction. These observations showed that recombinants generated via this integration system can be easily isolated with high efficiency. Two of the 13 candidate recombinants were sequenced to validate pMCBK integration (see Additional file 3).

### Exogenous DNA longer than 2 kbp can be stably integrated into the T7ΔG10G11-attB genome

**Fig. 4:**
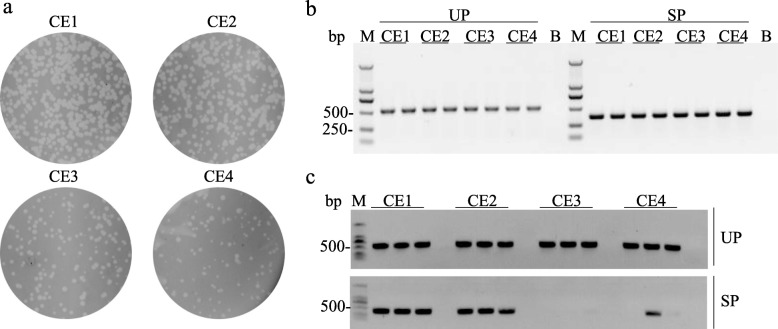
Capacity exploration. **a**. Detect PCR of recombinants from reaction lysates. **b**. Plague formation assay in 4 groups with equal quantities of phages. **c**. Recombinants detect PCR of isolates from 4 groups. UP represents the universal primer pair Int-SP3 and G11-SR used in the assay with an expected product of 518-bp. SP represents the specific primer pair IT-CF and IT-CR with an expected product of 363-bp. CE1–4 represents four inserts in duplicates respectively

**Fig. 5:**
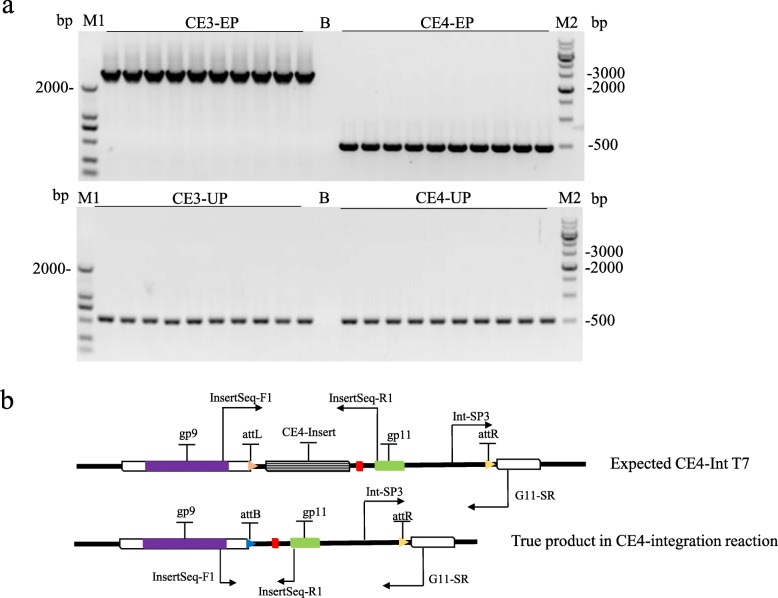
Validation of longer insert integration. **a**. Detect PCR of candidate CE3-integrated and CE4-integrated recombinants. EP represents the ensemble primer pair InsertSeq-F1 and InsertSeq-R1. The correspongding products in CE3-integrated and CE4-Integrated recombinants were 2.89 kbp and 3.549 kbp respectively. UP represents the universal primer pair Int-SP3 and G11-SR with an expected product of 518 bp. B represents Blank contral with deionized water as PCR template. **b**. Gene structure of CE4-integration reaction product was analyzed after sequencing. Red solid rectangle represents T7 promoter

To explore the capacity of this integration system, four donor plasmids containing various lengths of exogenous DNA (pMCBK-CE1, pMCBK-CE2, pMCBK-CE3 and pMCBK-CE4) were constructed and subjected to integration reactions. As expected, recombinants were detected in the four reaction lysates, and these recombinants were shown to carry exogenous DNA (Fig. 4b). Next, four groups of candidate recombinants were isolated from plaque formation assays on BL21-C10 lawns. Plaques from all four groups formed, while candidate recombinants from the pMCBK-CE3 and pMCBK-CE4 groups, which corresponding to 2.399 kbp and 3.058 kbp of exogenous DNA respectively, were significantly less abundant than those from the other two groups (Fig. 4a). These results demonstrated that integration with over-long exogenous DNA may result in decreased maturation of infectious virions. The isolated candidate recombinants from the four groups were verified by PCR. The expected band corresponding to the 518-bp product generated via the universal primer pair was present in all four groups; however, the distinct band corresponding to the 363-bp product with the specific primer pair was absent from the pMCBK-CE3 group and from two of the three plaques in the pMCBK-CE4 group (Fig. 4c). To further ascertain the integration of longer inserts, another integration assays with pMCBK-CE3 and pMCBK-CE4 were performed. As shown in Fig. 5a, the distinct band corresponding to a 2890-bp product in CE3-EP group demonstrated that entire insert from pMCBK-CE3 was present in recombinants. pMCBK-CE3 integrated T7 candidate recombinants were validated by sequencing (see Additional file 3). While expected band corresponding to a 518-bp product was observed in both CE3-UP group and CE4-UP group, the band corresponding to a 3549-bp product was absent from CE4-EP group but an approximately 500-bp product was observed (Fig. 5a). This distinct product was further sequenced and aligned with the expected CE4-Integrated T7 recombinants. It’s showed that the entire CE4-insert was lost as expectation, to our surprise, the predicted attL was replaced of attB (Fig. 5b). We assume that retrieve of attB site resulted from the homologous recombination events during T7 genome replication. These outcomes indicated that integration reaction will not be affected by the length of exogenous DNA, nevertheless, the inserts longer than 3 kbp were lost as the T7 phage proliferated.

## Discussion

Another popular phage system, M13, performs well not only for display technology [26] but also for bioengineering, such as enzyme evolution and soluble expression evolution in prokaryotic cells [27]. The versatile phagemid technology, which builds the linkage between DNA information and their protein products through phage packaging of an episomal circle DNA, is one of the most remarkable features of the M13 system. The M13 packaging signal and the accessary helper phage serve as the indispensable components in M13 phagemid system [28]. Likewise, attempts to harness the T7 phage packaging signal have been carried out, and great success in extending the T7 phage host range was achieved by Yosef et al. [11]. However, it will be difficult to further develop the T7 packaging signal to be as powerful as the M13 phagemid because the episomal replicon impedes T7 proliferation seriously, resulting in a sharp decrease of progeny production. Unlike M13 phage, the key for exploiting T7 phage seems to lie on direct genome modification, especially, exogenous DNA knock-in. Therefore, a more efficient method for exogenous DNA insertion is required for T7-related applications development. Focus on this issue, we built the T7 in vivo integration system to simplify genetic modification of T7 phage and prompt the development of other innovative T7-related technologies for widespread utilities.

The proliferation of the modified T7 phage could be affected due to its altered gene expression regulation. Nevertheless, our engineered T7 showed negligible growth defects when maintained in pCDG10G11-containing host cells. This result indicated that genome loci including *gene 10* and *gene 11* can be excised, as the regulation of *gene 9* expression is independent of *gene 10,* and the retained T7 promoter and the downstream TΦ transcription terminator ensures efficient regulation of *gene 12* expression. Deletion of the *gene 10* and *gene 11* loci from the T7 genome provides adequate room for exogenous DNA insertion, and we demonstrated that the presence of extra DNA in this position did little harm to T7 proliferation. Besides, the successful isolation of recombinants showed that positive recombinants can be efficiently isolated via effective use of *gene 11* as a select marker. While essentially this T7 in vivo integration system draws on donor plasmids analogous to phagemids, production of recombinants is concomitant with host lysis without the need of helper phages. In summary, this T7 in vivo integration system outperforms the available T7 genome knock-in methods with eminent efficiency, and notably, this novel approach is accessible for general laboratories in varieties of applications.

As a delicate kind of prokaryotic virus bio-system, T7 phage is believed to be one of the robust nano-machines due to its fundamental capability of linking the protein functions to nucleotide information like other applied phages. It’s well known that T7 phage outperforms the temperate phages in efficiency owning to the lytic growth mode, which is an ideal property for engineering as well as industrialization. Notably, lytic phage other than T7 is seldom developed into utilitarian tools for bio-application and thus T7 remains to be the most popular lytic phage. Our in vivo integration system makes an access to take advantage of T7 phage for broader use. For example, the present phage-assisted continuous evolution (PACE) application mainly counts on M13 phage system for reason that M13 phage system is more amenable to be handled. Similar PACE application within T7 phage is now being possible to effectuate drawing on this integration system. Besides, the T7 in vivo integration strategy provides an alternative method for library construction, by which the traditional construction procedure using in vitro package can be converted to plasmids construction. Moreover, we envisage that a device capable of recording desired nucleotide information in prokaryotic system could be designed via this integration strategy. ΦC31 integrase may function as a recorder under control of specific conditions, and the desired gene as trigger would be integrated into engineered T7 phage. Different applications may occur to researchers from diverse fields, we hope this integration system could contribute to other bio-application development for variety of objectives.

We note that the scaffold of donor plasmid, which includes the kanamycin resistance gene and the P15A replicon [29], with a length of up to 1.6 kbp, is redundant in the recombinants. While this redundant insert has little effect on T7 growth, it reduces the exogenous DNA capacity of T7ΔG10G11-attB. We have attempted to remove the scaffold by preparing a minicircle DNA that contains no antibiotic marker. However, the positive ratio of candidate recombinants decreased sharply in practice due to the presence of revertants that survive in the restrictive hosts without minicircle DNA integration. Therefore, it’s necessary for this system to preserve the functional antibiotic marker and P15A replicon by which most of the bacterial cells are ensured to contain donor plasmids.

## Conclusion

We have successfully developed a T7 phage in vivo integration system mediated by ΦC31 integrase. This system allowed the rapid knock-in into an engineered T7 genome of exogenous DNA longer than 2 kbp. The limit of this system is that inserts longer than 3 kbp are unstable in the engineered T7 genome. As a novel strategy for T7 phage genome integration, this system will support further development of T7 biosystems.

## Supplementary information

**Additional file 1.** Sequences of the whole primers mentioned in the text and the supplementary materials were recorded in “Primers.xlsx”. Sequences of engineered T7 phage and functional plasmids were recorded in “Sequences of engineered T7 genome and plasmids.docx”.

**Additional file 2.** Extra information about the development of T7 in vivo integration system as well as the plasmids construction schemes were described in “Development of T7 in vivo integration system.docx” and “Plasmids construction scheme.docx”, respectively. The relevant experiment results were documented in file “Figures of supplementary materials”.

**Additional file 3.** Sequencing results of engineered T7 phage and the positive recombinants harboring the exogenous DNA were documented in three files respectively, these files comprised the crude statistics as well as the processed sequences in docx format.

## Data Availability

All data used for supporting the findings were included in this article.

## Abbreviations

attB: The bacterial attachment site recognized by ΦC31 integrase
attL: The left attachment site as a product of ΦC31 integrase recombination
attP: The phage attachment site recognized by ΦC31 integrase
attR: The right attachment site as a product of ΦC31 integrase recombination
CE: Capacity exploration
DPs: The donor plasmid used for loading the exogenous DNA
*E.coli*: *Escherichia coli*
EP: Ensemble primer pair including InsertSeq-F1 and InsertSeq-R1
EPs: The executive plasmids used for providing ΦC31 integrase
GOI: Gene of interest
MOI: Multiplicity of infection
PACE: Phage-assisted continuous evolution
PCR: Polymerase chain reaction
pfu: Phage formation unit
RBS: Ribosomal binding sequence
SP: Specific primer pair including IT-CF and IT-CR
T7∆G10G11-attB: The engineered T7 phage used for integration of exogenous DNA
T7∆G9: The engineered T7 phage used for constructing T7∆G10G11-attB
UP: Universal primer pair including Int-SP3 and G11-SR
