# Supplementary material for: A novel approach for T7 bacteriophage genome integration of exogenous DNA
Source: J Biol Eng. 2020 Jan 16;14:2. doi: 10.1186/s13036-019-0224-x (PMC6966851; doi:10.1186/s13036-019-0224-x)
Supplement: Supplementary file 1 — Additional file 1. Sequences of the whole primers mentioned in the text and the supplementary materials were recorded in “Primers.xlsx”. Sequences of engineered T7 phage and functional plasmids were recorded in “Sequences of engineered T7 genome and plasmids.docx”. [file 13036_2019_224_MOESM1_ESM.zip › Additional file 1/Sequences of engineered T7 genome and plasmids.docx]

>Expected T7∆G10G11-attB partial genome

CAGAAGACATCTCGTTCCTCCAACTGGAGAAGCAAGCAGACTTTACTGTAGCTAAAGCCGTAAGTGACGCTATCGAGGCTCGCCTTTCGTTTGCCTTTATGTTGAACTCTGCGGTTCAGCGTACAGGTGAACGTGTGACCGCCGAAGAGATTCGGTATGTAGCTTCTGAACTTGAAGATACTTTAGGTGGTGTCTACTCTATCCTTTCTCAAGAATTACAATTGCCTCTGGTACGAGTGCTCTTGAAGCAACTACAAGCCACGCAACAGATTCCTGAGTTACCTAAGGAAGCCGTAGAGCCAACCATTAGTACAGGTCTGGAAGCAATTGGTCGAGGACAAGACCTTGATAAGCTGGAGCGGTGTGTCACTGCGTGGGCTGCACTGGCACCTATGCGGGACGACCCTGATATTAACCTTGCGATGATTAAGTTACGTATTGCCAACGCTATCGGTATTGACACTTCTGGTATTCTACTCACCGAAGAACAGAAGCAACAGAAGATGGCCCAACAGTCTATGCAAATGGGTATGGATAATGGTGCTGCTGCGCTGGCTCAAGGTATGGCTGCACAAGCTACAGCTTCACCTGAGGCTATGGCTGCTGCCGCTGATTCCGTAGGTTTACAGCCGGGAATTTAATACGACTCACTATAGGGAGACCTCATCTTTGAAATGAGCGATGACAAGAGGTTGGAGTCCTCGGTCTTCCTGTAGTTCAACTTTAAGGAGACAATAATAATGGCTGAATCTAATGCAGACGTATATGCATCTTTTGGCGTGAACTCCGCTGTGATGTCTGGTGGTTCCGTTGAGGAACATGAGCAGAACATGCTGGCTCTTGATGTTGCTGCCCGTGATGGCGATGATGCAATCGAGTTAGCGTCAGACGAAGTGGAAACAGAACGTGACCTGTATGACAACTCTGACCCGTTCGGTCAAGAGGATGACGAAGGCCGCATTCAGGTTCGTATCGGTGATGGCTCTGAGCCGACCGATGTGGACACTGGAGAAGAAGGCGTTGAGGGCACCGAAGGTTCCGAAGAGTTTACCCCACTGGGCGAGACTCCAGAAGAACTGGTAGCTGCCTCTGAGCAACTTGGTGAGCACGAAGAGGGCTTCCAAGAGATGATTAACATTGCTGCTGAGCGTGGCATGAGTGTCGAGACCATTGAGGCTATCCAGCGTGAGTACGAGGAGAACGAAGAGTTGTCCGCCGAGTCCTACGCTAAGCTGGCTGAAATTGGCTACACGAAGGCTTTCATTGACTCGTATATCCGTGGTCAAGAAGCTCTGGTGGAGCAGTACGTAAACAGTGTCATTGAGTACGCTGGTGGTCGTGAACGTTTTGATGCACTGTATAACCACCTTGAGACGCACAACCCTGAGGCTGCACAGTCGCTGGATAATGCGTTGACCAATCGTGACTTAGCGACCGTTAAGGCTATCATCAACTTGGCTGGTGAGTCTCGCGCTAAGGCGTTCGGTCGTAAGCCAACTCGTAGTGTGACTAATCGTGCTATTCCGGCTAAACCTCAGGCTACCAAGCGTGAAGGCTTTGCGGACCGTAGCGAGATGATTAAAGCTATGAGTGACCCTCGGTATCGCACAGATGCCAACTATCGTCGTCAAGTCGAACAGAAAGTAATCGATTCGAACTTCTAACTAGATCTGTGCTCAAAGAGGAATCTATCAAGGGCGACACGCGAATTCGATATCAAGCTTATGTAGGTGACGGTCTCGAAGCCGCGGTGCGGGTGCCAGGGCGTGCCCTTGGGCTCCCCGGGCGCGTACTCCACCTCACCCATCTGGTCCATCATGATGAACGGGTCGGCTAGCCGAAATTAATACGACTCACTATAGGGAGACCACAACGGTTTCCCTCTAGACACTCGAGTAACTAGTTAACCCCTTGGGGCCTCTAAACGGGTCTTGAGGGGTTTTTTGCTGAAAGGAGGAACTGAGGCGAGTGTTACTTCAACCTGGTCTACTGACTCGCTAACATTAATAAATAAGGAGGCTCTAATGGCACTCATTAGCCAATCAATCAAGAACTTGAAGGGTGGTATCAGCCAACAGCCTGACATCCTTC

>Expected T7∆G9 partial genome

CAGAAGACATCTCGTTCCTCCAACTGGAGAAGCAAGCAGACTTTACTGTAGCTAAAGCCGTAAGTGACGCTATCGAGGCTCGCCTTTCGTTTGCCTTTATGTTGAACTCTGCGGTTCAGCGTACAGGTGAACGTGTGACCGCCGAAGAGATTCGGTATGTAGCTTCTGAACTTGAAGATACTTTAGGTGGTGTCTACTCTATCCTTTCTCAAGAATTACAATTGCCTCTGGTACGAGTGCTCTTGAAGCAACTACAAGCCACGCAACAGATTCCTGAGTTACCTAAGGAAGCCGTAGAGCCAACCATTAGTACAGGTCTGGAAGCAATTGGTCGAGGACAAGACCTTGATAAGCTGGAGCGGTGTGTCACTGCGTGGGCTGCACTGGCACCTATGCGGGACGACCCTGATATTAACCTTGCGATGATTAAGTTACGTATTGCCAACGCTATCGGTATTGACACTTCTGGTATTCTACTCACCGAAGAACAGAAGCAACAGAAGATGGCCCAACAGTCTATGCAAATGGGTATGGATAATGGTGCTGCTGCGCTGGCTCAAGGTATGGCTGCACAAGCTACAGCTTCACCTGAGGCTATGGCTGCTGCCGCTGATTCCGTAGGTTTACAGCCGGGAATTTAATACGACTCACTATAGGGAGACCTCATCTTTGAAATGAGCGATGACAAGAGGTTGGAGTCCTCGGTCTTCCTGTAGTTCAACTTTAAGGAGACAATAATAATGGCTGAATCTAATGCAGACGTATATGCATCTTTTGGCGTGAACTCCGCTGTGATGTCTGGTGGTTCCGTTGAGGAACATGAGCAGAACATGCTGGCTCTTGATGTTGCTGCCCGTGATGGCGATGATGCAATCgaattcatgtaggtgacggtctcgaagccgcggtgcgggtgccagggcgtgcccttgggctccccgggcgcgtactccacctcacccatctggtccatcatgatgGTCGACGCTCTCCCTTATGCGACTCCTGCATTAGGAAGCAGCCCAGTAGTAGGTTGAGGCCGTTGAGCACCGCCGCCGCAAGGAATGGTGCATGCAAGGAGATGGCGCCCAACAGTCCCCCGGCCACGGGGCCTGCCACCATACCCACGCCGAAACAAGCGCTCATGAGCCCGAAGTGGCGAGCCCGATCTTCCCCATCGGTGATGTCGGCGATATAGGCGCCAGCAACCGCACCTGTGGCGCCGGTGATGCCGGCCACGATGCGTCCGGCGTAGAGGATCCCGCGAAATTAATACGACTCACTATAGGGAGACCACAACGGTTTCCCTCTAGAAATAATTTTGTTTAACTTTAAGAAGGAGATATACATATGGCTAGCATGACTGGTGGACAGCAAATGGGTACTAACCAAGGTAAAGGTGTAGTTGCTGCTGGAGATAAACTGGCGTTGTTCTTGAAGGTATTTGGCGGTGAAGTCCTGACTGCGTTCGCTCGTACCTCCGTGACCACTTCTCGCCACATGGTACGTTCCATCTCCAGCGGTAAATCCGCTCAGTTCCCTGTTCTGGGTCGCACTCAGGCAGCGTATCTGGCTCCGGGCGAGAACCTCGACGATAAACGTAAGGACATCAAACACACCGAGAAGGTAATCACCATTGACGGTCTCCTGACGGCTGACGTTCTGATTTATGATATTGAGGACGCGATGAACCACTACGACGTTCGCTCTGAGTATACCTCTCAGTTGGGTGAATCTCTGGCGATGGCTGCGGATGGTGCGGTTCTGGCTGAGATTGCCGGTCTGTGTAACGTGGAAAGCAAATATAATGAGAACATCGAGGGCTTAGGTACTGCTACCGTAATTGAGACCACTCAGAACAAGGCCGCACTTACCGACCAAGTTGCGCTGGGTAAGGAGATTATTGCGGCTCTGACTAAGGCTCGTGCGGCTCTGACCAAGAACTATGTTCCGGCTGCTGACCGTGTGTTCTACTGTGACCCAGATAGCTACTCTGCGATTCTGGCAGCACTGATGCCGAACGCAGCAAACTACGCTGCTCTGATTGACCCTGAGAAGGGTTCTATCCGCAACGTTATGGGCTTTGAGGTTGTAGAAGTTCCGCACCTCACCGCTGGTGGTGCTGGTACCGCTCGTGAGGGCACTACTGGTCAGAAGCACGTCTTCCCTGCCAATAAAGGTGAGGGTAATGTCAAGGTTGCTAAGGACAACGTTATCGGCCTGTTCATGCACCGCTCTGCGGTAGGTACTGTTAAGCTGCGTGACTTGGCTCTGGAGCGCGCTCGCCGTGCTAACTTCCAAGCGGACCAGATTATCGCTAAGTACGCAATGGGCCACGGTGGTCTTCGCCCAGAAGCTGCTGGTGCAGTGGTTTTCAAAGTGGAGTAATGCTGGGGGTGGCCTCAACGGTCGCTGCTAGTCCCGAAGAGGCGAGTGTTACTTCAACAGAAGAAACCTTAACGCCAGCACAGGAGGCCGCACGCACCCGCGCTGCTAACAAAGCCCGAAAGGAAGCTGAGTTGGCTGCTGCCACCGCTGAGCAATAACTAGCATAACCCCTTGGGGCCTCTAAACGGGTCTTGAGGGGTTTTTTGCTGAAAGGAGGAACTATATGCGCTCATACGATATGAACGTTGAGACTGCCGCTGAGTTATCAGCTGTGAACGACATTCTGGCGTCTATCGGTGAACCTCCGGTATCAACGCTGGAAGGTGACGCTAACGCAGATGCAGCGAACGCTCGGCGTATTCTCAACAAGATTAACCGACAGATTCAATCTCGTGGATGGACGTTCAACATTGAGGAAGGCATAACGCTACTACCTGATGTTTACTCCAACCTGATTGTATACAGTGACGACTATTTATCCCTAATGTCTACTTCCGGTCAATCCATCTACGTTAACCGAGGTGGCTATGTGTATGACCGAACGAGTCAATCAGACCGCTTTGACTCTGGTATTACTGTGAACATTATTCGTCTCCGCGACTACGATGAGATGCCTGAGTGCTTCCGTTACTGGATTGTCACCAAGGCTTCCCGTCAGTTCAACAACCGATTCTTTGGGGCACCGGAAGTAGAGGGTGTACTCCAAGAAGAGGAAGATGAGGCTAGACGTCTCTGCATGGAGTATGAGATGGACTACGGTGGGTACAATATGCTGGATGGAGATGCGTTCACTTCTGGTCTACTGACTCGCTAACATTAATAAATAAGGAGGCTCTAATGGCACTCATTAGCCAATCAATCAAGAACTTGAAGGGTGGTATCAGCCAACAGCCTGACATCCTTC

>pCDG9

GATCGCTGAGATAGGTGCCTCACTGATTAAGCATTGGTAATGAGGGCCCAAATGTAATCACCTGGCTCACCTTCGGGTGGGCCTTTCTGCGTTGCTGGCGTTTTTCCATAGGCTCCGCCCCCCTGACGAGCATCACAAAAATCGATGCTCAAGTCAGAGGTGGCGAAACCCGACAGGACTATAAAGATACCAGGCGTTTCCCCCTGGAAGCTCCCTCGTGCGCTCTCCTGTTCCGACCCTGCCGCTTACCGGATACCTGTCCGCCTTTCTCCCTTCGGGAAGCGTGGCGCTTTCTCATAGCTCACGCTGTAGGTATCTCAGTTCGGTGTAGGTCGTTCGCTCCAAGCTGGGCTGTGTGCACGAACCCCCCGTTCAGCCCGACCGCTGCGCCTTATCCGGTAACTATCGTCTTGAGTCCAACCCGGTAAGACACGACTTATCGCCACTGGCAGCAGCCACTGGTAACAGGATTAGCAGAGCGAGGTATGTAGGCGGTGCTACAGAGTTCTTGAAGTGGTGGCCTAACTACGGCTACACTAGAAGAACAGTATTTGGTATCTGCGCTCTGCTGAAGCCAGTTACCTCGGAAAAAGAGTTGGTAGCTCTTGATCCGGCAAACAAACCACCGCTGGTAGCGGTGGTTTTTTTGTTTGCAAGCAGCAGATTACGCGCAGAAAAAAAGGATCTCAAGAAGATCCTTTGATTTTCTACCGAAGAAAGGCCCACCCGTGAAGGTGAGCCAGTGAGTTGATTGTGTAAAACGACGGCCAGTGTCTGAGGCTCGCTGCAGTCCTGAAGCTTGATATCGAATTCGCGTGTCGCCCTTTAATACGACTCACTATAGGGAGACCTCATCTTTGAAATGAGCGATGACAAGAGGTTGGAGTCCTCGGTCTTCCTGTAGTTCAACTTTAAGGAGACAATAATAATGGCTGAATCTAATGCAGACGTATATGCATCTTTTGGCGTGAACTCCGCTGTGATGTCTGGTGGTTCCGTTGAGGAACATGAGCAGAACATGCTGGCTCTTGATGTTGCTGCCCGTGATGGCGATGATGCAATCGAGTTAGCGTCAGACGAAGTGGAAACAGAACGTGACCTGTATGACAACTCTGACCCGTTCGGTCAAGAGGATGACGAAGGCCGCATTCAGGTTCGTATCGGTGATGGCTCTGAGCCGACCGATGTGGACACTGGAGAAGAAGGCGTTGAGGGCACCGAAGGTTCCGAAGAGTTTACCCCACTGGGCGAGACTCCAGAAGAACTGGTAGCTGCCTCTGAGCAACTTGGTGAGCACGAAGAGGGCTTCCAAGAGATGATTAACATTGCTGCTGAGCGTGGCATGAGTGTCGAGACCATTGAGGCTATCCAGCGTGAGTACGAGGAGAACGAAGAGTTGTCCGCCGAGTCCTACGCTAAGCTGGCTGAAATTGGCTACACGAAGGCTTTCATTGACTCGTATATCCGTGGTCAAGAAGCTCTGGTGGAGCAGTACGTAAACAGTGTCATTGAGTACGCTGGTGGTCGTGAACGTTTTGATGCACTGTATAACCACCTTGAGACGCACAACCCTGAGGCTGCACAGTCGCTGGATAATGCGTTGACCAATCGTGACTTAGCGACCGTTAAGGCTATCATCAACTTGGCTGGTGAGTCTCGCGCTAAGGCGTTCGGTCGTAAGCCAACTCGTAGTGTGACTAATCGTGCTATTCCGGCTAAACCTCAGGCTACCAAGCGTGAAGGCTTTGCGGACCGTAGCGAGATGATTAAAGCTATGAGTGACCCTCGGTATCGCACAGATGCCAACTATCGTCGTCAAGTCGAACAGAAAGTAATCGATTCGAACTTCTAACTAGATCTGTGCTCAAAGAGGAATCTATCAAGGGCGACACGCGAATTCGATATCGCGGCCGCCTGCAGTCAATACTGACGATGGTCATAGCTGTTTCCTGTCCATAGCAGAAAGTCAAAAGCCTCCGACCGGAGGCTTTTGACTTGATCGGCACGTAAGAGGTTCCAACTTTCACCATAATGAAATAAGATCACTACCGGGCGTATTTTTTGAGTTATCGAGATTTTCAGGAGCTAAGGAAGCTAAAATGAGTATTCAACATTTCCGTGTCGCACTTATTCCGTTTTTTGCGGCATTTTGCCTTCCTGTTTTTGCTCACCCAGAAACGCTGGTGAAAGTAAAAGATGCTGAAGATCAGTTGGGTGCACGAGTGGGTTACATCGAACTGGATCTCAACAGCGGTAAGATCCTTGAGAGTTTTCGCCCCGAAGAACGTTTTCCAATGATGAGCACTTTTAAAGTTCTGCTATGTGGCGCGGTATTATCCCGTATTGACGCCGGGCAAGAGCAACTCGGTCGCCGCATACACTATTCTCAGAATGACTTGGTTGAGTACTCACCAGTCACAGAAAAGCATCTTACGGATGGCATGACAGTAAGAGAATTATGCAGTGCTGCCATAACCATGAGTGATAACACTGCGGCCAACTTACTTCTGACAACGATCGGAGGACCGAAGGAGCTAACCGCTTTTTTGCACAACATGGGGGATCATGTAACTCGCCTTGATCGTTGGGAACCGGAGCTGAATGAAGCCATACCAAACGACGAGCGTGACACCACGATGCCTGTAGCAATGGCAACAACGTTGCGCAAACTATTAACTGGCGAACTACTTACTCTAGCTTCCCGGCAACAATTAATAGACTGGATGGAGGCGGATAAAGTTGCAGGACCACTTCTGCGCTCGGCCCTTCCGGCTGGCTGGTTTATTGCTGATAAATCTGGAGCCGGTGAGCGTGGGTCTCGCGGTATCATTGCAGCACTGGGGCCAGATGGTAAGCCCTCCCGTATCGTAGTTATCTACACGACGGGGAGTCAGGCAACTATGGATGAACGAAATAGACA

>pCDG10

GATCGCTGAGATAGGTGCCTCACTGATTAAGCATTGGTAATGAGGGCCCAAATGTAATCACCTGGCTCACCTTCGGGTGGGCCTTTCTGCGTTGCTGGCGTTTTTCCATAGGCTCCGCCCCCCTGACGAGCATCACAAAAATCGATGCTCAAGTCAGAGGTGGCGAAACCCGACAGGACTATAAAGATACCAGGCGTTTCCCCCTGGAAGCTCCCTCGTGCGCTCTCCTGTTCCGACCCTGCCGCTTACCGGATACCTGTCCGCCTTTCTCCCTTCGGGAAGCGTGGCGCTTTCTCATAGCTCACGCTGTAGGTATCTCAGTTCGGTGTAGGTCGTTCGCTCCAAGCTGGGCTGTGTGCACGAACCCCCCGTTCAGCCCGACCGCTGCGCCTTATCCGGTAACTATCGTCTTGAGTCCAACCCGGTAAGACACGACTTATCGCCACTGGCAGCAGCCACTGGTAACAGGATTAGCAGAGCGAGGTATGTAGGCGGTGCTACAGAGTTCTTGAAGTGGTGGCCTAACTACGGCTACACTAGAAGAACAGTATTTGGTATCTGCGCTCTGCTGAAGCCAGTTACCTCGGAAAAAGAGTTGGTAGCTCTTGATCCGGCAAACAAACCACCGCTGGTAGCGGTGGTTTTTTTGTTTGCAAGCAGCAGATTACGCGCAGAAAAAAAGGATCTCAAGAAGATCCTTTGATTTTCTACCGAAGAAAGGCCCACCCGTGAAGGTGAGCCAGTGAGTTGATTGTGTAAAACGACGGCCAGTGTCTGAGGCTCGCTGCAGTCCTGAAGCTTGATATCGAATTCGCGTGTCGCCCTTCGCGAATTCGATATCAAGCTTTTACTCCACTTTGAAAACCACTGCACCAGCAGCTTCTGGGCGAAGACCACCGTGGCCCATTGCGTACTTAGCGATAATCTGGTCCGCTTGGAAGTTAGCACGGCGAGCGCGCTCCAGAGCCAAGTCACGCAGCTTAACAGTACCTACCGCAGAGCGGTGCATGAACAGGCCGATAACGTTGTCCTTAGCAACCTTGACATTACCCTCACCTTTATTGGCAGGGAAGACGTGCTTCTGACCAGTAGTGCCCTCACGAGCGGTACCAGCACCACCAGCGGTGAGGTGCGGAACTTCTACAACCTCAAAGCCCATAACGTTGCGGATAGAACCCTTCTCAGGGTCAATCAGAGCAGCGTAGTTTGCTGCGTTCGGCATCAGTGCTGCCAGAATCGCAGAGTAGCTATCTGGGTCACAGTAGAACACACGGTCAGCAGCCGGAACATAGTTCTTGGTCAGAGCCGCACGAGCCTTAGTCAGAGCCGCAATAATCTCCTTACCCAGCGCAACTTGGTCGGTAAGTGCGGCCTTGTTCTGAGTGGTCTCAATTACGGTAGCAGTACCTAAGCCCTCGATGTTCTCATTATATTTGCTTTCCACGTTACACAGACCGGCAATCTCAGCCAGAACCGCACCATCCGCAGCCATCGCCAGAGATTCACCCAACTGAGAGGTATACTCAGAGCGAACGTCGTAGTGGTTCATCGCGTCCTCAATATCATAAATCAGAACGTCAGCCGTCAGGAGACCGTCAATGGTGATTACCTTCTCGGTGTGTTTGATGTCCTTACGTTTATCGTCGAGGTTCTCGCCCGGAGCCAGATACGCTGCCTGAGTGCGACCCAGAACAGGGAACTGAGCGGATTTACCGCTGGAGATGGAACGTACCATGTGGCGAGAAGTGGTCACGGAGGTACGAGCGAACGCAGTCAGGACTTCACCGCCAAATACCTTCAAGAACAACGCCAGTTTATCTCCAGCAGCAACTACACCTTTACCTTGGTTAGTACCCATTTGCTGTCCACCAGTCATGCTAGCCATATGTATATCTCCTTCTTAAAGTTAAACAAAATTATTTCTAGAGGGAAACCGTTGTGGTCTCCCTATAGTGAGTCGTATTAATTTCGAAGTCTATCAGAAGTTCGAATCGATTACTTTCTGTTCGACTTGACGACGATAGTTGGCATCTGTGCGATACCGAGGGTCACTCATAGCTTTAATCATCTCGCTACGGTCCGAAGCTTCAGGACTGCAGCGAGAAGGGCGACACGCGAATTCGATATCGCGGCCGCCTGCAGTCAATACTGACGATGGTCATAGCTGTTTCCTGTCCATAGCAGAAAGTCAAAAGCCTCCGACCGGAGGCTTTTGACTTGATCGGCACGTAAGAGGTTCCAACTTTCACCATAATGAAATAAGATCACTACCGGGCGTATTTTTTGAGTTATCGAGATTTTCAGGAGCTAAGGAAGCTAAAATGAGTATTCAACATTTCCGTGTCGCACTTATTCCGTTTTTTGCGGCATTTTGCCTTCCTGTTTTTGCTCACCCAGAAACGCTGGTGAAAGTAAAAGATGCTGAAGATCAGTTGGGTGCACGAGTGGGTTACATCGAACTGGATCTCAACAGCGGTAAGATCCTTGAGAGTTTTCGCCCCGAAGAACGTTTTCCAATGATGAGCACTTTTAAAGTTCTGCTATGTGGCGCGGTATTATCCCGTATTGACGCCGGGCAAGAGCAACTCGGTCGCCGCATACACTATTCTCAGAATGACTTGGTTGAGTACTCACCAGTCACAGAAAAGCATCTTACGGATGGCATGACAGTAAGAGAATTATGCAGTGCTGCCATAACCATGAGTGATAACACTGCGGCCAACTTACTTCTGACAACGATCGGAGGACCGAAGGAGCTAACCGCTTTTTTGCACAACATGGGGGATCATGTAACTCGCCTTGATCGTTGGGAACCGGAGCTGAATGAAGCCATACCAAACGACGAGCGTGACACCACGATGCCTGTAGCAATGGCAACAACGTTGCGCAAACTATTAACTGGCGAACTACTTACTCTAGCTTCCCGGCAACAATTAATAGACTGGATGGAGGCGGATAAAGTTGCAGGACCACTTCTGCGCTCGGCCCTTCCGGCTGGCTGGTTTATTGCTGATAAATCTGGAGCCGGTGAGCGTGGGTCTCGCGGTATCATTGCAGCACTGGGGCCAGATGGTAAGCCCTCCCGTATCGTAGTTATCTACACGACGGGGAGTCAGGCAACTATGGATGAACGAAATAGACA

>pCDG10G11

TCGCGCGTTTCGGTGATGACGGTGAAAACCTCTGACACATGCAGCTCCCGGAGACGGTCACAGCTTGTCTGTAAGCGGATGCCGGGAGCAGACAAGCCCGTCAGGGCGCGTCAGCGGGTGTTGGCGGGTGTCGGGGCTGGCTTAACTATGCGGCATCAGAGCAGATTGTACTGAGAGTGCACCATATGCGGTGTGAAATACCGCACAGATGCGTAAGGAGAAAATACCGCATCAGGCGCCATTCGCCATTCAGGCTGCGCAACTGTTGGGAAGGGCGATCGGTGCGGGCCTCTTCGCTATTACGCCAGCTGGCGAAAGGGGGATGTGCTGCAAGGCGATTAAGTTGGGTAACGCCAGGGTTTTCCCAGTCACGACGTTGTAAAACGACGGCCAGTGAATTCTAGGGATAACAGGGTAATCATTAGGAAGCAGCCCAGTCTCGAGTTAATACGACTCACTATAGGGAGACCACAACGGTTTCCCTCTAGAAATAATTTTGTTTAACTTTAAGAAGGAGATATACATATGGCTAGCATGACTGGTGGACAGCAAATGGGTACTAACCAAGGTAAAGGTGTAGTTGCTGCTGGAGATAAACTGGCGTTGTTCTTGAAGGTATTTGGCGGTGAAGTCCTGACTGCGTTCGCTCGTACCTCCGTGACCACTTCTCGCCACATGGTACGTTCCATCTCCAGCGGTAAATCCGCTCAGTTCCCTGTTCTGGGTCGCACTCAGGCAGCGTATCTGGCTCCGGGCGAGAACCTCGACGATAAACGTAAGGACATCAAACACACCGAGAAGGTAATCACCATTGACGGTCTCCTGACGGCTGACGTTCTGATTTATGATATTGAGGACGCGATGAACCACTACGACGTTCGCTCTGAGTATACCTCTCAGTTGGGTGAATCTCTGGCGATGGCTGCGGATGGTGCGGTTCTGGCTGAGATTGCCGGTCTGTGTAACGTGGAAAGCAAATATAATGAGAACATCGAGGGCTTAGGTACTGCTACCGTAATTGAGACCACTCAGAACAAGGCCGCACTTACCGACCAAGTTGCGCTGGGTAAGGAGATTATTGCGGCTCTGACTAAGGCTCGTGCGGCTCTGACCAAGAACTATGTTCCGGCTGCTGACCGTGTGTTCTACTGTGACCCAGATAGCTACTCTGCGATTCTGGCAGCACTGATGCCGAACGCAGCAAACTACGCTGCTCTGATTGACCCTGAGAAGGGTTCTATCCGCAACGTTATGGGCTTTGAGGTTGTAGAAGTTCCGCACCTCACCGCTGGTGGTGCTGGTACCGCTCGTGAGGGCACTACTGGTCAGAAGCACGTCTTCCCTGCCAATAAAGGTGAGGGTAATGTCAAGGTTGCTAAGGACAACGTTATCGGCCTGTTCATGCACCGCTCTGCGGTAGGTACTGTTAAGCTGCGTGACTTGGCTCTGGAGCGCGCTCGCCGTGCTAACTTCCAAGCGGACCAGATTATCGCTAAGTACGCAATGGGCCACGGTGGTCTTCGCCCAGAAGCTGCTGGTGCAGTGGTTTTCAAAGTGGAGTAACTCGAGAGTAGGTTGAGGCCGTTGAGCTAGGGATAACAGGGTAATTCCCCATCGGTGATGTCGCGGACCGTAACCCCTTGGGGCCTCTAAACGGGTCTTGAGGGGTTTTTTGCTGAAAGGAGGAACTATATGCGCTCATACGATATGAACGTTGAGACTGCCGCTGAGTTATCAGCTGTGAACGACATTCTGGCGTCTATCGGTGAACCTCCGGTATCAACGCTGGAAGGTGACGCTAACGCAGATGCAGCGAACGCTCGGCGTATTCTCAACAAGATTAACCGACAGATTCAATCTCGTGGATGGACGTTCAACATTGAGGAAGGCATAACGCTACTACCTGATGTTTACTCCAACCTGATTGTATACAGTGACGACTATTTATCCCTAATGTCTACTTCCGGTCAATCCATCTACGTTAACCGAGGTGGCTATGTGTATGACCGAACGAGTCAATCAGACCGCTTTGACTCTGGTATTACTGTGAACATTATTCGTCTCCGCGACTACGATGAGATGCCTGAGTGCTTCCGTTACTGGATTGTCACCAAGGCTTCCCGTCAGTTCAACAACCGATTCTTTGGGGCACCGGAAGTAGAGGGTGTACTCCAAGAAGAGGAAGATGAGGCTAGACGTCTCTGCATGGAGTATGAGATGGACTACGGTGGGTACAATATGCTGGATGGAGATGCGTTCACTTCTGGTCTACTGACTCGCTAACGGACCGGCGATATAGGCGCCAGCAACCGTAGGGATAACAGGGTAATGATGTCGGCGATATAGGCGGCTAGCCCAGCAACCGCACCTGTGGCGTAGGGATAACAGGGTAATGTCGACTGCAGAGGCCTGCATGCAAGCTTGGCGTAATCATGGTCATAGCTGTTTCCTGTGTGAAATTGTTATCCGCTCACAATTCCACACAACATACGAGCCGGAAGCATAAAGTGTAAAGCCTGGGGTGCCTAATGAGTGAGCTAACTCACATTAATTGCGTTGCGCTCACTGCCCGCTTTCCAGTCGGGAAACCTGTCGTGCCAGCTGCATTAATGAATCGGCCAACGCGCGGGGAGAGGCGGTTTGCGTATTGGGCGCTCTTCCGCTTCCTCGCTCACTGACTCGCTGCGCTCGGTCGTTCGGCTGCGGCGAGCGGTATCAGCTCACTCAAAGGCGGTAATACGGTTATCCACAGAATCAGGGGATAACGCAGGAAAGAACATGTGAGCAAAAGGCCAGCAAAAGGCCAGGAACCGTAAAAAGGCCGCGTTGCTGGCGTTTTTCCATAGGCTCCGCCCCCCTGACGAGCATCACAAAAATCGACGCTCAAGTCAGAGGTGGCGAAACCCGACAGGACTATAAAGATACCAGGCGTTTCCCCCTGGAAGCTCCCTCGTGCGCTCTCCTGTTCCGACCCTGCCGCTTACCGGATACCTGTCCGCCTTTCTCCCTTCGGGAAGCGTGGCGCTTTCTCATAGCTCACGCTGTAGGTATCTCAGTTCGGTGTAGGTCGTTCGCTCCAAGCTGGGCTGTGTGCACGAACCCCCCGTTCAGCCCGACCGCTGCGCCTTATCCGGTAACTATCGTCTTGAGTCCAACCCGGTAAGACACGACTTATCGCCACTGGCAGCAGCCACTGGTAACAGGATTAGCAGAGCGAGGTATGTAGGCGGTGCTACAGAGTTCTTGAAGTGGTGGCCTAACTACGGCTACACTAGAAGAACAGTATTTGGTATCTGCGCTCTGCTGAAGCCAGTTACCTTCGGAAAAAGAGTTGGTAGCTCTTGATCCGGCAAACAAACCACCGCTGGTAGCGGTGGTTTTTTTGTTTGCAAGCAGCAGATTACGCGCAGAAAAAAAGGATCTCAAGAAGATCCTTTGATCTTTTCTACGGGGTCTGACGCTCAGTGGAACGAAAACTCACGTTAAGGGATTTTGGTCATGAGATTATCAAAAAGGATCTTCACCTAGATCCTTTTAAATTAAAAATGAAGTTTTAAATCAATCTAAAGTATATATGAGTAAACTTGGTCTGACAGTTACCAATGCTTAATCAGTGAGGCACCTATCTCAGCGATCTGTCTATTTCGTTCATCCATAGTTGCCTGACTCCCCGTCGTGTAGATAACTACGATACGGGAGGGCTTACCATCTGGCCCCAGTGCTGCAATGATACCGCGACTCCCACGCTCACCGGCTCCAGATTTATCAGCAATAAACCAGCCAGCCGGAAGGGCCGAGCGCAGAAGTGGTCCTGCAACTTTATCCGCCTCCATCCAGTCTATTAATTGTTGCCGGGAAGCTAGAGTAAGTAGTTCGCCAGTTAATAGTTTGCGCAACGTTGTTGCCATTGCTACAGGCATCGTGGTGTCACGCTCGTCGTTTGGTATGGCTTCATTCAGCTCCGGTTCCCAACGATCAAGGCGAGTTACATGATCCCCCATGTTGTGCAAAAAAGCGGTTAGCTCCTTCGGTCCTCCGATCGTTGTCAGAAGTAAGTTGGCCGCAGTGTTATCACTCATGGTTATGGCAGCACTGCATAATTCTCTTACTGTCATGCCATCCGTAAGATGCTTTTCTGTGACTGGTGAGTACTCAACCAAGTCATTCTGAGAATAGTGTATGCGGCGACCGAGTTGCTCTTGCCCGGCGTCAATACGGGATAATACCGCGCCACATAGCAGAACTTTAAAAGTGCTCATCATTGGAAAACGTTCTTCGGGGCGAAAACTCTCAAGGATCTTACCGCTGTTGAGATCCAGTTCGATGTAACCCACTCGTGCACCCAACTGATCTTCAGCATCTTTTACTTTCACCAGCGTTTCTGGGTGAGCAAAAACAGGAAGGCAAAATGCCGCAAAAAAGGGAATAAGGGCGACACGGAAATGTTGAATACTCATACTCTTCCTTTTTCAATATTATTGAAGCATTTATCAGGGTTATTGTCTCATGAGCGGATACATATTTGAATGTATTTAGAAAAATAAACAAATAGGGGTTCCGCGCACATTTCCCCGAAAAGTGCCACCTGACGTCTAAGAAACCATTATTATCATGACATTAACCTATAAAAATAGGCGTATCACGAGGCCCTTTCGTC

>pRFG9 partial sequence

CAAGGGTTGGTTTGCGGCATTCACAGTTCTCGCAAGAATGATGGCTCATCTGAGTGTGATCCGTAGCGAGTGCGCGCTTCCATTCAGGTCGAGGTGGCCCGGCTCCATGCACCGCGACGCAACGCGGGGAGGCAGACAAGGTATAGGGCGGCGCCTACAATCCATGCCAACCCGTTCCATGTGCTCGCCGAGGCGGCATAAATCGCCGTGACGATCAGCGGTCCAATGATCGAAGTTAGGCTGGTAAGAGCCGCGAGCGATCCTTGAAGCTGTCCCTGATGGTCGTCATCTACCTGCCTGGACAGCATGGCCTGCAACGCGGGCATCCCGATGCCGCCGGAAGCGAGAAGAATCATAATGGGGAAGGCCATCCAGCCTCGCGTCGCGAACGCCAGCAAGACGTAGCCCAGCGCGTCGGCCGCCATGCCGGCGATAATGGCCTGCTTCTCGCCGAAACGTTTGGTGGCGGGACCAGTGACGAAGGCTTGAGCGAGGGCGTGCAAGATTCCGAATACCGCAAGCGACAGGCCGATCATCGTCGCGCTCCAGCGAAAGCGGTCCTCGCCGAAAATGACCCAGAGCGCTGCCGGCACCTGTCCTACGAGTTGCATGATAAAGAAGACAGTCATAAGTGCGGCGACGATAGTCATGCCCCGCGCCCACCGGAAGGAGCTGACTGGGTTGAAGGCTCTCAAGGGCATCGGTCGACTTGGTCGAGGACAAGACCTTGATAAGCTGGAGCGGTGTGTCACTGCGTGGGCTGCACTGGCACCTATGCGGGACGACCCTGATATTAACCTTGCGATGATTAAGTTACGTATTGCCAACGCTATCGGTATTGACACTTCTGGTATTCTACTCACCGAAGAACAGAAGCAACAGAAGATGGCCCAACAGTCTATGCAAATGGGTATGGATAATGGTGCTGCTGCGCTGGCTCAAGGTATGGCTGCACAAGCTACAGCTTCACCTGAGGCTATGGCTGCTGCCGCTGATTCCGTAGGTTTACAGCCGGGAATTTAATACGACTCACTATAGGGAGACCTCATCTTTGAAATGAGCGATGACAAGAGGTTGGAGTCCTCGGTCTTCCTGTAGTTCAACTTTAAGGAGACAATAATAATGGCTGAATCTAATGCAGACGTATATGCATCTTTTGGCGTGAACTCCGCTGTGATGTCTGGTGGTTCCGTTGAGGAACATGAGCAGAACATGCTGGCTCTTGATGTTGCTGCCCGTGATGGCGATGATGCAATCgaattcatgtaggtgacggtctcgaagccgcggtgcgggtgccagggcgtgcccttgggctccccgggcgcgtactccacctcacccatctggtccatcatgatgGTCGACGCTCTCCCTTATGCGACTCCTGCATTAGGAAGCAGCCCAGTAGTAGGTTGAGGCCGTTGAGCACCGCCGCCGCAAGGAATGGTGCATGCAAGGAGATGGCGCCCAACAGTCCCCCGGCCACGGGGCCTGCCACCATACCCACGCCGAAACAAGCGCTCATGAGCCCGAAGTGGCGAGCCCGATCTTCCCCATCGGTGATGTCGGCGATATAGGCGCCAGCAACCGCACCTGTGGCGCCGGTGATGCCGGCCACGATGCGTCCGGCGTAGAGGATCCCGCGAAATTAATACGACTCACTATAGGGAGACCACAACGGTTTCCCTCTAGAAATAATTTTGTTTAACTTTAAGAAGGAGATATACATATGGCTAGCATGACTGGTGGACAGCAAATGGGTACTAACCAAGGTAAAGGTGTAGTTGCTGCTGGAGATAAACTGGCGTTGTTCTTGAAGGTATTTGGCGGTGAAGTCCTGACTGCGTTCGCTCGTACCTCCGTGACCACTTCTCGCCACATGGTACGTTCCATCTCCAGCGGTAAATCCGCTCAGTTCCCTGTTCTGGGTCGCACTCAGGCAGCGTATCTGGCTCCGGGCGAGAACCTCGACGATAAACGTAAGGACATCAAACACACCGAGAAGGTAATCACCATTGACGGTCTCCTGACGGCTGACGTTCTGATTTATGATATTGAGGACGCGATGAACCACTACGACGTTCGCTCTGAGTATACCTCTCAGTTGGGTGAATCTCTGGCGATGGCTGCGGATGGTGCGGTTCTGGCTGAGATTGCCGGTCTGTGTAACGTGGAAAGCAAATATAATGAGAACATCGAGGGCTTAGGTACTGCTACCGTAATTGAGACCACTCAGAACAAGGCCGCACTTACCGACCAAGTTGCGCTGGGTAAGGAGATTATTGCGGCTCTGACTAAGGCTCGTGCGGCTCTGACCAAGAACTATGTTCCGGCTGCTGACCGTGTGTTCTACTGTGACCCAGATAGCTACTCTGCGATTCTGGCAGCACTGATGCCGAACGCAGCAAACTACGCTGCTCTGATTGACCCTGAGAAGGGTTCTATCCGCAACGTTATGGGCTTTGAGGTTGTAGAAGTTCCGCACCTCACCGCTGGTGGTGCTGGTACCGCTCGTGAGGGCACTACTGGTCAGAAGCACGTCTTCCCTGCCAATAAAGGTGAGGGTAATGTCAAGGTTGCTAAGGACAACGTTATCGGCCTGTTCATGCACCGCTCTGCGGTAGGTACTGTTAAGCTGCGTGACTTGGCTCTGGAGCGCGCTCGCCGTGCTAACTTCCAAGCGGACCAGATTATCGCTAAGTACGCAATGGGCCACGGTGGTCTTCGCCCAGAAGCTGCTGGTGCAGTGGTTTTCAAAGTGGAGTAAGAATTCTTGAAGACGAAAGGGCCTCGTGATACGCCTATTTTTATAGGTTAATGTCATGATAATAATGGTTTCTTAGACGTCAGGTGGCACTTTTCGGGGAAATGTGCGCGGAACCCCTATTTGTTTATTTTTCTAAATACATTCAAATATGTATCCGCTCATGAGACAATAACCCTGATAAATGCTTCAATAATATTGAAAAAGGAAGAGTATGAGTATTCAACATTTCCGTGTCGCCCTTATTCCCTTTTTTGCGGCATTTTGCCTTCCTGTTTTTGCTCACCCAGAAACGCTGGTGAAAGTAAAAGATGCTGAAGATCAGTTGGGTGCACGAGTGGGTTACATCGAACTGGATCTCAACAGCGGTAAGATCCTTGAGAGTTTTCGCCCCGAAGAACGTTTTCCAATGATGAGCACTTTTAAAGTTCTGCTATGTGGCGCGGTATTATCCCGTGTTGACGCCGGGCAAGAGCAACTCGGTCGCCGCATACACTATTCTCAGAATGACTTGGTTGAGTACTCACCAGTCACAGAAAAGCATCTTACGGATGGCATGACAGTAAGAGAATTATGCAGTGCTGCCATAACCATGAGTGATAACACTGCGGCCAACTTACTTCTGACAACGATCGGAGGACCGAAGGAGCTAACCGCTTTTTTGCACAACATGGGGGATCATGTAACTCGCCTTGATCGTTGGGAACCGGAGCTGAATGAAGCCATACCAAACGACGAGCGTGACACCACGATGCCTGCAGCAATGGCAACAACGTTGCGCAAACTATTAACTGGCGAACTACTTACTCTAGCTTCCCGGCAACAATTAATAGACTGGATGGAGGCGGATAAAGTTGCAGGACCACTTCTGCGCTCGGCCCTTCCGGCTGGCTGGTTTATTGCTGATAAATCTGGAGCCGGTGAGCGTGGGTCTCGCGGTATCATTGCAGCACTGGGGCCAGATGGTAAGCCCTCCCGTATCGTAGTTATCTACACGACGGGGAGTCAGGCACTATGGATGACGAAATAGACAGATCGCTGAGATAGGTGCCTCACTGATAGCATGTTACTGTCAGACAGTTTACTCATATAATACTTTAGATTGATTGAC

>pRFG10G11

GATCGCTGAGATAGGTGCCTCACTGATTAAGCATTGGTAATGAGGGCCCAAATGTAATCACCTGGCTCACCTTCGGGTGGGCCTTTCTGCGTTGCTGGCGTTTTTCCATAGGCTCCGCCCCCCTGACGAGCATCACAAAAATCGATGCTCAAGTCAGAGGTGGCGAAACCCGACAGGACTATAAAGATACCAGGCGTTTCCCCCTGGAAGCTCCCTCGTGCGCTCTCCTGTTCCGACCCTGCCGCTTACCGGATACCTGTCCGCCTTTCTCCCTTCGGGAAGCGTGGCGCTTTCTCATAGCTCACGCTGTAGGTATCTCAGTTCGGTGTAGGTCGTTCGCTCCAAGCTGGGCTGTGTGCACGAACCCCCCGTTCAGCCCGACCGCTGCGCCTTATCCGGTAACTATCGTCTTGAGTCCAACCCGGTAAGACACGACTTATCGCCACTGGCAGCAGCCACTGGTAACAGGATTAGCAGAGCGAGGTATGTAGGCGGTGCTACAGAGTTCTTGAAGTGGTGGCCTAACTACGGCTACACTAGAAGAACAGTATTTGGTATCTGCGCTCTGCTGAAGCCAGTTACCTCGGAAAAAGAGTTGGTAGCTCTTGATCCGGCAAACAAACCACCGCTGGTAGCGGTGGTTTTTTTGTTTGCAAGCAGCAGATTACGCGCAGAAAAAAAGGATCTCAAGAAGATCCTTTGATTTTCTACCGAAGAAAGGCCCACCCGTGAAGGTGAGCCAGTGAGTTGATTGTGTAAAACGACGGCCAGTGTCTGAGGCTCGCTGCAGTCCTGAAGCTTGATATCGAATTCGCGTGTCGCCCTTTAATACGACTCACTATAGGGAGACCTCATCTTTGAAATGAGCGATGACAAGAGGTTGGAGTCCTCGGTCTTCCTGTAGTTCAACTTTAAGGAGACAATAATAATGGCTGAATCTAATGCAGACGTATATGCATCTTTTGGCGTGAACTCCGCTGTGATGTCTGGTGGTTCCGTTGAGGAACATGAGCAGAACATGCTGGCTCTTGATGTTGCTGCCCGTGATGGCGATGATGCAATCGAGTTAGCGTCAGACGAAGTGGAAACAGAACGTGACCTGTATGACAACTCTGACCCGTTCGGTCAAGAGGATGACGAAGGCCGCATTCAGGTTCGTATCGGTGATGGCTCTGAGCCGACCGATGTGGACACTGGAGAAGAAGGCGTTGAGGGCACCGAAGGTTCCGAAGAGTTTACCCCACTGGGCGAGACTCCAGAAGAACTGGTAGCTGCCTCTGAGCAACTTGGTGAGCACGAAGAGGGCTTCCAAGAGATGATTAACATTGCTGCTGAGCGTGGCATGAGTGTCGAGACCATTGAGGCTATCCAGCGTGAGTACGAGGAGAACGAAGAGTTGTCCGCCGAGTCCTACGCTAAGCTGGCTGAAATTGGCTACACGAAGGCTTTCATTGACTCGTATATCCGTGGTCAAGAAGCTCTGGTGGAGCAGTACGTAAACAGTGTCATTGAGTACGCTGGTGGTCGTGAACGTTTTGATGCACTGTATAACCACCTTGAGACGCACAACCCTGAGGCTGCACAGTCGCTGGATAATGCGTTGACCAATCGTGACTTAGCGACCGTTAAGGCTATCATCAACTTGGCTGGTGAGTCTCGCGCTAAGGCGTTCGGTCGTAAGCCAACTCGTAGTGTGACTAATCGTGCTATTCCGGCTAAACCTCAGGCTACCAAGCGTGAAGGCTTTGCGGACCGTAGCGAGATGATTAAAGCTATGAGTGACCCTCGGTATCGCACAGATGCCAACTATCGTCGTCAAGTCGAACAGAAAGTAATCGATTCGAACTTCTAACTAGATCTGTGCTCAAAGAGGAATCTATCAAGGGCGACACGCGAATTCGATATCGCGGCCGCCGATATCAAGCTTATGTAGGTGACGGTCTCGAAGCCGCGGTGCGGGTGCCAGGGCGTGCCCTTGGGCTCCCCGGGCGCGTACTCCACCTCACCCATCTGGTCCATCATGATGAACGGGTCGGCTAGCCGAAATTAATACGACTCACTATAGGGAGACCACAACGGTTTCCCTCTAGACACTCGAGTAACTAGTTAACCCCTTGGGGCCTCTAAACGGGTCTTGAGGGGTTTTTTGCTGAAAGGAGGAACTGAGGCGAGTGTTACTTCAACCTGGTCTACTGACTCGCTAACATTAATAAATAAGGAGGCTCTAATGGCACTCATTAGCCAATCAATCAAGAACTTGAAGGGTGGTATCAGCCAACAGCCTGACATCCTTCGCGGCCGCCTGCAGTCAATACTGACGATGGTCATAGCTGTTTCCTGTCCATAGCAGAAAGTCAAAAGCCTCCGACCGGAGGCTTTTGACTTGATCGGCACGTAAGAGGTTCCAACTTTCACCATAATGAAATAAGATCACTACCGGGCGTATTTTTTGAGTTATCGAGATTTTCAGGAGCTAAGGAAGCTAAAATGAGTATTCAACATTTCCGTGTCGCACTTATTCCGTTTTTTGCGGCATTTTGCCTTCCTGTTTTTGCTCACCCAGAAACGCTGGTGAAAGTAAAAGATGCTGAAGATCAGTTGGGTGCACGAGTGGGTTACATCGAACTGGATCTCAACAGCGGTAAGATCCTTGAGAGTTTTCGCCCCGAAGAACGTTTTCCAATGATGAGCACTTTTAAAGTTCTGCTATGTGGCGCGGTATTATCCCGTATTGACGCCGGGCAAGAGCAACTCGGTCGCCGCATACACTATTCTCAGAATGACTTGGTTGAGTACTCACCAGTCACAGAAAAGCATCTTACGGATGGCATGACAGTAAGAGAATTATGCAGTGCTGCCATAACCATGAGTGATAACACTGCGGCCAACTTACTTCTGACAACGATCGGAGGACCGAAGGAGCTAACCGCTTTTTTGCACAACATGGGGGATCATGTAACTCGCCTTGATCGTTGGGAACCGGAGCTGAATGAAGCCATACCAAACGACGAGCGTGACACCACGATGCCTGTAGCAATGGCAACAACGTTGCGCAAACTATTAACTGGCGAACTACTTACTCTAGCTTCCCGGCAACAATTAATAGACTGGATGGAGGCGGATAAAGTTGCAGGACCACTTCTGCGCTCGGCCCTTCCGGCTGGCTGGTTTATTGCTGATAAATCTGGAGCCGGTGAGCGTGGGTCTCGCGGTATCATTGCAGCACTGGGGCCAGATGGTAAGCCCTCCCGTATCGTAGTTATCTACACGACGGGGAGTCAGGCAACTATGGATGAACGAAATAGACA

>pEXM4

TCGCGCGTTTCGGTGATGACGGTGAAAACCTCTGACACATGCAGCTCCCGGAGACGGTCACAGCTTGTCTGTAAGCGGATGCCGGGAGCAGACAAGCCCGTCAGGGCGCGTCAGCGGGTGTTGGCGGGTGTCGGGGCTGGCTTAACTATGCGGCATCAGAGCAGATTGTACTGAGAGTGCACCATATGCGGTGTGAAATACCGCACAGATGCGTAAGGAGAAAATACCGCATCAGGCGCCATTCGCCATTCAGGCTGCGCAACTGTTGGGAAGGGCGATCGGTGCGGGCCTCTTCGCTATTACGCCAGCTGGCGAAAGGGGGATGTGCTGCAAGGCGATTAAGTTGGGTAACGCCAGGGTTTTCCCAGTCACGACGTTGTAAAACGACGGCCAGTGAATTCTAGGGATAACAGGGTAATCATTAGGAAGCAGCCCAGTCTCGAGTTAATACGACTCACTATAGGGAGACCACAACGGTTTCCCTCTAGAAATAATTTTGTTTAACTTTAAGAAGGAGATATACATATGGCTAGCATGACTGGTGGACAGCAAATGGGTACTAACCAAGGTAAAGGTGTAGTTGCTGCTGGAGATAAACTGGCGTTGTTCTTGAAGGTATTTGGCGGTGAAGTCCTGACTGCGTTCGCTCGTACCTCCGTGACCACTTCTCGCCACATGGTACGTTCCATCTCCAGCGGTAAATCCGCTCAGTTCCCTGTTCTGGGTCGCACTCAGGCAGCGTATCTGGCTCCGGGCGAGAACCTCGACGATAAACGTAAGGACATCAAACACACCGAGAAGGTAATCACCATTGACGGTCTCCTGACGGCTGACGTTCTGATTTATGATATTGAGGACGCGATGAACCACTACGACGTTCGCTCTGAGTATACCTCTCAGTTGGGTGAATCTCTGGCGATGGCTGCGGATGGTGCGGTTCTGGCTGAGATTGCCGGTCTGTGTAACGTGGAAAGCAAATATAATGAGAACATCGAGGGCTTAGGTACTGCTACCGTAATTGAGACCACTCAGAACAAGGCCGCACTTACCGACCAAGTTGCGCTGGGTAAGGAGATTATTGCGGCTCTGACTAAGGCTCGTGCGGCTCTGACCAAGAACTATGTTCCGGCTGCTGACCGTGTGTTCTACTGTGACCCAGATAGCTACTCTGCGATTCTGGCAGCACTGATGCCGAACGCAGCAAACTACGCTGCTCTGATTGACCCTGAGAAGGGTTCTATCCGCAACGTTATGGGCTTTGAGGTTGTAGAAGTTCCGCACCTCACCGCTGGTGGTGCTGGTACCGCTCGTGAGGGCACTACTGGTCAGAAGCACGTCTTCCCTGCCAATAAAGGTGAGGGTAATGTCAAGGTTGCTAAGGACAACGTTATCGGCCTGTTCATGCACCGCTCTGCGGTAGGTACTGTTAAGCTGCGTGACTTGGCTCTGGAGCGCGCTCGCCGTGCTAACTTCCAAGCGGACCAGATTATCGCTAAGTACGCAATGGGCCACGGTGGTCTTCGCCCAGAAGCTGCTGGTGCAGTGGTTTTCAAAGTGGAGTAACTCGAGAGTAGGTTGAGGCCGTTGAGCTAGGGATAACAGGGTAATTCCCCATCGGTGATGTCGCGGACCGTAACCCCTTGGGGCCTCTAAACGGGTCTTGAGGGGTTTTTTGCTGAAAGGAGGAACTATATGCGCTCATACGATATGAACGTTGAGACTGCCGCTGAGTTATCAGCTGTGAACGACATTCTGGCGTCTATCGGTGAACCTCCGGTATCAACGCTGGAAGGTGACGCTAACGCAGATGCAGCGAACGCTCGGCGTATTCTCAACAAGATTAACCGACAGATTCAATCTCGTGGATGGACGTTCAACATTGAGGAAGGCATAACGCTACTACCTGATGTTTACTCCAACCTGATTGTATACAGTGACGACTATTTATCCCTAATGTCTACTTCCGGTCAATCCATCTACGTTAACCGAGGTGGCTATGTGTATGACCGAACGAGTCAATCAGACCGCTTTGACTCTGGTATTACTGTGAACATTATTCGTCTCCGCGACTACGATGAGATGCCTGAGTGCTTCCGTTACTGGATTGTCACCAAGGCTTCCCGTCAGTTCAACAACCGATTCTTTGGGGCACCGGAAGTAGAGGGTGTACTCCAAGAAGAGGAAGATGAGGCTAGACGTCTCTGCATGGAGTATGAGATGGACTACGGTGGGTACAATATGCTGGATGGAGATGCGTTCACTTCTGGTCTACTGACTCGCTAAAGCTCTAAGGAGGTTATAAAAAGCTAGCATGACCATGATTACCCCGAGCGCGCAGCTGACCCTGACCAAAGGCAACAAAAGCTGGAGCAGCCTGGTGACCGCGGCGAGCGTGCTGGAATTTGCGACCATGATTCAAGGGGTTGCGGGGGAAGTGACGTACGCGGGTGCTTACGACCGTCAGTCGCGCGAGCGCGAAAATTCGAGCGCAGCAAGCCCAGCGACACAGCGTAGCGCCAACGAAGCGAAGGCGGCCGCGCTTCAGCGCGAAATTGAGCGCGCGGGGGGCCGGTGTTTTCGTTTCGTAGGTCATTTTAGCGAAGCTCCGGGTACCTCTGCATTTGGTACCGCAGAACGTCCGGAATTCGAACGTATTCTGAACGAGTGTCGCGCAGGTCGTCTGAACATGATCATCGTCTACGACGTTAGCCGCTTTAGCCGTCTGAAAGTCATGGACGCGATTCCGATTGTTTCTGAACTGCTGGCACTGGGCGTTACCATTGTTAGTACCCAGGAAGGCGTTTTTCGCCAAGGCAACGTTATGGACCTGATCCATCTGATCATGCGTCTGGACGCAAGCCACAAAGAAAGCAGCCTGAAAAGCGCGAAGATCCTGGACACCAAAAATCTGCAACGCGAACTGGGCGGTTACGTTGGCGGTAAAGCACCGTACGGCTTTGAACTGGTTAGCGAAACCAAAGAGATTACCCGCAACGGTCGTATGGTCAACGTCGTCATCAACAAACTGGCGCATAGTACCACCCCGCTGACCGGTCCGTTTGAATTTGAACCGGACGTAATCCGTTGGTGGTGGCGCGAAATTAAGACCCACAAACATCTGCCGTTTAAACCGGGTAGCCAGGCAGCGATTCATCCGGGTTCTATTACCGGCCTGTGTAAACGTATGGATGCAGACGCAGTTCCGACCCGCGGCGAAACCATTGGCAAAAAGACCGCGAGCAGCGCTTGGGATCCGGCTACCGTTATGCGTATTCTGCGCGATCCGCGTATTGCAGGTTTTGCAGCGGAGGTCATCTACAAGAAAAAACCGGACGGTACCCCGACCACCAAAATTGAAGGCTATCGCATTCAACGCGATCCGATTACCCTGCGTCCGGTTGAACTGGATTGCGGTCCGATTATTGAACCGGCAGAGTGGTACGAACTGCAAGCTTGGCTGGACGGTCGCGGTCGCGGTAAAGGTCTGAGTCGCGGTCAAGCTATTCTGTCAGCAATGGACAAACTGTATTGCGAGTGCGGCGCAGTTATGACCAGTAAACGCGGCGAAGAAAGCATCAAAGATAGCTATCGCTGCCGTCGTCGTAAAGTTGTAGATCCGTCTGCTCCGGGTCAACATGAAGGTACCTGTAACGTTAGCATGGCGGCGCTGGATAAATTTGTTGCGGAACGCATCTTCAACAAAATTCGTCACGCGGAAGGCGACGAAGAAACCCTGGCTCTGCTGTGGGAAGCAGCACGTCGTTTTGGTAAACTGACGGAAGCACCGGAAAAATCTGGCGAACGCGCAAATCTGGTTGCAGAACGCGCAGACGCACTGAACGCACTGGAAGAACTGTACGAAGATCGCGCAGCAGGCGCATACGACGGTCCGGTTGGTCGTAAACATTTTCGTAAACAGCAGGCAGCACTGACCCTGCGTCAACAAGGCGCTGAAGAACGTCTGGCAGAACTGGAAGCAGCAGAAGCACCGAAACTGCCGCTGGATCAGTGGTTTCCGGAAGACGCAGATGCAGATCCGACCGGTCCGAAATCTTGGTGGGGTCGCGCATCAGTTGACGATAAACGCGTTTTCGTCGGCCTGTTTGTCGATAAAATCGTTGTTACCAAAAGCACCACCGGTCGTGGTCAAGGTACCCCGATTGAAAAACGCGCAAGCATTACCTGGGCTAAACCGCCGACCGATGACGATGAAGACGACGCACAAGACGGTACCGAAGACGTTGCAGCGTAAGTTATCCACAGAATCAGGGGATAACGCAGGAAAGAACATGTGAGCAAAAGGCCAGCAAAAGGCCAGGAACCGTAAAAAGGCCGCGTTGCTGGCGTTTTTCCATAGGCTCCGCCCCCCTGACGAGCATCACAAAAATCGACGCTCAAGTCAGAGGTGGCGAAACCCGACAGGACTATAAAGATACCAGGCGTTTCCCCCTGGAAGCTCCCTCGTGCGCTCTCCTGTTCCGACCCTGCCGCTTACCGGATACCTGTCCGCCTTTCTCCCTTCGGGAAGCGTGGCGCTTTCTCATAGCTCACGCTGTAGGTATCTCAGTTCGGTGTAGGTCGTTCGCTCCAAGCTGGGCTGTGTGCACGAACCCCCCGTTCAGCCCGACCGCTGCGCCTTATCCGGTAACTATCGTCTTGAGTCCAACCCGGTAAGACACGACTTATCGCCACTGGCAGCAGCCACTGGTAACAGGATTAGCAGAGCGAGGTATGTAGGCGGTGCTACAGAGTTCTTGAAGTGGTGGCCTAACTACGGCTACACTAGAAGAACAGTATTTGGTATCTGCGCTCTGCTGAAGCCAGTTACCTTCGGAAAAAGAGTTGGTAGCTCTTGATCCGGCAAACAAACCACCGCTGGTAGCGGTGGTTTTTTTGTTTGCAAGCAGCAGATTACGCGCAGAAAAAAAGGATCTCAAGAAGATCCTTTGATCTTTTCTACGGGGTCTGACGCTCAGTGGAACGAAAACTCACGTTAAGGGATTTTGGTCATGAGATTATCAAAAAGGATCTTCACCTAGATCCTTTTAAATTAAAAATGAAGTTTTAAATCAATCTAAAGTATATATGAGTAAACTTGGTCTGACAGTTACCAATGCTTAATCAGTGAGGCACCTATCTCAGCGATCTGTCTATTTCGTTCATCCATAGTTGCCTGACTCCCCGTCGTGTAGATAACTACGATACGGGAGGGCTTACCATCTGGCCCCAGTGCTGCAATGATACCGCGACTCCCACGCTCACCGGCTCCAGATTTATCAGCAATAAACCAGCCAGCCGGAAGGGCCGAGCGCAGAAGTGGTCCTGCAACTTTATCCGCCTCCATCCAGTCTATTAATTGTTGCCGGGAAGCTAGAGTAAGTAGTTCGCCAGTTAATAGTTTGCGCAACGTTGTTGCCATTGCTACAGGCATCGTGGTGTCACGCTCGTCGTTTGGTATGGCTTCATTCAGCTCCGGTTCCCAACGATCAAGGCGAGTTACATGATCCCCCATGTTGTGCAAAAAAGCGGTTAGCTCCTTCGGTCCTCCGATCGTTGTCAGAAGTAAGTTGGCCGCAGTGTTATCACTCATGGTTATGGCAGCACTGCATAATTCTCTTACTGTCATGCCATCCGTAAGATGCTTTTCTGTGACTGGTGAGTACTCAACCAAGTCATTCTGAGAATAGTGTATGCGGCGACCGAGTTGCTCTTGCCCGGCGTCAATACGGGATAATACCGCGCCACATAGCAGAACTTTAAAAGTGCTCATCATTGGAAAACGTTCTTCGGGGCGAAAACTCTCAAGGATCTTACCGCTGTTGAGATCCAGTTCGATGTAACCCACTCGTGCACCCAACTGATCTTCAGCATCTTTTACTTTCACCAGCGTTTCTGGGTGAGCAAAAACAGGAAGGCAAAATGCCGCAAAAAAGGGAATAAGGGCGACACGGAAATGTTGAATACTCATACTCTTCCTTTTTCAATATTATTGAAGCATTTATCAGGGTTATTGTCTCATGAGCGGATACATATTTGAATGTATTTAGAAAAATAAACAAATAGGGGTTCCGCGCACATTTCCCCGAAAAGTGCCACCTGACGTCTAAGAAACCATTATTATCATGACATTAACCTATAAAAATAGGCGTATCACGAGGCCCTTTCGTC

>pMCBK

GCTGTTATGGCCGCGTTTGTCTCATTCCACGCCTGACACTCAGTTCCGGGTAGGCAGTTCGCTCCAAGCTGGACTGTATGCACGAACCCCCCGTTCAGTCCGACCGCTGCGCCTTATCCGGTAACTATCGTCTTGAGTCCAACCCGGAAAGACATGCAAAAGCACCACTGGCAGCAGCCACTGGTAATTGATTTAGAGGAGTTAGTCTTGAAGTCATGCGCCGGTTAAGGCTAAACTGAAAGGACAAGTTTTGGTGACTGCGCTCCTCCAAGCCAGTTACCTCGGTTCAAAGAGTTGGTAGCTCAGAGAACCTTCGAAAAACCGCCCTGCAAGGCGGTTTTTTCGTTTTCAGAGCAAGAGATTACGCGCAGACCAAAACGATCTCAAGAAGATCCTTTGATTTTCTACCGAAGAAAGGCCCACCCGTGAAGGTGAGCCAGTGAGTTGATTGTGTAAAACGACGGCCAGTGAATTCCTCGCTGCAGTCCTGAAGCTTCCAGGTCAGAAGCGGTTTTCGGGAGTAGTGCCCCAACTGGGGTAACCTTTGAGTTCTCTCAGTTGGGGGCGTAGGGTCGCCGACATGACACAAGGGGTTAAGCTTGATATCGAATTCGCGTTAATACGACTCACTATAGGGAGACCACAACGGTTTCCCTCTAGACACTCGAGTAACTAGTTAACCCCTTGGGGCCTCTAAACGGGTCTTGAGGGGTTTTTTGCTGAAAGGAGGAACTATATGCGCTCATACGATATGAACGTTGAGACTGCCGCTGAGTTATCAGCTGTGAACGACATTCTGGCGTCTATCGGTGAACCTCCGGTATCAACGCTGGAAGGTGACGCTAACGCAGATGCAGCGAACGCTCGGCGTATTCTCAACAAGATTAACCGACAGATTCAATCTCGTGGATGGACGTTCAACATTGAGGAAGGCATAACGCTACTACCTGATGTTTACTCCAACCTGATTGTATACAGTGACGACTATTTATCCCTAATGTCTACTTCCGGTCAATCCATCTACGTTAACCGAGGTGGCTATGTGTATGACCGAACGAGTCAATCAGACCGCTTTGACTCTGGTATTACTGTGAACATTATTCGTCTCCGCGACTACGATGAGATGCCTGAGTGCTTCCGTTACTGGATTGTCACCAAGGCTTCCCGTCAGTTCAACAACCGATTCTTTGGGGCACCGGAAGTAGAGGGTGTACTCCAAGAAGAGGAAGATGAGGCTAGACGTCTCTGCATGGAGTATGAGATGGACTACGGTGGGTACAATATGCTGGATGGAGATGCGTTCACTTCTGGTCTACTGACTCGCTAAGTTACTCGTGTGCGTCCTTAAGCGGCCGCCTGCAGTCAATACTGACGATGGTCATAGCTGTTTCCTGTCCATAGCAGAAAGTCAAAAGCCTCCGACCGGAGGCTTTTGACTTGATCGGCACGTAAGAGGTTCCAACTTTCACCATAATGAAATAAGATCACTACCGGGCGTATTTTTTGAGTTATCGAGATTTTCAGGAGCTAAGGAAGCTAAAATGAGCCATATTCAACGGGAAACGTCGAGGCCGCGATTAAATTCCAACATGGATGCTGATTTATATGGGTATAAATGGGCTCGCGATAATGTCGGGCAATCAGGTGCGACAATCTATCGCTTGTATGGGAAGCCCGATGCGCCAGAGTTGTTTCTGAAACATGGCAAAGGTAGCGTTGCCAATGATGTTACAGATGAGATGGTCAGACTAAACTGGCTGACGGAATTTATGCCTCTTCCGACCATCAAGCATTTTATCCGTACTCCTGATGATGCATGGTTACTCACCACTGCGATCCCCGGAAAAACAGCATTCCAGGTATTAGAAGAATATCCTGATTCAGGTGAAAATATTGTTGATGCGCTGGCAGTGTTCCTGCGCCGGTTGCATTCGATTCCTGTTTGTAATTGTCCTTTTAACAGCGATCGCGTATTTCGTCTCGCTCAGGCGCAATCACGAATGAATAACGGTTTGGTTGATGCGAGTGATTTTGATGACGAGCGTAATGGCTGGCCTGTTGAACAAGTCTGGAAAGAAATGCATAAACTTTTGCCATTCTCACCGGATTCAGTCGTCACTCATGGTGATTTCTCACTTGATAACCTTATTTTTGACGAGGGGAAATTAATAGGTTGTATTGATGTTGGACGAGTCGGAATCGCAGACCGATACCAGGATCTTGCCATCCTATGGAACTGCCTCGGTGAGTTTTCTCCTTCATTACAGAAACGGCTTTTTCAAAAATATGGTATTGATAATCCTGATATGAATAAATTGCAGTTTCATTTGATGCTCGATGAGTTTTTCTAATGAGGGCCCAAATGTAATCACCTGGCTCACCTTCGGGTGGGCCTTTCTGCGTTGCTGGCGTTTTTCCATAGGCTCCGCCCCCCTGACAAGCATCACGAAATCTGACGCTCAAATCAGTGGTGGCGAAACCCGACAGGACTATAAAGATACCAGGCGTTTCCCCCTGGCGGCTCCCTCGTGCGCTCTCCTGTTCCTGCCTTTCGGTTTACCGGTGTCATTCC

>pMCBK-CE1

GCTGTTATGGCCGCGTTTGTCTCATTCCACGCCTGACACTCAGTTCCGGGTAGGCAGTTCGCTCCAAGCTGGACTGTATGCACGAACCCCCCGTTCAGTCCGACCGCTGCGCCTTATCCGGTAACTATCGTCTTGAGTCCAACCCGGAAAGACATGCAAAAGCACCACTGGCAGCAGCCACTGGTAATTGATTTAGAGGAGTTAGTCTTGAAGTCATGCGCCGGTTAAGGCTAAACTGAAAGGACAAGTTTTGGTGACTGCGCTCCTCCAAGCCAGTTACCTCGGTTCAAAGAGTTGGTAGCTCAGAGAACCTTCGAAAAACCGCCCTGCAAGGCGGTTTTTTCGTTTTCAGAGCAAGAGATTACGCGCAGACCAAAACGATCTCAAGAAGATCCTTTGATTTTCTACCGAAGAAAGGCCCACCCGTGAAGGTGAGCCAGTGAGTTGATTGTGTAAAACGACGGCCAGTGAATTCCTCGCTGCAGTCCTGAAGCTTCCAGGTCAGAAGCGGTTTTCGGGAGTAGTGCCCCAACTGGGGTAACCTTTGAGTTCTCTCAGTTGGGGGCGTAGGGTCGCCGACATGACACAAGGGGTTAAGCTTGATATCAGTTCAAGGTTCTGGGAAATACAGACCGCCACAGTATCAAAAAAAATCTTATAGGGGCTCTTTTATTTGACAGTGGAGAGACAGCGGAAGCGACTCGCCTCAAACGGACAGCTCGTAGAAGGTATACACGTCGGAAGAATCGTATTTGTTATCTACAGGAGATTTTTTCAAATGAGATGGCGAAAGTAGATGATAGTTTCTTTCATCGACTTGAAGAGTCTTTTTTGGTGGAAGAAGACAAGAAGCATGAACGTCATCCTATTTTTGGAAATATAGTAGATGAAGTTGCTTATCATGAGAAATATCCAACTATCTATCATCTGCGAAAAAAATTGGTAGATTCTACTGATAAAGCGGATTTGCGCTTAATCTATTTGGCCTTAGCGCATATGATTAAGTTTCGTGGTCATTTTTTGATTGAGGGAGATTTAAATCCTGATAATAGTGATGTGGACAAACTATTTATCCAGTTGGTACAAACCTACAATCAATTATTTGAAGAAAACCCTATTAACGCAAGTGGAGTAGATGCTAAAGCGATTCTTTCTGCACGATTGAGTAAATCAAGACGATTAGAAAATCTCATTGCTCAGCTCCCCGGTGAGAAGAAAAATGGCTTATTTGGGAATCTCATTGCTTTGTCATTGGGTTTGACCCCTAATTTTAAATCAAATTTTGATTTGGCAGAAGATGCTAAATTACAGCTTTCAAAAGATACTTACGATGATGATTTAGATAATTTATTGGCGCAAATTGGAGATCAATATGCTGATTTGTTTTTGGCAGCTAAGAATTTATCAGATGCTATTTTACTTTCAGATATCCTAAGAGTAAATACTGAAATAACTAAGGCTCCCCTATCAGCGATATCGAATTCGCGTTAATACGACTCACTATAGGGAGACCACAACGGTTTCCCTCTAGACACTCGAGTAACTAGTTAACCCCTTGGGGCCTCTAAACGGGTCTTGAGGGGTTTTTTGCTGAAAGGAGGAACTATATGCGCTCATACGATATGAACGTTGAGACTGCCGCTGAGTTATCAGCTGTGAACGACATTCTGGCGTCTATCGGTGAACCTCCGGTATCAACGCTGGAAGGTGACGCTAACGCAGATGCAGCGAACGCTCGGCGTATTCTCAACAAGATTAACCGACAGATTCAATCTCGTGGATGGACGTTCAACATTGAGGAAGGCATAACGCTACTACCTGATGTTTACTCCAACCTGATTGTATACAGTGACGACTATTTATCCCTAATGTCTACTTCCGGTCAATCCATCTACGTTAACCGAGGTGGCTATGTGTATGACCGAACGAGTCAATCAGACCGCTTTGACTCTGGTATTACTGTGAACATTATTCGTCTCCGCGACTACGATGAGATGCCTGAGTGCTTCCGTTACTGGATTGTCACCAAGGCTTCCCGTCAGTTCAACAACCGATTCTTTGGGGCACCGGAAGTAGAGGGTGTACTCCAAGAAGAGGAAGATGAGGCTAGACGTCTCTGCATGGAGTATGAGATGGACTACGGTGGGTACAATATGCTGGATGGAGATGCGTTCACTTCTGGTCTACTGACTCGCTAAGTTACTCGTGTGCGTCCTTAAGCGGCCGCCTGCAGTCAATACTGACGATGGTCATAGCTGTTTCCTGTCCATAGCAGAAAGTCAAAAGCCTCCGACCGGAGGCTTTTGACTTGATCGGCACGTAAGAGGTTCCAACTTTCACCATAATGAAATAAGATCACTACCGGGCGTATTTTTTGAGTTATCGAGATTTTCAGGAGCTAAGGAAGCTAAAATGAGCCATATTCAACGGGAAACGTCGAGGCCGCGATTAAATTCCAACATGGATGCTGATTTATATGGGTATAAATGGGCTCGCGATAATGTCGGGCAATCAGGTGCGACAATCTATCGCTTGTATGGGAAGCCCGATGCGCCAGAGTTGTTTCTGAAACATGGCAAAGGTAGCGTTGCCAATGATGTTACAGATGAGATGGTCAGACTAAACTGGCTGACGGAATTTATGCCTCTTCCGACCATCAAGCATTTTATCCGTACTCCTGATGATGCATGGTTACTCACCACTGCGATCCCCGGAAAAACAGCATTCCAGGTATTAGAAGAATATCCTGATTCAGGTGAAAATATTGTTGATGCGCTGGCAGTGTTCCTGCGCCGGTTGCATTCGATTCCTGTTTGTAATTGTCCTTTTAACAGCGATCGCGTATTTCGTCTCGCTCAGGCGCAATCACGAATGAATAACGGTTTGGTTGATGCGAGTGATTTTGATGACGAGCGTAATGGCTGGCCTGTTGAACAAGTCTGGAAAGAAATGCATAAACTTTTGCCATTCTCACCGGATTCAGTCGTCACTCATGGTGATTTCTCACTTGATAACCTTATTTTTGACGAGGGGAAATTAATAGGTTGTATTGATGTTGGACGAGTCGGAATCGCAGACCGATACCAGGATCTTGCCATCCTATGGAACTGCCTCGGTGAGTTTTCTCCTTCATTACAGAAACGGCTTTTTCAAAAATATGGTATTGATAATCCTGATATGAATAAATTGCAGTTTCATTTGATGCTCGATGAGTTTTTCTAATGAGGGCCCAAATGTAATCACCTGGCTCACCTTCGGGTGGGCCTTTCTGCGTTGCTGGCGTTTTTCCATAGGCTCCGCCCCCCTGACAAGCATCACGAAATCTGACGCTCAAATCAGTGGTGGCGAAACCCGACAGGACTATAAAGATACCAGGCGTTTCCCCCTGGCGGCTCCCTCGTGCGCTCTCCTGTTCCTGCCTTTCGGTTTACCGGTGTCATTCC

>pMCBK-CE2

GCTGTTATGGCCGCGTTTGTCTCATTCCACGCCTGACACTCAGTTCCGGGTAGGCAGTTCGCTCCAAGCTGGACTGTATGCACGAACCCCCCGTTCAGTCCGACCGCTGCGCCTTATCCGGTAACTATCGTCTTGAGTCCAACCCGGAAAGACATGCAAAAGCACCACTGGCAGCAGCCACTGGTAATTGATTTAGAGGAGTTAGTCTTGAAGTCATGCGCCGGTTAAGGCTAAACTGAAAGGACAAGTTTTGGTGACTGCGCTCCTCCAAGCCAGTTACCTCGGTTCAAAGAGTTGGTAGCTCAGAGAACCTTCGAAAAACCGCCCTGCAAGGCGGTTTTTTCGTTTTCAGAGCAAGAGATTACGCGCAGACCAAAACGATCTCAAGAAGATCCTTTGATTTTCTACCGAAGAAAGGCCCACCCGTGAAGGTGAGCCAGTGAGTTGATTGTGTAAAACGACGGCCAGTGAATTCCTCGCTGCAGTCCTGAAGCTTCCAGGTCAGAAGCGGTTTTCGGGAGTAGTGCCCCAACTGGGGTAACCTTTGAGTTCTCTCAGTTGGGGGCGTAGGGTCGCCGACATGACACAAGGGGTTAAGCTTGATATCAGTTCAAGGTTCTGGGAAATACAGACCGCCACAGTATCAAAAAAAATCTTATAGGGGCTCTTTTATTTGACAGTGGAGAGACAGCGGAAGCGACTCGCCTCAAACGGACAGCTCGTAGAAGGTATACACGTCGGAAGAATCGTATTTGTTATCTACAGGAGATTTTTTCAAATGAGATGGCGAAAGTAGATGATAGTTTCTTTCATCGACTTGAAGAGTCTTTTTTGGTGGAAGAAGACAAGAAGCATGAACGTCATCCTATTTTTGGAAATATAGTAGATGAAGTTGCTTATCATGAGAAATATCCAACTATCTATCATCTGCGAAAAAAATTGGTAGATTCTACTGATAAAGCGGATTTGCGCTTAATCTATTTGGCCTTAGCGCATATGATTAAGTTTCGTGGTCATTTTTTGATTGAGGGAGATTTAAATCCTGATAATAGTGATGTGGACAAACTATTTATCCAGTTGGTACAAACCTACAATCAATTATTTGAAGAAAACCCTATTAACGCAAGTGGAGTAGATGCTAAAGCGATTCTTTCTGCACGATTGAGTAAATCAAGACGATTAGAAAATCTCATTGCTCAGCTCCCCGGTGAGAAGAAAAATGGCTTATTTGGGAATCTCATTGCTTTGTCATTGGGTTTGACCCCTAATTTTAAATCAAATTTTGATTTGGCAGAAGATGCTAAATTACAGCTTTCAAAAGATACTTACGATGATGATTTAGATAATTTATTGGCGCAAATTGGAGATCAATATGCTGATTTGTTTTTGGCAGCTAAGAATTTATCAGATGCTATTTTACTTTCAGATATCCTAAGAGTAAATACTGAAATAACTAAGGCTCCCCTATCAGCTTCAATGATTAAACGCTACGATGAACATCATCAAGACTTGACTCTTTTAAAAGCTTTAGTTCGACAACAACTTCCAGAAAAGTATAAAGAAATCTTTTTTGATCAATCAAAAAACGGATATGCAGGTTATATTGATGGGGGAGCTAGCCAAGAAGAATTTTATAAATTTATCAAACCAATTTTAGAAAAAATGGATGGTACTGAGGAATTATTGGTGAAACTAAATCGTGAAGATTTGCTGCGCAAGCAACGGACCTTTGACAACGGCTCTATTCCCCATCAAATTCACTTGGGTGAGCTGCATGCTATTTTGAGAAGACAAGAAGACTTTTATCCATTTTTAAAAGACAATCGTGAGAAGATTGAAAAAATCTTGACTTTTCGAATTCCTTATTATGTTGGTCCATTGGCGCGTGGCAATAGTCGTTTTGGATATCGAATTCGCGTTAATACGACTCACTATAGGGAGACCACAACGGTTTCCCTCTAGACACTCGAGTAACTAGTTAACCCCTTGGGGCCTCTAAACGGGTCTTGAGGGGTTTTTTGCTGAAAGGAGGAACTATATGCGCTCATACGATATGAACGTTGAGACTGCCGCTGAGTTATCAGCTGTGAACGACATTCTGGCGTCTATCGGTGAACCTCCGGTATCAACGCTGGAAGGTGACGCTAACGCAGATGCAGCGAACGCTCGGCGTATTCTCAACAAGATTAACCGACAGATTCAATCTCGTGGATGGACGTTCAACATTGAGGAAGGCATAACGCTACTACCTGATGTTTACTCCAACCTGATTGTATACAGTGACGACTATTTATCCCTAATGTCTACTTCCGGTCAATCCATCTACGTTAACCGAGGTGGCTATGTGTATGACCGAACGAGTCAATCAGACCGCTTTGACTCTGGTATTACTGTGAACATTATTCGTCTCCGCGACTACGATGAGATGCCTGAGTGCTTCCGTTACTGGATTGTCACCAAGGCTTCCCGTCAGTTCAACAACCGATTCTTTGGGGCACCGGAAGTAGAGGGTGTACTCCAAGAAGAGGAAGATGAGGCTAGACGTCTCTGCATGGAGTATGAGATGGACTACGGTGGGTACAATATGCTGGATGGAGATGCGTTCACTTCTGGTCTACTGACTCGCTAAGTTACTCGTGTGCGTCCTTAAGCGGCCGCCTGCAGTCAATACTGACGATGGTCATAGCTGTTTCCTGTCCATAGCAGAAAGTCAAAAGCCTCCGACCGGAGGCTTTTGACTTGATCGGCACGTAAGAGGTTCCAACTTTCACCATAATGAAATAAGATCACTACCGGGCGTATTTTTTGAGTTATCGAGATTTTCAGGAGCTAAGGAAGCTAAAATGAGCCATATTCAACGGGAAACGTCGAGGCCGCGATTAAATTCCAACATGGATGCTGATTTATATGGGTATAAATGGGCTCGCGATAATGTCGGGCAATCAGGTGCGACAATCTATCGCTTGTATGGGAAGCCCGATGCGCCAGAGTTGTTTCTGAAACATGGCAAAGGTAGCGTTGCCAATGATGTTACAGATGAGATGGTCAGACTAAACTGGCTGACGGAATTTATGCCTCTTCCGACCATCAAGCATTTTATCCGTACTCCTGATGATGCATGGTTACTCACCACTGCGATCCCCGGAAAAACAGCATTCCAGGTATTAGAAGAATATCCTGATTCAGGTGAAAATATTGTTGATGCGCTGGCAGTGTTCCTGCGCCGGTTGCATTCGATTCCTGTTTGTAATTGTCCTTTTAACAGCGATCGCGTATTTCGTCTCGCTCAGGCGCAATCACGAATGAATAACGGTTTGGTTGATGCGAGTGATTTTGATGACGAGCGTAATGGCTGGCCTGTTGAACAAGTCTGGAAAGAAATGCATAAACTTTTGCCATTCTCACCGGATTCAGTCGTCACTCATGGTGATTTCTCACTTGATAACCTTATTTTTGACGAGGGGAAATTAATAGGTTGTATTGATGTTGGACGAGTCGGAATCGCAGACCGATACCAGGATCTTGCCATCCTATGGAACTGCCTCGGTGAGTTTTCTCCTTCATTACAGAAACGGCTTTTTCAAAAATATGGTATTGATAATCCTGATATGAATAAATTGCAGTTTCATTTGATGCTCGATGAGTTTTTCTAATGAGGGCCCAAATGTAATCACCTGGCTCACCTTCGGGTGGGCCTTTCTGCGTTGCTGGCGTTTTTCCATAGGCTCCGCCCCCCTGACAAGCATCACGAAATCTGACGCTCAAATCAGTGGTGGCGAAACCCGACAGGACTATAAAGATACCAGGCGTTTCCCCCTGGCGGCTCCCTCGTGCGCTCTCCTGTTCCTGCCTTTCGGTTTACCGGTGTCATTCC

>pMCBK-CE3

GCTGTTATGGCCGCGTTTGTCTCATTCCACGCCTGACACTCAGTTCCGGGTAGGCAGTTCGCTCCAAGCTGGACTGTATGCACGAACCCCCCGTTCAGTCCGACCGCTGCGCCTTATCCGGTAACTATCGTCTTGAGTCCAACCCGGAAAGACATGCAAAAGCACCACTGGCAGCAGCCACTGGTAATTGATTTAGAGGAGTTAGTCTTGAAGTCATGCGCCGGTTAAGGCTAAACTGAAAGGACAAGTTTTGGTGACTGCGCTCCTCCAAGCCAGTTACCTCGGTTCAAAGAGTTGGTAGCTCAGAGAACCTTCGAAAAACCGCCCTGCAAGGCGGTTTTTTCGTTTTCAGAGCAAGAGATTACGCGCAGACCAAAACGATCTCAAGAAGATCCTTTGATTTTCTACCGAAGAAAGGCCCACCCGTGAAGGTGAGCCAGTGAGTTGATTGTGTAAAACGACGGCCAGTGAATTCCTCGCTGCAGTCCTGAAGCTTCCAGGTCAGAAGCGGTTTTCGGGAGTAGTGCCCCAACTGGGGTAACCTTTGAGTTCTCTCAGTTGGGGGCGTAGGGTCGCCGACATGACACAAGGGGTTAAGCTTGATATCAAACCCTATTAACGCAAGTGGAGTAGATGCTAAAGCGATTCTTTCTGCACGATTGAGTAAATCAAGACGATTAGAAAATCTCATTGCTCAGCTCCCCGGTGAGAAGAAAAATGGCTTATTTGGGAATCTCATTGCTTTGTCATTGGGTTTGACCCCTAATTTTAAATCAAATTTTGATTTGGCAGAAGATGCTAAATTACAGCTTTCAAAAGATACTTACGATGATGATTTAGATAATTTATTGGCGCAAATTGGAGATCAATATGCTGATTTGTTTTTGGCAGCTAAGAATTTATCAGATGCTATTTTACTTTCAGATATCCTAAGAGTAAATACTGAAATAACTAAGGCTCCCCTATCAGCTTCAATGATTAAACGCTACGATGAACATCATCAAGACTTGACTCTTTTAAAAGCTTTAGTTCGACAACAACTTCCAGAAAAGTATAAAGAAATCTTTTTTGATCAATCAAAAAACGGATATGCAGGTTATATTGATGGGGGAGCTAGCCAAGAAGAATTTTATAAATTTATCAAACCAATTTTAGAAAAAATGGATGGTACTGAGGAATTATTGGTGAAACTAAATCGTGAAGATTTGCTGCGCAAGCAACGGACCTTTGACAACGGCTCTATTCCCCATCAAATTCACTTGGGTGAGCTGCATGCTATTTTGAGAAGACAAGAAGACTTTTATCCATTTTTAAAAGACAATCGTGAGAAGATTGAAAAAATCTTGACTTTTCGAATTCCTTATTATGTTGGTCCATTGGCGCGTGGCAATAGTCGTTTTGCATGGATGACTCGGAAGTCTGAAGAAACAATTACCCCATGGAATTTTGAAGAAGTTGTCGATAAAGGTGCTTCAGCTCAATCATTTATTGAACGCATGACAGCGTTTGATAAAAATCTTCCAAATGAAAAAGTACTACCAAAACATAGTTTGCTTTATGAGTATTTTACGGTTTATAACGAATTGACAAAGGTCAAATATGTTACTGAAGGAATGCGAAAACCAGCATTTCTTTCAGGTGAACAGAAGAAAGCCATTGTTGATTTACTCTTCAAAACAAATCGAAAAGTAACCGTTAAGCAATTAAAAGAAGATTATTTCAAAAAAATAGAATGTTTTGATAGTGTTGAAATTTCAGGAGTTGAAGATAGATTTAATGCTTCATTAGGTACCTACCATGATTTGCTAAAAATTATTAAAGATAAAGATTTTTTGGATAATGAAGAAAATGAAGATATCTTAGAGGATATTGTTTTAACATTGACCTTATTTGAAGATAGGGAGATGATTGAGGAAAGACTTAAAACATATGCTCACCTCTTTGATGATAAGGTGATGAAACAGCTTAAACGTCGCCGTTATACTGGTTGGGGAGCGTTGTCTCGAAAATTGATTAATGGTATTAGGGATAAGCAATCTGGCAAAACAATATTAGATTTTTTGAAATCAGATGGTTTTGCCAATCGCAATTTTATGGCGCTGATCCATGATGATAGTTTGACATTTAAAGAAGACATTCAAAAAGCACAAGTGTCCGGACAAGGCGATAGTTTACATGAACATATTGCAAATTTAGCTGGTAGCCCTGCTATTAAAAAAGGTATTTTACAGACTGTAAAAGTTGTTGATGAATTGGTCAAAGTAATGGGGCGGCATAAGCCAGAAAATATCGTTATTGAAATGGCACGTGAAAATCAGACAACTCAAAAGGGCCAGAAAAATTCGCGAGAGCGTATGAAACGAATCGAAGAAGGTATCAAAGAATTAGGAAGTCAGATTCTTAAAGAGCATCCTGTTGAAAATACTCAATTGCAAAATGAAAAGCTCTATCTCTATTATCTCCAAAATGGAAGAGACATGTATGTGGACCAAGAATTAGATATTAATCGTTTAAGTGATTATGATGTCGATCACATTGTTCCACAAAGTTTCCTTAAAGACGATTCAATAGACAATAAGGTCTTAACGCGTTCTGATAAAAATCGTGGTAAATCGGATAACGTTCCAAGTGAAGAAGTAGTCAAAAAGATGAAAAACTATTGGAGACAACTTCTAAACGCCAAGTTAATCACTCAACGTAAGTTTGATAATTTAACGAAAGCTGAACGTGGAGGTTTGAGTGAACTTGATAAAGCTGGTTTTATCAAACGCCAATTGGTTGAAACTCGCGCGATCACTAAGCATGTGGCACAAATTTTGGATAGTCGCATGAATACTAAATACGATGAAAATGATAAACTTATTCGAGAGGTTAAAGTGATTACCTTAAAATCTAAATTAGTTTCTGACTTCCGAAAAGATTTCCAATTCTATAAAGTACGTGAGATTAACAATTACCATCATGCCCATGATGCGTATCTAAATGCCGTCGTTGGAACTGCTTTGAGATATCGAATTCGCGTTAATACGACTCACTATAGGGAGACCACAACGGTTTCCCTCTAGACACTCGAGTAACTAGTTAACCCCTTGGGGCCTCTAAACGGGTCTTGAGGGGTTTTTTGCTGAAAGGAGGAACTATATGCGCTCATACGATATGAACGTTGAGACTGCCGCTGAGTTATCAGCTGTGAACGACATTCTGGCGTCTATCGGTGAACCTCCGGTATCAACGCTGGAAGGTGACGCTAACGCAGATGCAGCGAACGCTCGGCGTATTCTCAACAAGATTAACCGACAGATTCAATCTCGTGGATGGACGTTCAACATTGAGGAAGGCATAACGCTACTACCTGATGTTTACTCCAACCTGATTGTATACAGTGACGACTATTTATCCCTAATGTCTACTTCCGGTCAATCCATCTACGTTAACCGAGGTGGCTATGTGTATGACCGAACGAGTCAATCAGACCGCTTTGACTCTGGTATTACTGTGAACATTATTCGTCTCCGCGACTACGATGAGATGCCTGAGTGCTTCCGTTACTGGATTGTCACCAAGGCTTCCCGTCAGTTCAACAACCGATTCTTTGGGGCACCGGAAGTAGAGGGTGTACTCCAAGAAGAGGAAGATGAGGCTAGACGTCTCTGCATGGAGTATGAGATGGACTACGGTGGGTACAATATGCTGGATGGAGATGCGTTCACTTCTGGTCTACTGACTCGCTAAGTTACTCGTGTGCGTCCTTAAGCGGCCGCCTGCAGTCAATACTGACGATGGTCATAGCTGTTTCCTGTCCATAGCAGAAAGTCAAAAGCCTCCGACCGGAGGCTTTTGACTTGATCGGCACGTAAGAGGTTCCAACTTTCACCATAATGAAATAAGATCACTACCGGGCGTATTTTTTGAGTTATCGAGATTTTCAGGAGCTAAGGAAGCTAAAATGAGCCATATTCAACGGGAAACGTCGAGGCCGCGATTAAATTCCAACATGGATGCTGATTTATATGGGTATAAATGGGCTCGCGATAATGTCGGGCAATCAGGTGCGACAATCTATCGCTTGTATGGGAAGCCCGATGCGCCAGAGTTGTTTCTGAAACATGGCAAAGGTAGCGTTGCCAATGATGTTACAGATGAGATGGTCAGACTAAACTGGCTGACGGAATTTATGCCTCTTCCGACCATCAAGCATTTTATCCGTACTCCTGATGATGCATGGTTACTCACCACTGCGATCCCCGGAAAAACAGCATTCCAGGTATTAGAAGAATATCCTGATTCAGGTGAAAATATTGTTGATGCGCTGGCAGTGTTCCTGCGCCGGTTGCATTCGATTCCTGTTTGTAATTGTCCTTTTAACAGCGATCGCGTATTTCGTCTCGCTCAGGCGCAATCACGAATGAATAACGGTTTGGTTGATGCGAGTGATTTTGATGACGAGCGTAATGGCTGGCCTGTTGAACAAGTCTGGAAAGAAATGCATAAACTTTTGCCATTCTCACCGGATTCAGTCGTCACTCATGGTGATTTCTCACTTGATAACCTTATTTTTGACGAGGGGAAATTAATAGGTTGTATTGATGTTGGACGAGTCGGAATCGCAGACCGATACCAGGATCTTGCCATCCTATGGAACTGCCTCGGTGAGTTTTCTCCTTCATTACAGAAACGGCTTTTTCAAAAATATGGTATTGATAATCCTGATATGAATAAATTGCAGTTTCATTTGATGCTCGATGAGTTTTTCTAATGAGGGCCCAAATGTAATCACCTGGCTCACCTTCGGGTGGGCCTTTCTGCGTTGCTGGCGTTTTTCCATAGGCTCCGCCCCCCTGACAAGCATCACGAAATCTGACGCTCAAATCAGTGGTGGCGAAACCCGACAGGACTATAAAGATACCAGGCGTTTCCCCCTGGCGGCTCCCTCGTGCGCTCTCCTGTTCCTGCCTTTCGGTTTACCGGTGTCATTCC

>pMCBK-CE4

GCTGTTATGGCCGCGTTTGTCTCATTCCACGCCTGACACTCAGTTCCGGGTAGGCAGTTCGCTCCAAGCTGGACTGTATGCACGAACCCCCCGTTCAGTCCGACCGCTGCGCCTTATCCGGTAACTATCGTCTTGAGTCCAACCCGGAAAGACATGCAAAAGCACCACTGGCAGCAGCCACTGGTAATTGATTTAGAGGAGTTAGTCTTGAAGTCATGCGCCGGTTAAGGCTAAACTGAAAGGACAAGTTTTGGTGACTGCGCTCCTCCAAGCCAGTTACCTCGGTTCAAAGAGTTGGTAGCTCAGAGAACCTTCGAAAAACCGCCCTGCAAGGCGGTTTTTTCGTTTTCAGAGCAAGAGATTACGCGCAGACCAAAACGATCTCAAGAAGATCCTTTGATTTTCTACCGAAGAAAGGCCCACCCGTGAAGGTGAGCCAGTGAGTTGATTGTGTAAAACGACGGCCAGTGAATTCCTCGCTGCAGTCCTGAAGCTTCCAGGTCAGAAGCGGTTTTCGGGAGTAGTGCCCCAACTGGGGTAACCTTTGAGTTCTCTCAGTTGGGGGCGTAGGGTCGCCGACATGACACAAGGGGTTAAGCTTGATATCCAAATGAGATGGCGAAAGTAGATGATAGTTTCTTTCATCGACTTGAAGAGTCTTTTTTGGTGGAAGAAGACAAGAAGCATGAACGTCATCCTATTTTTGGAAATATAGTAGATGAAGTTGCTTATCATGAGAAATATCCAACTATCTATCATCTGCGAAAAAAATTGGTAGATTCTACTGATAAAGCGGATTTGCGCTTAATCTATTTGGCCTTAGCGCATATGATTAAGTTTCGTGGTCATTTTTTGATTGAGGGAGATTTAAATCCTGATAATAGTGATGTGGACAAACTATTTATCCAGTTGGTACAAACCTACAATCAATTATTTGAAGAAAACCCTATTAACGCAAGTGGAGTAGATGCTAAAGCGATTCTTTCTGCACGATTGAGTAAATCAAGACGATTAGAAAATCTCATTGCTCAGCTCCCCGGTGAGAAGAAAAATGGCTTATTTGGGAATCTCATTGCTTTGTCATTGGGTTTGACCCCTAATTTTAAATCAAATTTTGATTTGGCAGAAGATGCTAAATTACAGCTTTCAAAAGATACTTACGATGATGATTTAGATAATTTATTGGCGCAAATTGGAGATCAATATGCTGATTTGTTTTTGGCAGCTAAGAATTTATCAGATGCTATTTTACTTTCAGATATCCTAAGAGTAAATACTGAAATAACTAAGGCTCCCCTATCAGCTTCAATGATTAAACGCTACGATGAACATCATCAAGACTTGACTCTTTTAAAAGCTTTAGTTCGACAACAACTTCCAGAAAAGTATAAAGAAATCTTTTTTGATCAATCAAAAAACGGATATGCAGGTTATATTGATGGGGGAGCTAGCCAAGAAGAATTTTATAAATTTATCAAACCAATTTTAGAAAAAATGGATGGTACTGAGGAATTATTGGTGAAACTAAATCGTGAAGATTTGCTGCGCAAGCAACGGACCTTTGACAACGGCTCTATTCCCCATCAAATTCACTTGGGTGAGCTGCATGCTATTTTGAGAAGACAAGAAGACTTTTATCCATTTTTAAAAGACAATCGTGAGAAGATTGAAAAAATCTTGACTTTTCGAATTCCTTATTATGTTGGTCCATTGGCGCGTGGCAATAGTCGTTTTGCATGGATGACTCGGAAGTCTGAAGAAACAATTACCCCATGGAATTTTGAAGAAGTTGTCGATAAAGGTGCTTCAGCTCAATCATTTATTGAACGCATGACAGCGTTTGATAAAAATCTTCCAAATGAAAAAGTACTACCAAAACATAGTTTGCTTTATGAGTATTTTACGGTTTATAACGAATTGACAAAGGTCAAATATGTTACTGAAGGAATGCGAAAACCAGCATTTCTTTCAGGTGAACAGAAGAAAGCCATTGTTGATTTACTCTTCAAAACAAATCGAAAAGTAACCGTTAAGCAATTAAAAGAAGATTATTTCAAAAAAATAGAATGTTTTGATAGTGTTGAAATTTCAGGAGTTGAAGATAGATTTAATGCTTCATTAGGTACCTACCATGATTTGCTAAAAATTATTAAAGATAAAGATTTTTTGGATAATGAAGAAAATGAAGATATCTTAGAGGATATTGTTTTAACATTGACCTTATTTGAAGATAGGGAGATGATTGAGGAAAGACTTAAAACATATGCTCACCTCTTTGATGATAAGGTGATGAAACAGCTTAAACGTCGCCGTTATACTGGTTGGGGAGCGTTGTCTCGAAAATTGATTAATGGTATTAGGGATAAGCAATCTGGCAAAACAATATTAGATTTTTTGAAATCAGATGGTTTTGCCAATCGCAATTTTATGGCGCTGATCCATGATGATAGTTTGACATTTAAAGAAGACATTCAAAAAGCACAAGTGTCCGGACAAGGCGATAGTTTACATGAACATATTGCAAATTTAGCTGGTAGCCCTGCTATTAAAAAAGGTATTTTACAGACTGTAAAAGTTGTTGATGAATTGGTCAAAGTAATGGGGCGGCATAAGCCAGAAAATATCGTTATTGAAATGGCACGTGAAAATCAGACAACTCAAAAGGGCCAGAAAAATTCGCGAGAGCGTATGAAACGAATCGAAGAAGGTATCAAAGAATTAGGAAGTCAGATTCTTAAAGAGCATCCTGTTGAAAATACTCAATTGCAAAATGAAAAGCTCTATCTCTATTATCTCCAAAATGGAAGAGACATGTATGTGGACCAAGAATTAGATATTAATCGTTTAAGTGATTATGATGTCGATCACATTGTTCCACAAAGTTTCCTTAAAGACGATTCAATAGACAATAAGGTCTTAACGCGTTCTGATAAAAATCGTGGTAAATCGGATAACGTTCCAAGTGAAGAAGTAGTCAAAAAGATGAAAAACTATTGGAGACAACTTCTAAACGCCAAGTTAATCACTCAACGTAAGTTTGATAATTTAACGAAAGCTGAACGTGGAGGTTTGAGTGAACTTGATAAAGCTGGTTTTATCAAACGCCAATTGGTTGAAACTCGCGCGATCACTAAGCATGTGGCACAAATTTTGGATAGTCGCATGAATACTAAATACGATGAAAATGATAAACTTATTCGAGAGGTTAAAGTGATTACCTTAAAATCTAAATTAGTTTCTGACTTCCGAAAAGATTTCCAATTCTATAAAGTACGTGAGATTAACAATTACCATCATGCCCATGATGCGTATCTAAATGCCGTCGTTGGAACTGCTTTGATTAAGAAATATCCAAAACTTGAATCGGAGTTTGTCTATGGTGATTATAAAGTTTATGATGTTCGTAAAATGATTGCTAAGTCTGAGCAAGAAATAGGCAAAGCAACCGCAAAATATTTCTTTTACTCTAATATCATGAACTTCTTCAAAACAGAAATTACACTTGCAAATGGAGAGATTCGCAAACGCCCTCTAATCGAAACTAATGGGGAAACTGGAGAAATTGTCTGGGATAAAGGGCGAGATTTTGCCACAGTGCGCAAAGTATTGTCCATGCCCCAAGTCAATATTGTCAAGAAAACAGAAGTACAGACAGGCGGATTCTCGATATCGAATTCGCGTTAATACGACTCACTATAGGGAGACCACAACGGTTTCCCTCTAGACACTCGAGTAACTAGTTAACCCCTTGGGGCCTCTAAACGGGTCTTGAGGGGTTTTTTGCTGAAAGGAGGAACTATATGCGCTCATACGATATGAACGTTGAGACTGCCGCTGAGTTATCAGCTGTGAACGACATTCTGGCGTCTATCGGTGAACCTCCGGTATCAACGCTGGAAGGTGACGCTAACGCAGATGCAGCGAACGCTCGGCGTATTCTCAACAAGATTAACCGACAGATTCAATCTCGTGGATGGACGTTCAACATTGAGGAAGGCATAACGCTACTACCTGATGTTTACTCCAACCTGATTGTATACAGTGACGACTATTTATCCCTAATGTCTACTTCCGGTCAATCCATCTACGTTAACCGAGGTGGCTATGTGTATGACCGAACGAGTCAATCAGACCGCTTTGACTCTGGTATTACTGTGAACATTATTCGTCTCCGCGACTACGATGAGATGCCTGAGTGCTTCCGTTACTGGATTGTCACCAAGGCTTCCCGTCAGTTCAACAACCGATTCTTTGGGGCACCGGAAGTAGAGGGTGTACTCCAAGAAGAGGAAGATGAGGCTAGACGTCTCTGCATGGAGTATGAGATGGACTACGGTGGGTACAATATGCTGGATGGAGATGCGTTCACTTCTGGTCTACTGACTCGCTAAGTTACTCGTGTGCGTCCTTAAGCGGCCGCCTGCAGTCAATACTGACGATGGTCATAGCTGTTTCCTGTCCATAGCAGAAAGTCAAAAGCCTCCGACCGGAGGCTTTTGACTTGATCGGCACGTAAGAGGTTCCAACTTTCACCATAATGAAATAAGATCACTACCGGGCGTATTTTTTGAGTTATCGAGATTTTCAGGAGCTAAGGAAGCTAAAATGAGCCATATTCAACGGGAAACGTCGAGGCCGCGATTAAATTCCAACATGGATGCTGATTTATATGGGTATAAATGGGCTCGCGATAATGTCGGGCAATCAGGTGCGACAATCTATCGCTTGTATGGGAAGCCCGATGCGCCAGAGTTGTTTCTGAAACATGGCAAAGGTAGCGTTGCCAATGATGTTACAGATGAGATGGTCAGACTAAACTGGCTGACGGAATTTATGCCTCTTCCGACCATCAAGCATTTTATCCGTACTCCTGATGATGCATGGTTACTCACCACTGCGATCCCCGGAAAAACAGCATTCCAGGTATTAGAAGAATATCCTGATTCAGGTGAAAATATTGTTGATGCGCTGGCAGTGTTCCTGCGCCGGTTGCATTCGATTCCTGTTTGTAATTGTCCTTTTAACAGCGATCGCGTATTTCGTCTCGCTCAGGCGCAATCACGAATGAATAACGGTTTGGTTGATGCGAGTGATTTTGATGACGAGCGTAATGGCTGGCCTGTTGAACAAGTCTGGAAAGAAATGCATAAACTTTTGCCATTCTCACCGGATTCAGTCGTCACTCATGGTGATTTCTCACTTGATAACCTTATTTTTGACGAGGGGAAATTAATAGGTTGTATTGATGTTGGACGAGTCGGAATCGCAGACCGATACCAGGATCTTGCCATCCTATGGAACTGCCTCGGTGAGTTTTCTCCTTCATTACAGAAACGGCTTTTTCAAAAATATGGTATTGATAATCCTGATATGAATAAATTGCAGTTTCATTTGATGCTCGATGAGTTTTTCTAATGAGGGCCCAAATGTAATCACCTGGCTCACCTTCGGGTGGGCCTTTCTGCGTTGCTGGCGTTTTTCCATAGGCTCCGCCCCCCTGACAAGCATCACGAAATCTGACGCTCAAATCAGTGGTGGCGAAACCCGACAGGACTATAAAGATACCAGGCGTTTCCCCCTGGCGGCTCCCTCGTGCGCTCTCCTGTTCCTGCCTTTCGGTTTACCGGTGTCATTCC
