# Supplementary material for: A novel approach for T7 bacteriophage genome integration of exogenous DNA
Source: J Biol Eng. 2020 Jan 16;14:2. doi: 10.1186/s13036-019-0224-x (PMC6966851; doi:10.1186/s13036-019-0224-x)
Supplement: Supplementary file 2 — Additional file 2. Extra information about the development of T7 in vivo integration system as well as the plasmids construction schemes were described in “Development of T7 in vivo integration system.docx” and “Plasmids construction scheme.docx”, respectively. The relevant experiment results were documented in file “Figures of supplementary materials”. [file 13036_2019_224_MOESM2_ESM.zip › Additional file 2/Development of T7 in vivo integration system.docx]

As mentioned in the text, development of T7 *in vivo* integration system can be generally divided into three parts, including construction of T7∆G9, construction of T7∆G10G11-attB and construction of functional plasmids in this system. Phage recombineering method refers to a series of plasmids, which contribute to the ultimate plasmids involved in engineered T7 phage construction as well as running the integration system. Detail information about plasmids construction can be found in Additional file 2 (Plasmids construction scheme.docx). We here just listed the experiment results of the ultimate plasmids and engineered T7 phages. The figures were documented in the local file designated as “Figures of supplementary materials”.

Figure Legends of supplementary materials

**Fig. S1** Colony PCR of DH5α-pRFG9

12 colonies were randomly selected and verified by colony PCR with primer pair PG9-scF and G9U-R. The expected product was 170 bp.

**Fig. S2** Colony PCR of DH5α-pCDG9

9 colonies were randomly selected and verified by colony PCR with primer pair M13F and M13R. The expected product was 1197 bp.

**Fig. S3** BL21-pRFG9 and BL21-pCDG9 digestion assay in duplicates

pRFG9 was digested by *SalI* and Expected products were 5500 bp and 665 bp respectively. pCDG9 was digested by *EcoRV* and Expected products were 1815 bp and 1105 bp respectively.

**Fig. S4** Detect PCR of T7∆G9 recombinants

lysates from 5 reactions were subjected to PCR with primer pair PG9-scF and 5403-USP. The expected product was 1023 bp. Panel 6 indicated Blank contral with deionized water as PCR template.

**Fig. S5** Isolation of T7∆G9

a. Plague formation assay with diluted lysate from recombination reaction. Approximately 10^4 pfu phages were seeded to BL21-pCDG9 lawn b. Verification PCR of candidate T7∆G9 isolates. 10 plagues were isolated and subjected to PCR with primer pair PG9-scF and 5403-USP. The expected product was 1023 bp.

**Fig. S6** Colony PCR of BL21-pRFG10G11 and DH5α-pCDG9

5 colonies from BL21-pRFG10G11 culture plate and DH5α-pCDG9 culture plate were randomly selected respectively. The primer pair was PS-F2 and M13R with an expected product of 786 bp.

**Fig. S7** T7∆G10G11-attB plague formation assay with diluted lysate from recombination reaction

a. approximately 10^4 pfu phages seeding to BL21-C10C11 lawn. b. approximately 5x10^4 pfu phages seeding to BL21-C10C11 lawn. c. approximately 10^5 pfu phages seeding to BL21-C10C11 lawn.

**Fig. S8** Verification PCR of candidate T7∆G10G11-attB isolates

48 plagues were isolated and subjected to PCR with primer pair S23-IRS and G11-SR. The expected product was 494 bp. Red frame represented positive clones. Panel P indicated reaction lysate as positive control. Panel B indicated Blank contral with deionized water as PCR template.

**Fig. S9** Colony PCR of DH5α-pEXM3, DH5α-pEXM4, BL21-pEXM3 and DH5α- pEXM4

a. 5 colonies from DH5α-pEXM3 culture plate and DH5α-pEXM4 culture plate were randomly selected respectively. The primer pair was MC-G11-VF and EXM3-VR with an expected product of 1758 bp. b. 3 colonies from BL21-pEXM3 culture plate and BL21-pEXM4 culture plate were randomly selected respectively. The primer pair was MC-G11-VF and EXM3-VR with an expected product of 1758 bp. Panel P indicated BL21-pCDG10G11. Panel B indicated Blank contral with deionized water as PCR template.

**Fig. S10** Colony PCR of DH5α-pMCBK

5 colonies from DH5α-pMCBK culture plate were randomly selected. The primer pair was M13F and M13R with an expected product of 942 bp.
