# Supplementary material for: A novel approach for T7 bacteriophage genome integration of exogenous DNA
Source: J Biol Eng. 2020 Jan 16;14:2. doi: 10.1186/s13036-019-0224-x (PMC6966851; doi:10.1186/s13036-019-0224-x)
Supplement: Supplementary file 2 — Additional file 2. Extra information about the development of T7 in vivo integration system as well as the plasmids construction schemes were described in “Development of T7 in vivo integration system.docx” and “Plasmids construction scheme.docx”, respectively. The relevant experiment results were documented in file “Figures of supplementary materials”. [file 13036_2019_224_MOESM2_ESM.zip › Additional file 2/Plasmids construction scheme.docx]

pCDG9

Firstly, DNA fragments including the whole ORF of wild-type *gene 9* and upstream T7 promoter were amplified by PCR with templates of T7 genome DNA. The used primers were PG9-scF and PG9-scR. Next, this DNA fragments were used as templates to amplify the functional *gene 9* loci including T7 promoter as well as the intact ORF, because deletions of homologous regions flanking the key *gene 9* loci can reduce revert recombination of mutated T7 phages. The second PCR product was cloned into pTOPO-Blunt vector (Mei5 Biotechnology, China).

pCDG10

Similarly, DNA fragments including the whole ORF of wild-type *gene 10A* and upstream T7 promoter were amplified by PCR with templates of T7 genome DNA. The used primers were PS-F2 and gene10-R1. Next, the primer pair PS10A-F and PS10A-R was used to amplify the functional *gene 10* loci including T7 promoter as well as the intact ORF with the primary PCR products as template. At last, the second PCR product was cloned into pTOPO-Blunt vector (Mei5 Biotechnology, China).

pCDG10G11

The original plasmid was called ISFG10A in our laboratory, which comprised the whole ORF of wild-type *gene 10A* as well as several I-SceI restriction sites. To construct pCDG10G11, ISFG10A was digested by Cpo I (FastDigetst CpoI, Thermo scientific, the USA) as main scaffold and the insert was derived from PCR amplification by using of primer pair CDG10G11dx-IFL and CDG10G11dx-IRL with T7 genome DNA as template. Subsequently, the recombine reaction was carried out to ligate ISFG10A scaffold and subjected insert by using of ClonExpress II One Step Cloning Kit (Vazyme Biotech, China).

pRFG9

Firstly, attB site (bacterial attachment site recognized by ΦC31 integrase) was cloned downstream the truncated T7 *gene 9* ORF and subsequently, this fragment was cloned upstream the wild-type T7 *gene 10A* ORF. As consequence, the truncated *gene 9* ORF took the place of wile-type *gene 9* loci and the wild-type T7 *gene 10A* ORF functioned to supply gp10A protein and substituted the defect *gene 10* in T7Select10-3b genome.

pRFG10G11

As pCDG9 was present, it can be used to supply T7 gp9 protein in the process of T7∆G9 growth. To delete T7 *gene 10* and *gene 11* of T7∆G9, the homologous regions flanking the substitute insert were designed. The upstream homologous region was included in *gene 9* ORF while downstream homologous region was composed of *gene 12* 5’-proximal ORF. In order to sustain the normal gene 12 expression, an additional T7 promoter as well as the Tφ transcription terminator was cloned upstream the *gene 12* 5’-proximal ORF. The indispensable attB site (bacterial attachment site recognized by ΦC31 integrase) was present downstream *gene 9* ORF like pRFG9.

pEXM4

pEXM4 construction was based on pCDG10G11. ORF encoding enhanced ΦC31 integrase was cloned downstream T7 *gene 11* and the Shine-Dalgarno sequence was inserted between *gene 11* and ΦC31 integrase encoding region. As consequence, enhanced ΦC31 integrase can be produced during T7∆G10G11-attB growth under the control of T7 promoter.

pMCBK

Initially, a plasmid scaffold with Kanamycin resistant gene as well as the p15A origin of replication was employed to construct pMCBK for the sake of double-plasmid system in our integration system design. T7 gene 11 with the upstream T7 promoter and Tφ transcription terminator was cloned into the initial plasmid and then the indispensable attP site (phage attachment site) was inserted upstream T7 promoter.

pMCBK-CE Series

Based on pMCBK, exogenous DNA with various lengths were inserted between attP site and T7 promoter. These inserts were amplified by PCR from SpCas9 ORF and they had the common regions that allowed to conduct PCR with the same primer pair and the same products.
