# Supplementary figures and images for: A novel approach for T7 bacteriophage genome integration of exogenous DNA
Source: J Biol Eng. 2020 Jan 16;14:2. doi: 10.1186/s13036-019-0224-x (PMC6966851; doi:10.1186/s13036-019-0224-x)

12 colonies

M bp

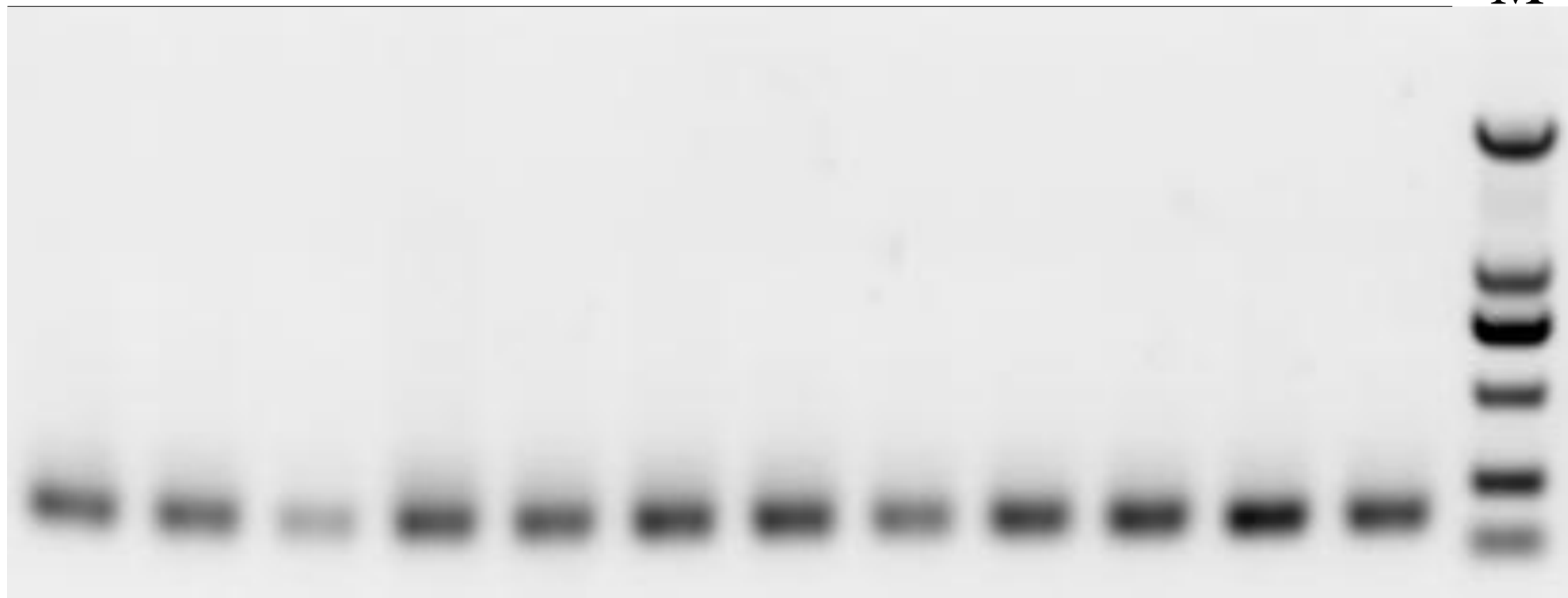

-250

-100

Supplement: Supplementary file 2 — Additional file 2. Extra information about the development of T7 in vivo integration system as well as the plasmids construction schemes were described in “Development of T7 in vivo integration system.docx” and “Plasmids construction scheme.docx”, respectively. The relevant experiment results were documented in file “Figures of supplementary materials”. [file 13036_2019_224_MOESM2_ESM.zip › Additional file 2/Figures of supplementary materials/Fig. S1.pdf]

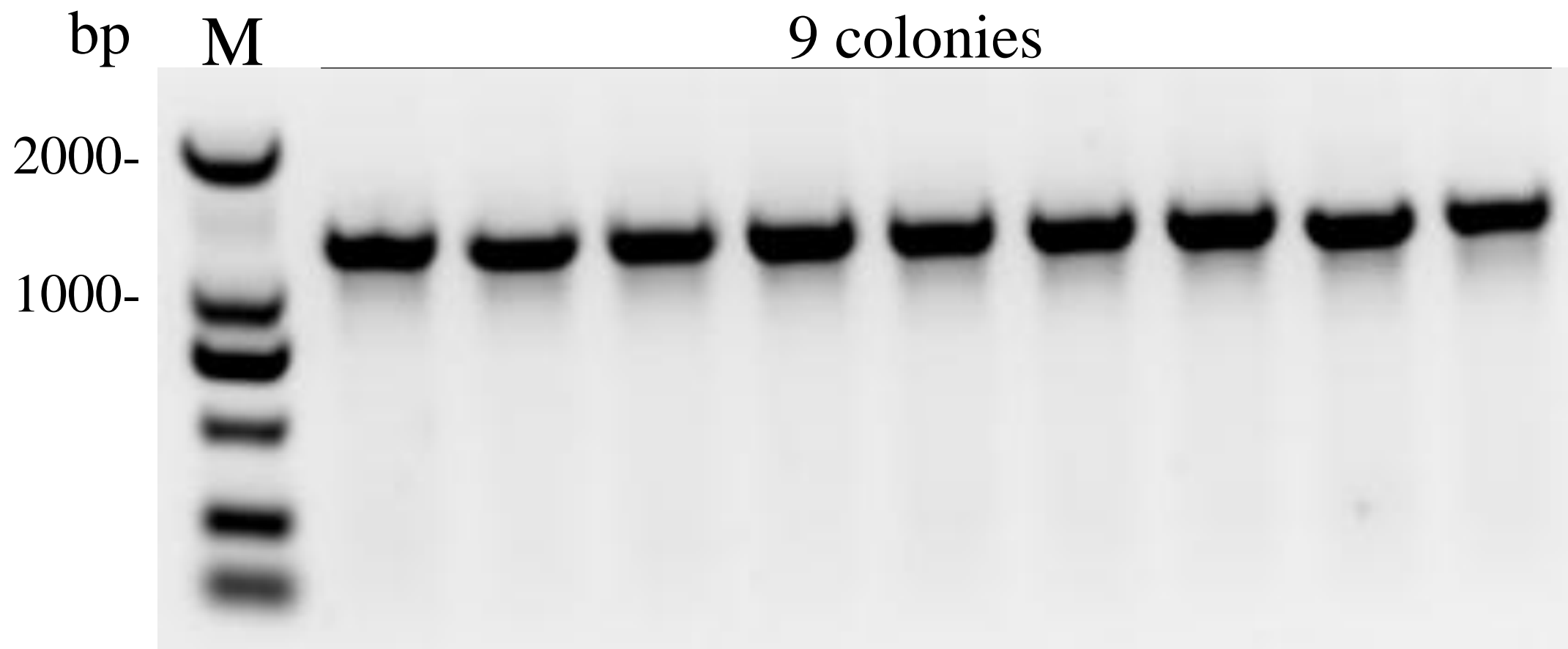

Supplement: Supplementary file 2 — Additional file 2. Extra information about the development of T7 in vivo integration system as well as the plasmids construction schemes were described in “Development of T7 in vivo integration system.docx” and “Plasmids construction scheme.docx”, respectively. The relevant experiment results were documented in file “Figures of supplementary materials”. [file 13036_2019_224_MOESM2_ESM.zip › Additional file 2/Figures of supplementary materials/Fig. S2.pdf]

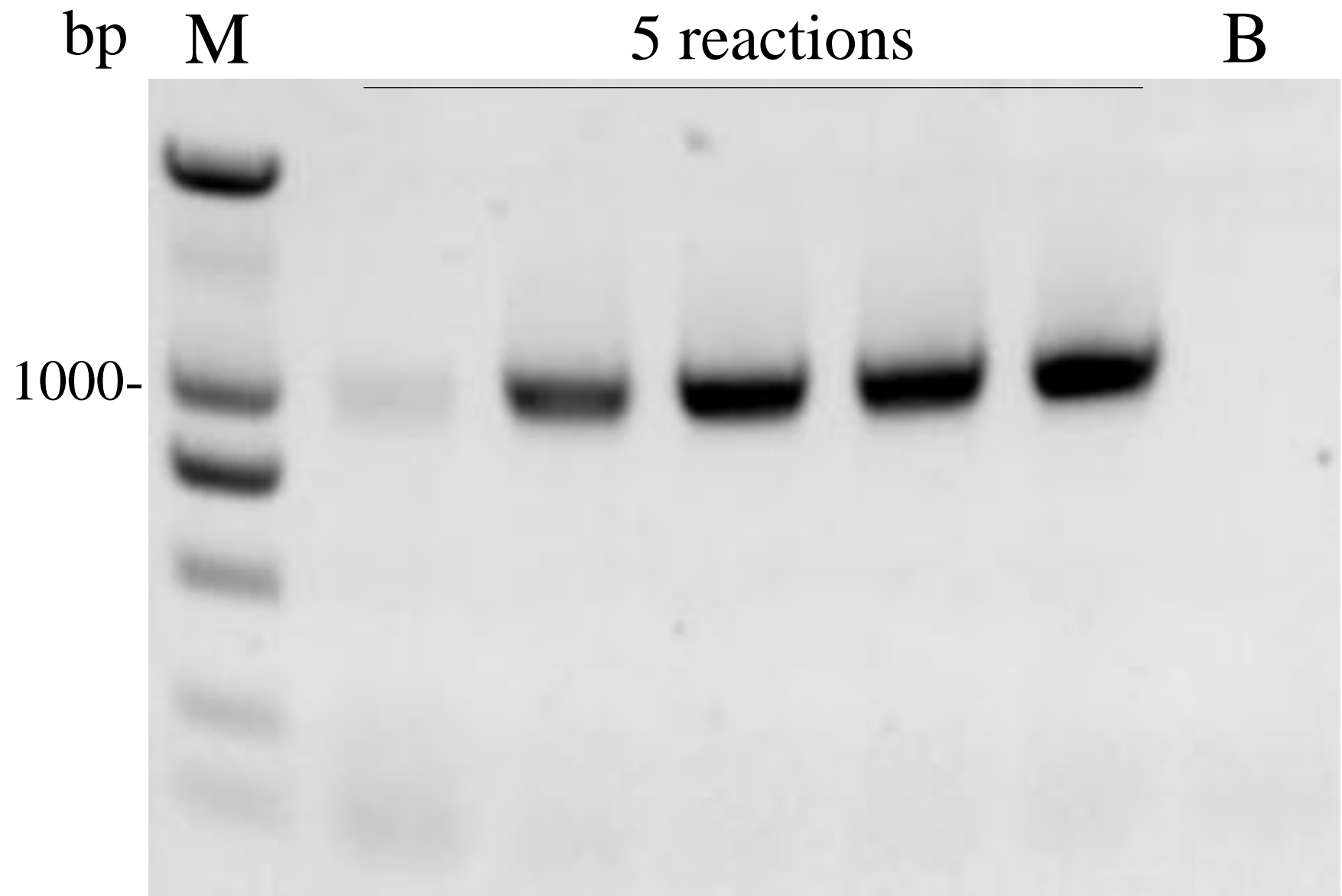

Supplement: Supplementary file 2 — Additional file 2. Extra information about the development of T7 in vivo integration system as well as the plasmids construction schemes were described in “Development of T7 in vivo integration system.docx” and “Plasmids construction scheme.docx”, respectively. The relevant experiment results were documented in file “Figures of supplementary materials”. [file 13036_2019_224_MOESM2_ESM.zip › Additional file 2/Figures of supplementary materials/Fig. S4.pdf]

a

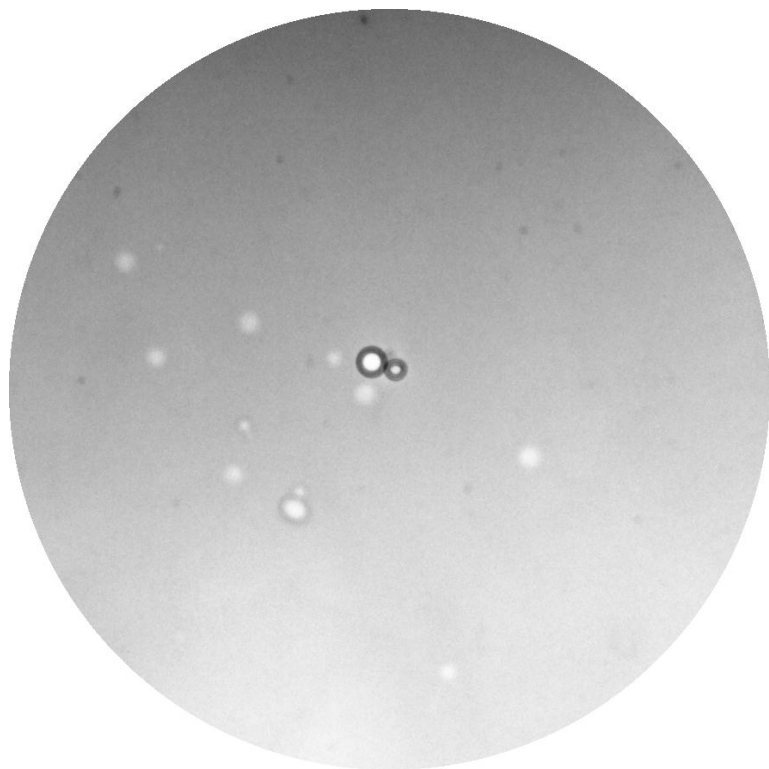

b

bp

M

10 plaques

1000-

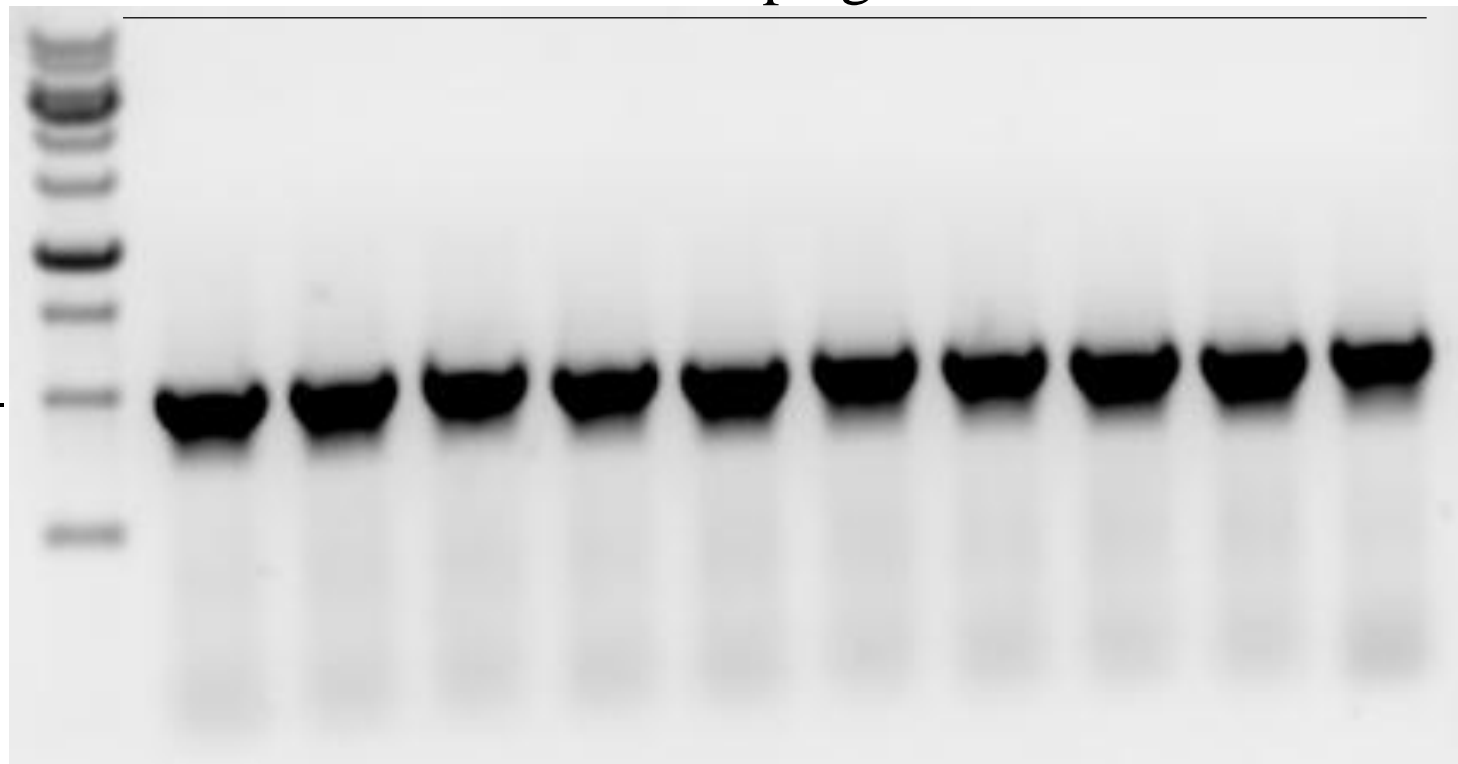

Supplement: Supplementary file 2 — Additional file 2. Extra information about the development of T7 in vivo integration system as well as the plasmids construction schemes were described in “Development of T7 in vivo integration system.docx” and “Plasmids construction scheme.docx”, respectively. The relevant experiment results were documented in file “Figures of supplementary materials”. [file 13036_2019_224_MOESM2_ESM.zip › Additional file 2/Figures of supplementary materials/Fig. S5.pdf]

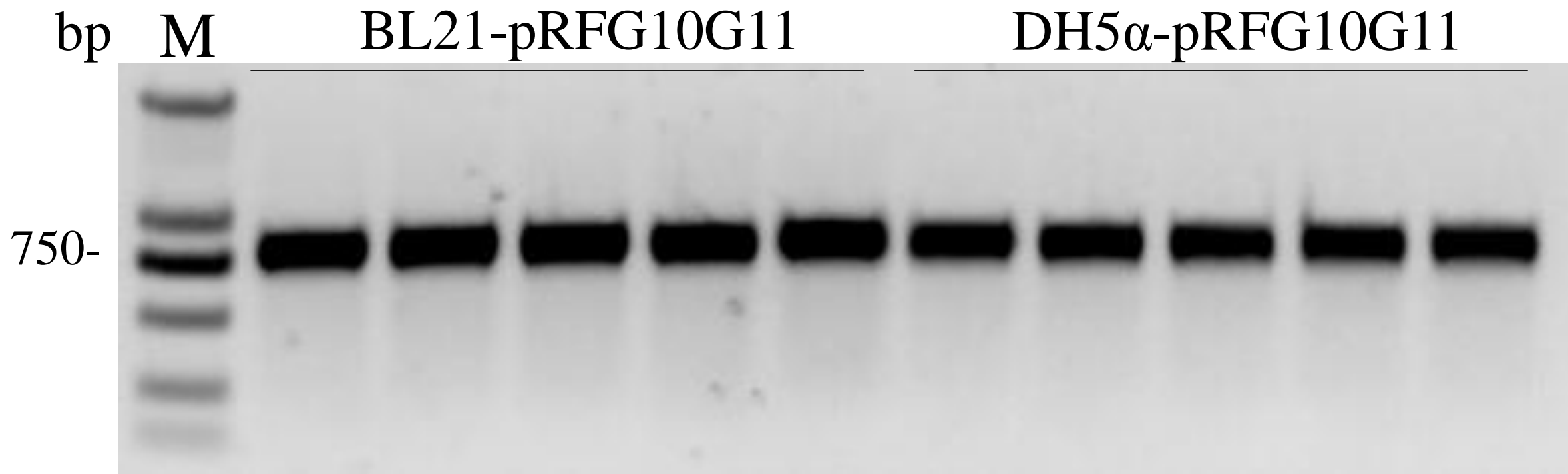

Supplement: Supplementary file 2 — Additional file 2. Extra information about the development of T7 in vivo integration system as well as the plasmids construction schemes were described in “Development of T7 in vivo integration system.docx” and “Plasmids construction scheme.docx”, respectively. The relevant experiment results were documented in file “Figures of supplementary materials”. [file 13036_2019_224_MOESM2_ESM.zip › Additional file 2/Figures of supplementary materials/Fig. S6.pdf]

a

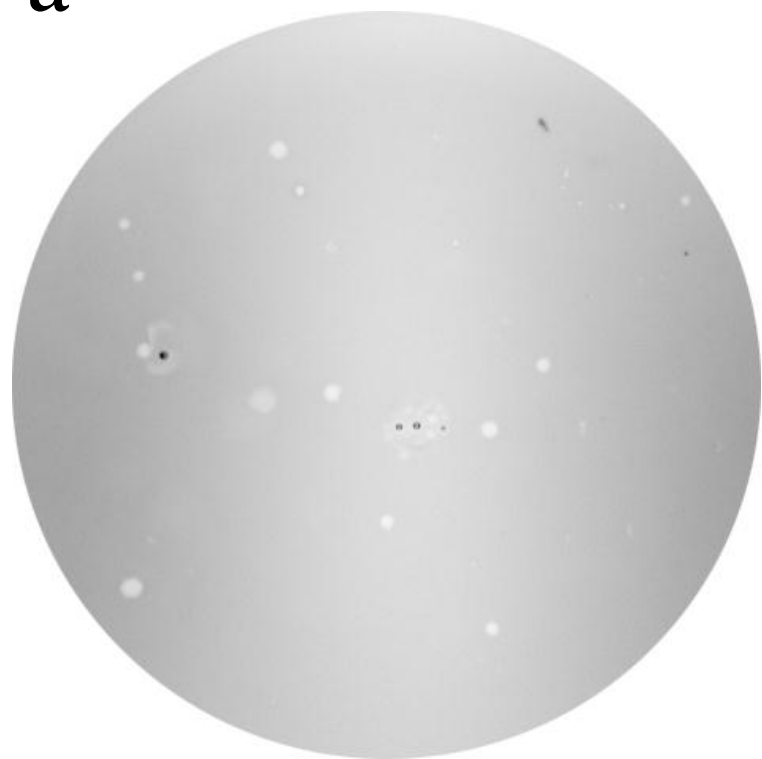

b

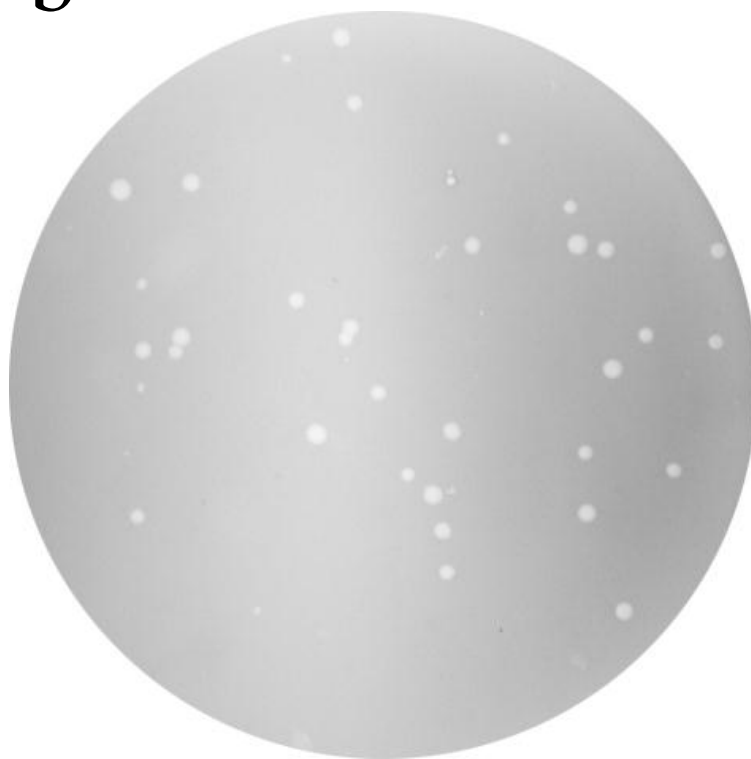

c

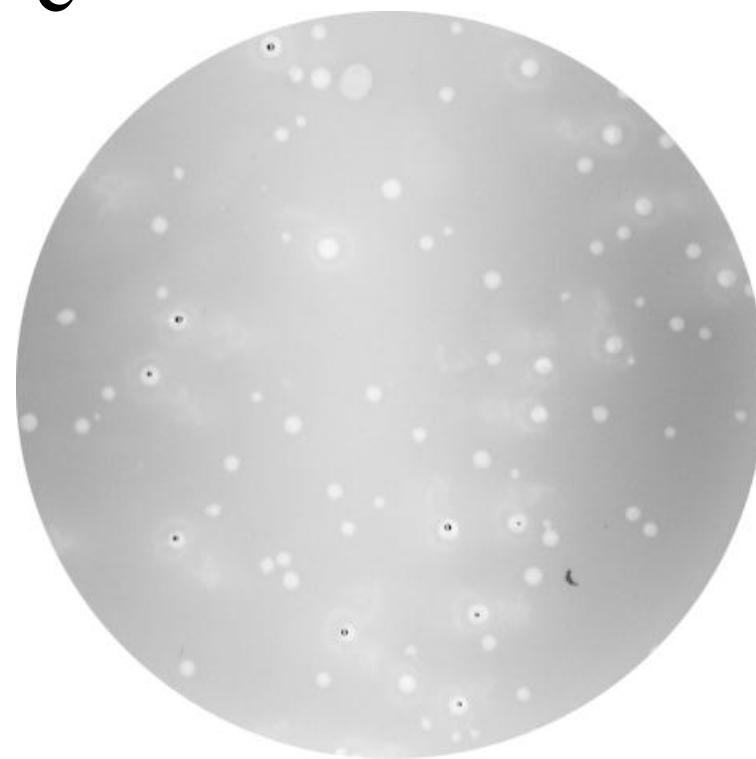

Supplement: Supplementary file 2 — Additional file 2. Extra information about the development of T7 in vivo integration system as well as the plasmids construction schemes were described in “Development of T7 in vivo integration system.docx” and “Plasmids construction scheme.docx”, respectively. The relevant experiment results were documented in file “Figures of supplementary materials”. [file 13036_2019_224_MOESM2_ESM.zip › Additional file 2/Figures of supplementary materials/Fig. S7.pdf]

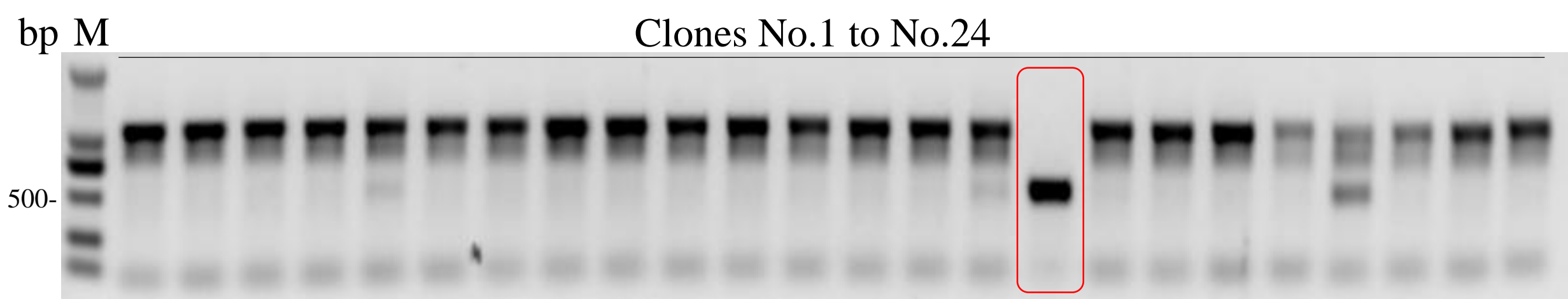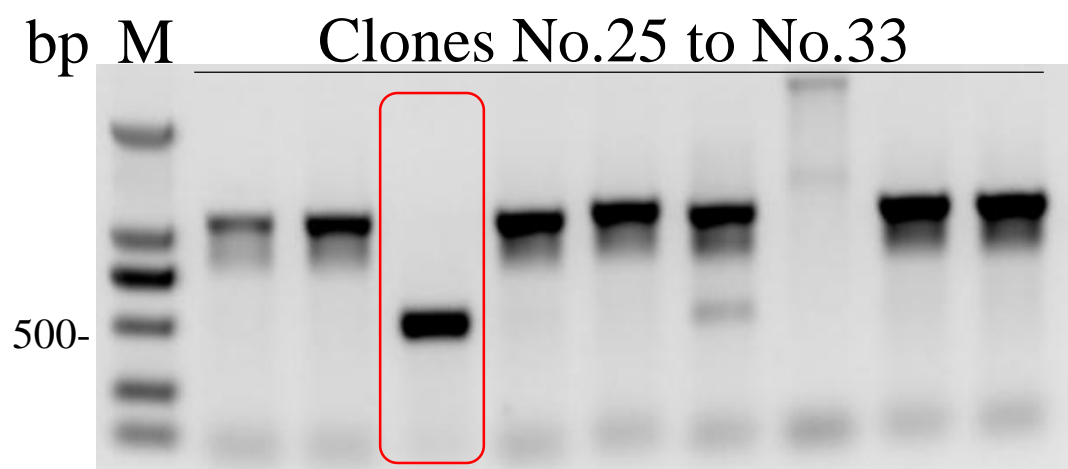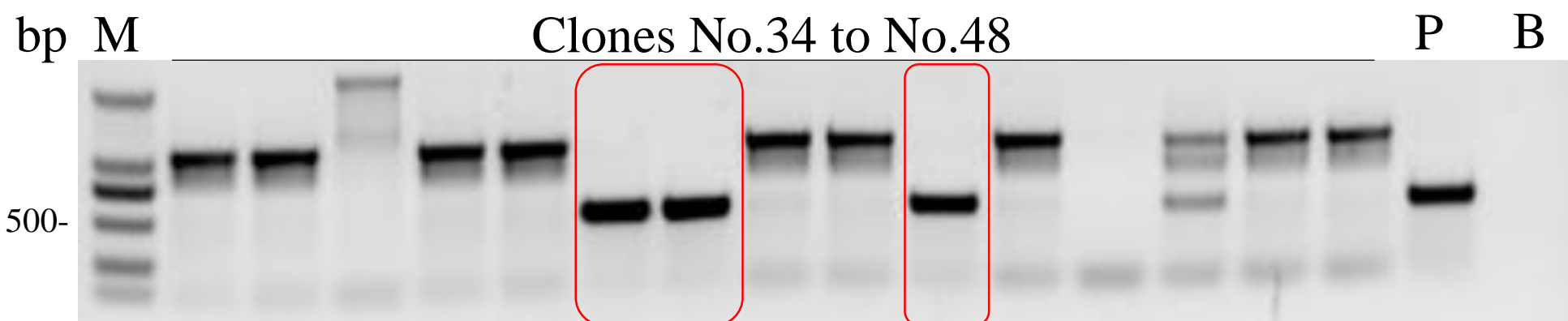

Supplement: Supplementary file 2 — Additional file 2. Extra information about the development of T7 in vivo integration system as well as the plasmids construction schemes were described in “Development of T7 in vivo integration system.docx” and “Plasmids construction scheme.docx”, respectively. The relevant experiment results were documented in file “Figures of supplementary materials”. [file 13036_2019_224_MOESM2_ESM.zip › Additional file 2/Figures of supplementary materials/Fig. S8.pdf]

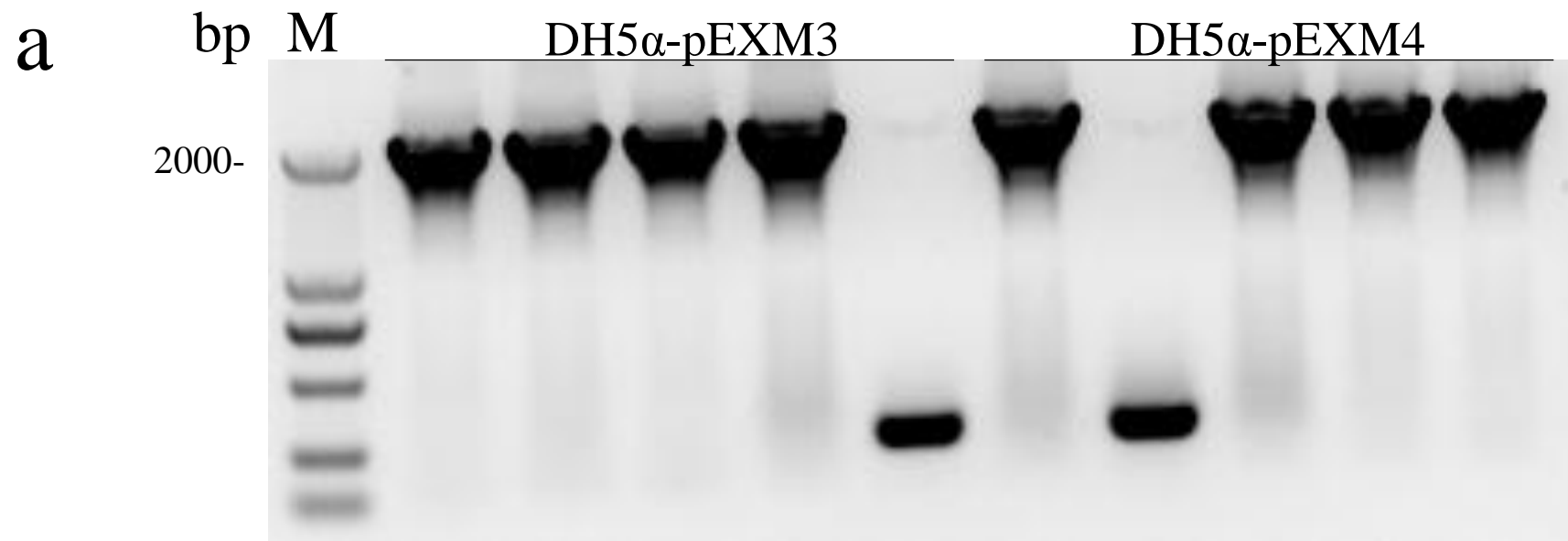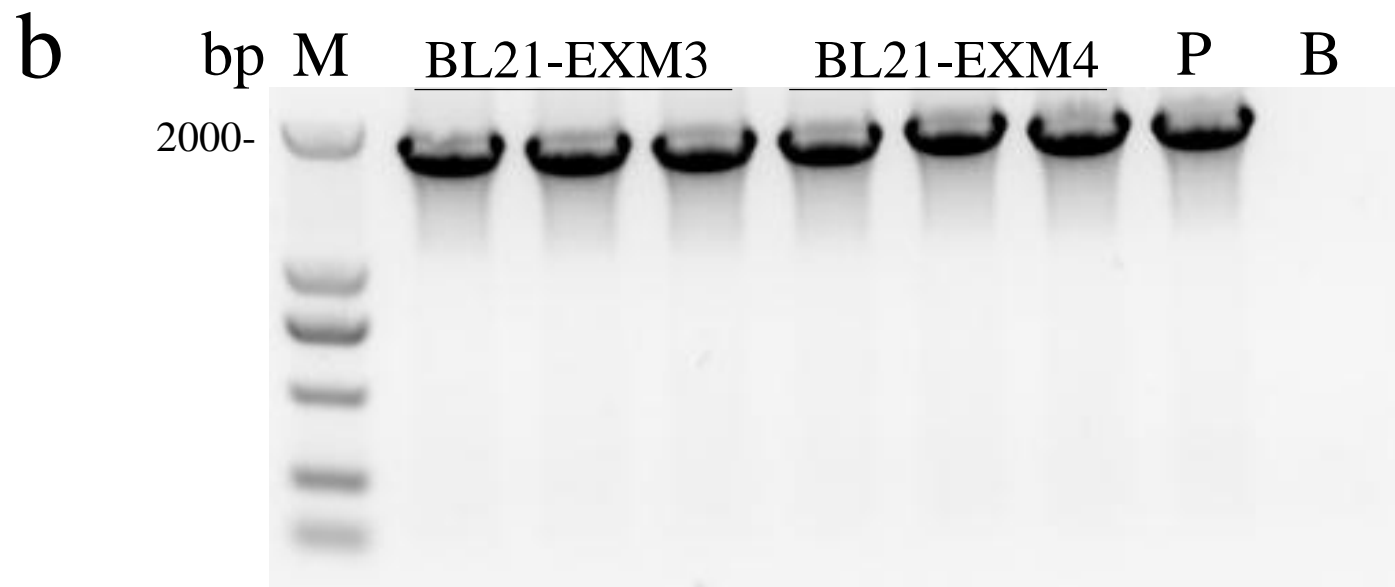

Supplement: Supplementary file 2 — Additional file 2. Extra information about the development of T7 in vivo integration system as well as the plasmids construction schemes were described in “Development of T7 in vivo integration system.docx” and “Plasmids construction scheme.docx”, respectively. The relevant experiment results were documented in file “Figures of supplementary materials”. [file 13036_2019_224_MOESM2_ESM.zip › Additional file 2/Figures of supplementary materials/Fig. S9.pdf]
