# Supplementary material for: A novel approach for T7 bacteriophage genome integration of exogenous DNA
Source: J Biol Eng. 2020 Jan 16;14:2. doi: 10.1186/s13036-019-0224-x (PMC6966851; doi:10.1186/s13036-019-0224-x)
Supplement: Supplementary file 3 — Additional file 3. Sequencing results of engineered T7 phage and the positive recombinants harboring the exogenous DNA were documented in three files respectively, these files comprised the crude statistics as well as the processed sequences in docx format. [file 13036_2019_224_MOESM3_ESM.zip › Additional file 3/CE-Int T7 sequencing/CE-Int T7 sequencing.docx]

Expected Sequence of CE1-Int T7 genome amplified by PS-F2 and IT-CR

**CCTTGAGACGCACAACCCTGAGGCTGCACAGTCGCTGGATAATGCGTTGACCAATCGTGACTTAGCGACCGTTAAGGCTATCATCAACTTGGCTGGTGAGTCTCGCGCTAAGGCGTTCGGTCGTAAGCCAACTCGTAGTGTGACTAATCGTGCTATTCCGGCTAAACCTCAGGCTACCAAGCGTGAAGGCTTTGCGGACCGTAGCGAGATGATTAAAGCTATGAGTGACCCTCGGTATCGCACAGATGCCAACTATCGTCGTCAAGTCGAACAGAAAGTAATCGATTCGAACTTC**TAACTAGATCTGTGCTCAAAGAGGAATCTATCAAGGGCGACACGCGAATTCGATATCAAGCTTATGTAGGTGACGGT**CTCGAAGCCGCGGTGCGGGTGCCAGGGCGTGCCCTTGAGTTCTCTCAGTTGGGGGCGTAGGGTCGCCGACATGACACAAGGGGTT**AAGCTTGATATCAGTTCAAGGTTCTGGGAAATACAGACCGCCACAGTATCAAAAAAAATCTTATAGGGGCTCTTTTATTTGACAGTGGAGAGACAGCGGAAGCGACTCGCCTCAAACGGACAGCTCGTAGAAGGTATACACGTCGGAAGAATCGTATTTGTTATCTACAGGAGATTTTTTCAAATGAGATGGCGAAAGTAGATGATAGTTTCTTTCATCGACTTGAAGAGTCTTTTTTGGTGGAAGAAGACAAGAAGCATGAACGTCATCCTATTTTTGGAAATATAGTAGATGAAGTTGCTTATCATGAGAAATATCCAACTATCTATCATCTGCGAAAAAAATTGGTAGATTCTACTGATAAAGCGGATTTGCGCTTAATCTATTTGGCCTTAGCGCATATGATTAAGTTTCGTGGTCATTTTTTGATTGAGGGAGATTTAAATCCTGATAATAGTGATGTGGACAAACTATTTATCCAGTTGGTACAAACCTACAATCAATTATTTGAAGAAAACCCTATTAACGCAAGTGGAGTAGATGCTAAAGCGATTCTTTCTGCACGATTGAGTAAATCAAGACGATTAGAAAATCTCATTGCTCAGCTCCCCGGTGAGAAGAAAAATGGCTTATTTGGGAATCTCATTGCTTTGTCATTGGGTTTGACCCCTAATTTTAAATCAAATTTTGATTTGGCAGAAGATGCTAAATTACAGCTTTCAAAAGATACTTACGATGATGATTTAGATAATTTATTGGCGCAAATTGGAGATCAATATGCTGATTTGTTTTTGGCAGCTAAGAATTTATCAGATGCTATTTTACTTTCAGATATCCTAAGAGTAAATACTGAAATAACTAAGGCTCCCCTATCAGC

>1.1 F

GACGCTGGTATGCGTTGACCATCGTGACTTAGCGACCGTTAAGGCTATCATCAACTTGGCTGGTGAGTCTCGCGCTAAGGCGTTCGGTCGTAAGCCAACTCGTAGTGTGACTAATCGTGCTATTCCGGCTAAACCTCAGGCTACCAAGCGTGAAGGCTTTGCGGACCGTAGCGAGATGATTAAAGCTATGAGTGACCCTCGGTATCGCACAGATGCCAACTATCGTCGTCAAGTCGAACAGAAAGTAATCGATTCGAACTTCTAACTAGATCTGTGCTCAAAGAGGAATCTATCAAGGGCGACACGCGAATTCGATATCGCGGCCGCGATATCAAGCTTATGTAGGTGACGGTCTCGAAGCCGCGGTGCGGGTGCCAGGGCGTGCCCTTGAGTTCTCTCAGTTGGGGGCGTAGGGTCGCCGACATGACTCAAGGGGTTAAGCTTGATATCAGTTCAAGGTTCTGGGAAATACAGACCGCCACAGTATCAAAAAAAATCTTATAGGGGCTCTTTTATTTGACAGTGGAGAGACAGCGGAAGCGACTCGCCTCAAACGGACAGCTCGTAGAAGGTATACACGTCGGAAGAATCGTATTTGTTATCTACAGGAGATTTTTTCAAATGAGATGGCGAAAGTAGATGATAGTTTCTTTCATCGACTTGAAGAGTCTTTTTTGGTGGAAGAAGACAAGAAGCATGAACGTCATCCTATTTTTGGAAATATAGTAGATGAAGTTGCTTATCATGAGAAATATCCAACTATCTATCATCTGCGAAAAGAATTGGTAGATTCTACTGATAAAGCGGATTTGCGCTTAATCTATTTGGCCTTAGCGCATATGATTAAGTTTCGTGGTCATTTTTTGATTGAGGGAGATTTAAATCCTGATAATAG

>1.1 R

CAAGGGGTTAAGCTTGATATCAGTTCAAGGTTCTGGGAAATCCAGACCGCCCCCAGTTTCAAAAAAATTCTTATAGGGGCTCTTTTATTTGACAGTGGAGAGACAGCGGAAGCCGCTCGCCTCAAACGGACAGCTCGTAGGAAGGTATACACGTCGGAAGAATCGTATTTGTTATCTACAGGAGATTTTTTCAAATGAGATGGCGAAAGTAGATGATAGTTTCTTTCATCGACTTGAAGAGTCTTTTTTGGTGGAAGAAGACAAGAAGCATGAACGTCATCCTTATTTTTGGAAATATAGTAGATGAAGTTGCTTATCATGAGAAATATCCAACTATCTATCATCTGCGAAAAAAATTGGTAGATTCTACTGATAAAGCGGATTTGCGCTTAATCTATTTGGCCTTAGCGCATATGATTAAGTTTCGTGGTCATTTTTTGATTGAGGGAGATTTAAATCCTGATAATAGTGATGTGGACAAACTATTTATCCAGTTGGTACAAACCTACAATCAATTATTTGAAGAAAACCCTATTAACGCAAGTGGAGTAGATGCTAAAGCGATTCTTTCTGCACGATTGAGTAAATCAAGACGATTAGAAAATCTCATTGCTCAGCTCCCCGGTGAGAAGAAAAATGGCTTATTTGGGAATCTCATTGCTTTGTCATTGGGTTTGACCCCTAATTTTAAATCAAATTTTGATTTGGCAGAAGATGCTAAATTACAGCTTTCAAAAGATACTTACGATGATGATTTAGATAATTTATTGGCGCAAATTGGAGATCAATATGCTGATTTGTTTTTGGCAGCTAAGAAATTTATCAGATGCTATTTACTTCAGAATCGAAGAGGTGATAC

>1.1

GACGCTGGTATGCGTTGACCATCGTGACTTAGCGACCGTTAAGGCTATCATCAACTTGGCTGGTGAGTCTCGCGCTAAGGCGTTCGGTCGTAAGCCAACTCGTAGTGTGACTAATCGTGCTATTCCGGCTAAACCTCAGGCTACCAAGCGTGAAGGCTTTGCGGACCGTAGCGAGATGATTAAAGCTATGAGTGACCCTCGGTATCGCACAGATGCCAACTATCGTCGTCAAGTCGAACAGAAAGTAATCGATTCGAACTTCTAACTAGATCTGTGCTCAAAGAGGAATCTATCAAGGGCGACACGCGAATTCGATATCGCGGCCGCGATATCAAGCTTATGTAGGTGACGGT**CTCGAAGCCGCGGTGCGGGTGCCAGGGCGTGCCCTTGAGTTCTCTCAGTTGGGGGCGTAGGGTCGCCGACATGACA/TCAAGGGGTT**AAGCTTGATATCAGTTCAAGGTTCTGGGAAATACAGACCGCCACAGTATCAAAAAAAATCTTATAGGGGCTCTTTTATTTGACAGTGGAGAGACAGCGGAAGCGACTCGCCTCAAACGGACAGCTCGTAGAAGGTATACACGTCGGAAGAATCGTATTTGTTATCTACAGGAGATTTTTTCAAATGAGATGGCGAAAGTAGATGATAGTTTCTTTCATCGACTTGAAGAGTCTTTTTTGGTGGAAGAAGACAAGAAGCATGAACGTCATCCTTATTTTTGGAAATATAGTAGATGAAGTTGCTTATCATGAGAAATATCCAACTATCTATCATCTGCGAAAAAAATTGGTAGATTCTACTGATAAAGCGGATTTGCGCTTAATCTATTTGGCCTTAGCGCATATGATTAAGTTTCGTGGTCATTTTTTGATTGAGGGAGATTTAAATCCTGATAATAGTGATGTGGACAAACTATTTATCCAGTTGGTACAAACCTACAATCAATTATTTGAAGAAAACCCTATTAACGCAAGTGGAGTAGATGCTAAAGCGATTCTTTCTGCACGATTGAGTAAATCAAGACGATTAGAAAATCTCATTGCTCAGCTCCCCGGTGAGAAGAAAAATGGCTTATTTGGGAATCTCATTGCTTTGTCATTGGGTTTGACCCCTAATTTTAAATCAAATTTTGATTTGGCAGAAGATGCTAAATTACAGCTTTCAAAAGATACTTACGATGATGATTTAGATAATTTATTGGCGCAAATTGGAGATCAATATGCTGATTTGTTTTTGGCAGCTAAGAAATTTATCAGATGCTATTTACTTCAGAATCGAAGAGGTGATAC

>1.2 F

CGGGCTATGCGTTGACAATCGTGACTTAGCGACCGTTAAGGCTATCATCAACTTGGCTGGTGAGTCTCGCGCTAAGGCGTTCGGTCGTAAGCCAACTCGTAGTGTGACTAATCGTGCTATTCCGGCTAAACCTCAGGCTACCAAGCGTGAAGGCTTTGCGGACCGTAGCGAGATGATTAAAGCTATGAGTGACCCTCGGTATCGCACAGATGCCAACTATCGTCGTCAAGTCGAACAGAAAGTAATCGATTCGAACTTCTAACTAGATCTGTGCTCAAAGAGGAATCTATCAAGGGCGACACGCGAATTCGATATCGCGGCCGCGATATCAAGCTTATGTAGGTGACGGTCTCGAAGCCGCGGTGCGGGTGCCAGGGCGTGCCCTTGAGTTCTCTCAGTTGGGGGCGTAGGGTCGCCGACATGACTCAAGGGGTTAAGCTTGATATCAGTTCAAGGTTCTGGGAAATACAGACCGCCACAGTATCAAAAAAAATCTTATAGGGGCTCTTTTATTTGACAGTGGAGAGACAGCGGAAGCGACTCGCCTCAAACGGACAGCTCGTAGAAGGTATACACGTCGGAAGAATCGTATTTGTTATCTACAGGAGATTTTTTCAAATGAGATGGCGAAAGTAGATGATAGTTTCTTTCATCGACTTGAAGAGTCTTTTTTGGTGGAAGAAGACAAGAAGCATGAACGTCATCCTATTTTTGGAAATATAGTAGATGAAGTTGCTTATCATGAGAAATATCCAACTATCTATCATCTGCGAAGAAAATTGGTAGATTCTACTGATAAGGCGGAGTTGCGCTTAGTCTATTTGGCCTTAGCGCATATGATTAAGTTTCATGGTCATTGTGTGATTGAGGAAGATTTAAGTCCTGATAAGAGTGATGG

>1.2 R

AGCGTCGCCGACATGACTCAAGGGGTTAAGCTTGATATCAGTTCAAGGTTCTGCGAAATACAGACCGCCACAGTATCAAAAAAAATCTTATAGGGGCTCTTTTATCTGACAGTGGAGAGACAGCGGAAGCGACTCGCCTCAAACGGACAGCTCGTAGAAGGTATACACGTCGGAAGAATCGTATCTGTTATCTACAGGAGATTTTTTCAAATGAGATGGCGAAAGTAGATGATAGTTTCTTTCATCGACTTGAAGAGTCTTTTTTGGTGGAAGAAGACAAGAAGCATGAACGTCATCCTATTTTTGGAAATATAGTAGATGAAGTTGCTTATCATGAGAAATATCCAACTATCTATCATCTGCGAAAAAAATTGGTAGATTCTACTGATAAAGCGGATTTGCGCTTAATCTATTTGGCCTTAGCGCATATGATTAAGTTTCGTGGTCATTTTTTGATTGAGGGAGATTTAAATCCTGATAATAGTGATGTGGACAAACTATTTATCCAGTTGGTACAAACCTACAATCAATTATTTGAAGAAAACCCTATTAACGCAAGTGGAGTAGATGCTAAAGCGATTCTTTCTGCACGATTGAGTAAATCAAGACGATTAGAAAATCTCATTGCTCAGCTCCCCGGTGAGAAGAAAAATGGCTTATTTGGGAATCTCATTGCTTTGTCATTGGGTTTGACCCCTAATTTTAAATCAAATTTTGATTTGGCAGAAGATGCTAAATTACAGCTTTCAAAAGATACTTACGATGATGATTTAGATAATTTATTGGCGCAAATTGGAGATCAATATGCTGATTTGTTTTTGGCAGCTAAGAATTTATCAGATGCTATTTACTTCAGATTCCTAAGAGTAATTAC

>1.2

CGGGCTATGCGTTGACAATCGTGACTTAGCGACCGTTAAGGCTATCATCAACTTGGCTGGTGAGTCTCGCGCTAAGGCGTTCGGTCGTAAGCCAACTCGTAGTGTGACTAATCGTGCTATTCCGGCTAAACCTCAGGCTACCAAGCGTGAAGGCTTTGCGGACCGTAGCGAGATGATTAAAGCTATGAGTGACCCTCGGTATCGCACAGATGCCAACTATCGTCGTCAAGTCGAACAGAAAGTAATCGATTCGAACTTCTAACTAGATCTGTGCTCAAAGAGGAATCTATCAAGGGCGACACGCGAATTCGATATCGCGGCCGCGATATCAAGCTTATGTAGGTGACGGT**CTCGAAGCCGCGGTGCGGGTGCCAGGGCGTGCCCTTGAGTTCTCTCAGTTGGGGGCGTAGGGTCGCCGACATGACA/TCAAGGGGTT**AAGCTT**GATATCAGTTCAAGGTTCTGGGAAATACAGACCGCCACAGTAT**CAAAAAAAATCTTATAGGGGCTCTTTTATTTGACAGTGGAGAGACAGCGGAAGCGACTCGCCTCAAACGGACAGCTCGTAGAAGGTATACACGTCGGAAGAATCGTATCTGTTATCTACAGGAGATTTTTTCAAATGAGATGGCGAAAGTAGATGATAGTTTCTTTCATCGACTTGAAGAGTCTTTTTTGGTGGAAGAAGACAAGAAGCATGAACGTCATCCTATTTTTGGAAATATAGTAGATGAAGTTGCTTATCATGAGAAATATCCAACTATCTATCATCTGCGAAAAAAATTGGTAGATTCTACTGATAAAGCGGATTTGCGCTTAATCTATTTGGCCTTAGCGCATATGATTAAGTTTCGTGGTCATTTTTTGATTGAGGGAGATTTAAATCCTGATAATAGTGATGTGGACAAACTATTTATCCAGTTGGTACAAACCTACAATCAATTATTTGAAGAAAACCCTATTAACGCAAGTGGAGTAGATGCTAAAGCGATTCTTTCTGCACGATTGAGTAAATCAAGACGATTAGAAAATCTCATTGCTCAGCTCCCCGGTGAGAAGAAAAATGGCTTATTTGGGAATCTCATTGCTTTGTCATTGGGTTTGACCCCTAATTTTAAATCAAATTTTGATTTGGCAGAAGATGCTAAATTACAGCTTTCAAAAGATACTTACGATGATGATTTAGATAATTTATTGGCGCAAATTGGAGATCAATATGCTGATTTGTTTTTGGCAGCTAAGAATTTATCAGATGCTATTTACTTCAGATTCCTAAGAGTAATTAC

Expected Sequence of CE2-Int T7 genome amplified by PS-F2 and IT-CR

CCTTGAGACGCACAACCCTGAGGCTGCACAGTCGCTGGATAATGCGTTGACCAATCGTGACTTAGCGACCGTTAAGGCTATCATCAACTTGGCTGGTGAGTCTCGCGCTAAGGCGTTCGGTCGTAAGCCAACTCGTAGTGTGACTAATCGTGCTATTCCGGCTAAACCTCAGGCTACCAAGCGTGAAGGCTTTGCGGACCGTAGCGAGATGATTAAAGCTATGAGTGACCCTCGGTATCGCACAGATGCCAACTATCGTCGTCAAGTCGAACAGAAAGTAATCGATTCGAACTTCTAACTAGATCTGTGCTCAAAGAGGAATCTATCAAGGGCGACACGCGAATTCGATATCAAGCTTATGTAGGTGACGGT**CTCGAAGCCGCGGTGCGGGTGCCAGGGCGTGCCCTTGAGTTCTCTCAGTTGGGGGCGTAGGGTCGCCGACATGACACAAGGGGTT**AAGCTTGATATCAGTTCAAGGTTCTGGGAAATACAGACCGCCACAGTATCAAAAAAAATCTTATAGGGGCTCTTTTATTTGACAGTGGAGAGACAGCGGAAGCGACTCGCCTCAAACGGACAGCTCGTAGAAGGTATACACGTCGGAAGAATCGTATTTGTTATCTACAGGAGATTTTTTCAAATGAGATGGCGAAAGTAGATGATAGTTTCTTTCATCGACTTGAAGAGTCTTTTTTGGTGGAAGAAGACAAGAAGCATGAACGTCATCCTATTTTTGGAAATATAGTAGATGAAGTTGCTTATCATGAGAAATATCCAACTATCTATCATCTGCGAAAAAAATTGGTAGATTCTACTGATAAAGCGGATTTGCGCTTAATCTATTTGGCCTTAGCGCATATGATTAAGTTTCGTGGTCATTTTTTGATTGAGGGAGATTTAAATCCTGATAATAGTGATGTGGACAAACTATTTATCCAGTTGGTACAAACCTACAATCAATTATTTGAAGAAAACCCTATTAACGCAAGTGGAGTAGATGCTAAAGCGATTCTTTCTGCACGATTGAGTAAATCAAGACGATTAGAAAATCTCATTGCTCAGCTCCCCGGTGAGAAGAAAAATGGCTTATTTGGGAATCTCATTGCTTTGTCATTGGGTTTGACCCCTAATTTTAAATCAAATTTTGATTTGGCAGAAGATGCTAAATTACAGCTTTCAAAAGATACTTACGATGATGATTTAGATAATTTATTGGCGCAAATTGGAGATCAATATGCTGATTTGTTTTTGGCAGCTAAGAATTTATCAGATGCTATTTTACTTTCAGATATCCTAAGAGTAAATACTGAAATAACTAAGGCTCCCCTATCAGC

>2.1 F

GTTCCTGGTATGCGTTGACCAATCGTGACTTAGCGACCGTTAAGGCTATCATCAACTTGGCTGGTGAGTCTCGCGCTAAGGCGTTCGGTCGTAAGCCAACTCGTAGTGTGACTAATCGTGCTATTCCGGCTAAACCTCAGGCTACCAAGCGTGAAGGCTTTGCGGACCGTAGCGAGATGATTAAAGCTATGAGTGACCCTCGGTATCGCACAGATGCCAACTATCGTCGTCAAGTCGAACAGAAAGTAATCGATTCGAACTTCTAACTAGATCTGTGCTCAAAGAGGAATCTATCAAGGGCGACACGCGAATTCGATATCGCGGCCGCGATATCAAGCTTATGTAGGTGACGGTCTCGAAGCCGCGGTGCGGGTGCCAGGGCGTGCCCTTGAGTTCTCTCAGTT**GGGGGCGTAGGGTCGCCGACATGACACAAGGGGTT**AAGCTTGATATCAGTTCAAGGTTCTGGGAAATACAGACCGCCACAGTATCAAAAAAAATCTTATAGGGGCTCTTTTATTTGACAGTGGAGAGACAGCGGAAGCGACTCGCCTCAAACGGACAGCTCGTAGAAGGTATACACGTCGGAAGAATCGTATTTGTTATCTACAGGAGATTTTTTCAAATGAGATGGCGAAAGTAGATGATAGTTTCTTTCATCGACTTGAAGAGTCTTTTTTGGTGGAAGAAGACAAGAAGCATGAACGTCATCCTATTTTTGGAAATATAGTAGATGAAGTTGCTTATCATGAGAAATATCCAACTATCTATCATCTGCGAAAAAAATTGGTAGATTCTACTGATAAAGCGGATTTGCGCTTAATCTATTTGGCCTTAGCGCATATGATTAAGTTTCGTGGTCATTGTTTGATTGAGGGAGATTTAAATCCTGATAATAGTGATGTGGACAAA

>2.1 R

CGTGCCCTTGAGTTCTCTCAGTT**GGGGGCGTAGGGTCGCCGACATGACACAAGGGGTT**AAGCTTGATATCAGTTCAAGGTTCTGGGAAATACAGACCGCCACAGTATCAAAAAAAATCTTATAGGGGCTCTTTTATTTGACAGTGGAGAGACAGCGGAAGCGACTCGCCTCAAACGGACAGCTCGTAGAAGGTATACACGTCGGAAGAATCGTATTTGTTATCTACAGGAGATTTTTTCAAATGAGATGGCGAAAGTAGATGATAGTTTCTTTCATCGACTTGAAGAGTCTTTTTTGGTGGAAGAAGACAAGAAGCATGAACGTCATCCTATTTTTGGAAATATAGTAGATGAAGTTGCTTATCATGAGAAATATCCAACTATCTATCATCTGCGAAAAAAATTGGTAGATTCTACTGATAAAGCGGATTTGCGCTTAATCTATTTGGCCTTAGCGCATATGATTAAGTTTCGTGGTCATTTTTTGATTGAGGGAGATTTAAATCCTGATAATAGTGATGTGGACAAACTATTTATCCAGTTGGTACAAACCTACAATCAATTATTTGAAGAAAACCCTATTAACGCAAGTGGAGTAGATGCTAAAGCGATTCTTTCTGCACGATTGAGTAAATCAAGACGATTAGAAAATCTCATTGCTCAGCTCCCCGGTGAGAAGAAAAATGGCTTATTTGGGAATCTCATTGCTTTGTCATTGGGTTTGACCCCTAATTTTAAATCAAATTTTGATTTGGCAGAAGATGCTAAATTACAGCTTTCAAAAGATACTTACGATGATGATTTAGATAATTTATTGGCGCAAATTGGAGATCAATATGCTGATTTGTTTTTGGCAGCTAAGAATTTATCAGATGCTATTTACTTCAGATATCCTAAGAGTTAATAC

>2.1

GTTCCTGGTATGCGTTGACCAATCGTGACTTAGCGACCGTTAAGGCTATCATCAACTTGGCTGGTGAGTCTCGCGCTAAGGCGTTCGGTCGTAAGCCAACTCGTAGTGTGACTAATCGTGCTATTCCGGCTAAACCTCAGGCTACCAAGCGTGAAGGCTTTGCGGACCGTAGCGAGATGATTAAAGCTATGAGTGACCCTCGGTATCGCACAGATGCCAACTATCGTCGTCAAGTCGAACAGAAAGTAATCGATTCGAACTTCTAACTAGATCTGTGCTCAAAGAGGAATCTATCAAGGGCGACACGCGAATTCGATATCGCGGCCGCGATATCAAGCTTATGTAGGTGACGGT**CTCGAAGCCGCGGTGCGGGTGCCAGGGCGTGCCCTTGAGTTCTCTCAGTTGGGGGCGTAGGGTCGCCGACATGACACAAGGGGTT**AAGCTTGATATCAGTTCAAGGTTCTGGGAAATACAGACCGCCACAGTATCAAAAAAAATCTTATAGGGGCTCTTTTATTTGACAGTGGAGAGACAGCGGAAGCGACTCGCCTCAAACGGACAGCTCGTAGAAGGTATACACGTCGGAAGAATCGTATTTGTTATCTACAGGAGATTTTTTCAAATGAGATGGCGAAAGTAGATGATAGTTTCTTTCATCGACTTGAAGAGTCTTTTTTGGTGGAAGAAGACAAGAAGCATGAACGTCATCCTATTTTTGGAAATATAGTAGATGAAGTTGCTTATCATGAGAAATATCCAACTATCTATCATCTGCGAAAAAAATTGGTAGATTCTACTGATAAAGCGGATTTGCGCTTAATCTATTTGGCCTTAGCGCATATGATTAAGTTTCGTGGTCATTTTTTGATTGAGGGAGATTTAAATCCTGATAATAGTGATGTGGACAAACTATTTATCCAGTTGGTACAAACCTACAATCAATTATTTGAAGAAAACCCTATTAACGCAAGTGGAGTAGATGCTAAAGCGATTCTTTCTGCACGATTGAGTAAATCAAGACGATTAGAAAATCTCATTGCTCAGCTCCCCGGTGAGAAGAAAAATGGCTTATTTGGGAATCTCATTGCTTTGTCATTGGGTTTGACCCCTAATTTTAAATCAAATTTTGATTTGGCAGAAGATGCTAAATTACAGCTTTCAAAAGATACTTACGATGATGATTTAGATAATTTATTGGCGCAAATTGGAGATCAATATGCTGATTTGTTTTTGGCAGCTAAGAATTTATCAGATGCTATTTACTTCAGATATCCTAAGAGTTAATAC

>2.2 F

GGTCACTGGGATATGCGTTGACCAATCGTGACTTAGCGACCGTTAAGGCTATCATCAACTTGGCTGGTGAGTCTCGCGCTAAGGCGTTCGGTCGTAAGCCAACTCGTAGTGTGACTAATCGTGCTATTCCGGCTAAACCTCAGGCTACCAAGCGTGAAGGCTTTGCGGACCGTAGCGAGATGATTAAAGCTATGAGTGACCCTCGGTATCGCACAGATGCCAACTATCGTCGTCAAGTCGAACAGAAAGTAATCGATTCGAACTTCTAACTAGATCTGTGCTCAAAGAGGAATCTATCAAGGGCGACACGCGAATTCGATATCGCGGCCGCGATATCAAGCTTATGTAGGTGACGGT**CTCGAAGCCGCGGTGCGGGTGCCAGGGCGTGCCCTTGAGTTCTCTCAGTTGGGGGCGTAGGGTCGCCGACATGACACAAGGGGTT**AAGCTTGATATCAGTTCAAGGTTCTGGGAAATACAGACCGCCACAGTATCAAAAAAAATCTTATAGGGGCTCTTTTATTTGACAGTGGAGAGACAGCGGAAGCGACTCGCCTCAAACGGACAGCTCGTAGAAGGTATACACGTCGGAAGAATCGTATTTGTTATCTACAGGAGATTTTTTCAAATGAGATGGCGAAAGTAGATGATAGTTTCTTTCATCGACTTGAAGAGTCTTTTTTGGTGGAAGAAGACAAGAAGCATGAACGTCATCCTATTTTTGGAAATATAGTAGATGAAGTTGCTTATCATGAGAAATATCCAACTATCTATCATCTGCGAAAAAAATTGGTAGATTCTACTGATAAAGCGGATTTGCGCTTAATCTATTTGGCCTTAGCGCATATGATTAAAGTTTCGTGGTCATTTTTTGATTGAGGGAGATTTAAATCCTGATAATAGTGATG

>2.2 R

CCGGTTCCCAGGGCGTGCCCTTGATTTCTCTCAGTT**GGGGGCGTAGGGTCGCCGACATGACACAAGGGGTT**AAGCTTGATATCAGTTCAAGGTTCTGGGAAATACAGACCGCCACAGTTTCAAAAAAAATCTTATAGGGGCTCTTTTATTTGACAGTGGAGAGACAGCGGAAGCGACTCGCCTCAAACGGACAGCTCGTAGAAGGTATACACGTCGGAAGAATCGTATTTGTTATCTACAGGAGATTTTTTCAAATGAGATGGCGAAAGTAGATGATAGTTTCTTTCATCGACTTGAAGAGTCTTTTTTGGTGGAAGAAGACAAGAAGCATGAACGTCATCCTATTTTTGGAAATATAGTAGATGAAGTTGCTTATCATGAGAAATATCCAACTATCTATCATCTGCGAAAAAAATTGGTAGATTCTACTGATAAAGCGGATTTGCGCTTAATCTATTTGGCCTTAGCGCATATGATTAAGTTTCGTGGTCATTTTTTGATTGAGGGAGATTTAAATCCTGATAATAGTGATGTGGACAAACTATTTATCCAGTTGGTACAAACCTACAATCAATTATTTGAAGAAAACCCTATTAACGCAAGTGGAGTAGATGCTAAAGCGATTCTTTCTGCACGATTGAGTAAATCAAGACGATTAGAAAATCTCATTGCTCAGCTCCCCGGTGAGAAGAAAAATGGCTTATTTGGGAATCTCATTGCTTTGTCATTGGGTTTGACCCCTAATTTTAAATCAAATTTTGATTTGGCAGAAGATGCTAAATTACAGCTTTCAAAAGATACTTACGATGATGATTTAGATAATTTATTGGCGCAAATTGGAGATCAATATGCTGATTTGTTTTTGGCAGCTAAGAATTTATCAGATGCTATTTACTTCAGATTCCTAAGAGTAATAC

>2.2

GGTCACTGGGATATGCGTTGACCAATCGTGACTTAGCGACCGTTAAGGCTATCATCAACTTGGCTGGTGAGTCTCGCGCTAAGGCGTTCGGTCGTAAGCCAACTCGTAGTGTGACTAATCGTGCTATTCCGGCTAAACCTCAGGCTACCAAGCGTGAAGGCTTTGCGGACCGTAGCGAGATGATTAAAGCTATGAGTGACCCTCGGTATCGCACAGATGCCAACTATCGTCGTCAAGTCGAACAGAAAGTAATCGATTCGAACTTCTAACTAGATCTGTGCTCAAAGAGGAATCTATCAAGGGCGACACGCGAATTCGATATCGCGGCCGCGATATCAAGCTTATGTAGGTGACGGTCTCGAAGCCGCGGTGCGGGTGCCAGGGCGTGCCCTTGAGTTCTCTCAGTT**GGGGGCGTAGGGTCGCCGACATGACACAAGGGGTT**AAGCTTGATATCAGTTCAAGGTTCTGGGAAATACAGACCGCCACAGTATCAAAAAAAATCTTATAGGGGCTCTTTTATTTGACAGTGGAGAGACAGCGGAAGCGACTCGCCTCAAACGGACAGCTCGTAGAAGGTATACACGTCGGAAGAATCGTATTTGTTATCTACAGGAGATTTTTTCAAATGAGATGGCGAAAGTAGATGATAGTTTCTTTCATCGACTTGAAGAGTCTTTTTTGGTGGAAGAAGACAAGAAGCATGAACGTCATCCTATTTTTGGAAATATAGTAGATGAAGTTGCTTATCATGAGAAATATCCAACTATCTATCATCTGCGAAAAAAATTGGTAGATTCTACTGATAAAGCGGATTTGCGCTTAATCTATTTGGCCTTAGCGCATATGATTAAGTTTCGTGGTCATTTTTTGATTGAGGGAGATTTAAATCCTGATAATAGTGATGTGGACAAACTATTTATCCAGTTGGTACAAACCTACAATCAATTATTTGAAGAAAACCCTATTAACGCAAGTGGAGTAGATGCTAAAGCGATTCTTTCTGCACGATTGAGTAAATCAAGACGATTAGAAAATCTCATTGCTCAGCTCCCCGGTGAGAAGAAAAATGGCTTATTTGGGAATCTCATTGCTTTGTCATTGGGTTTGACCCCTAATTTTAAATCAAATTTTGATTTGGCAGAAGATGCTAAATTACAGCTTTCAAAAGATACTTACGATGATGATTTAGATAATTTATTGGCGCAAATTGGAGATCAATATGCTGATTTGTTTTTGGCAGCTAAGAATTTATCAGATGCTATTTACTTCAGATTCCTAAGAGTAATAC

Expected Sequence of CE3-Int T7 genome amplified by PS-F2 and IT-CR

CCTTGAGACGCACAACCCTGAGGCTGCACAGTCGCTGGATAATGCGTTGACCAATCGTGACTTAGCGACCGTTAAGGCTATCATCAACTTGGCTGGTGAGTCTCGCGCTAAGGCGTTCGGTCGTAAGCCAACTCGTAGTGTGACTAATCGTGCTATTCCGGCTAAACCTCAGGCTACCAAGCGTGAAGGCTTTGCGGACCGTAGCGAGATGATTAAAGCTATGAGTGACCCTCGGTATCGCACAGATGCCAACTATCGTCGTCAAGTCGAACAGAAAGTAATCGATTCGAACTTCTAACTAGATCTGTGCTCAAAGAGGAATCTATCAAGGGCGACACGCGAATTCGATATCAAGCTTATGTAGGTGACGGT**CTCGAAGCCGCGGTGCGGGTGCCAGGGCGTGCCCTTGAGTTCTCTCAGTTGGGGGCGTAGGGTCGCCGACATGACACAAGGGGTT**AAGCTTGATATCAAACCCTATTAACGCAAGTGGAGTAGATGCTAAAGCGATTCTTTCTGCACGATTGAGTAAATCAAGACGATTAGAAAATCTCATTGCTCAGCTCCCCGGTGAGAAGAAAAATGGCTTATTTGGGAATCTCATTGCTTTGTCATTGGGTTTGACCCCTAATTTTAAATCAAATTTTGATTTGGCAGAAGATGCTAAATTACAGCTTTCAAAAGATACTTACGATGATGATTTAGATAATTTATTGGCGCAAATTGGAGATCAATATGCTGATTTGTTTTTGGCAGCTAAGAATTTATCAGATGCTATTTTACTTTCAGATATCCTAAGAGTAAATACTGAAATAACTAAGGCTCCCCTATCAGC

>3.1 F

CGGGATATGCGTTGACCATCGTGACTTAGCGACCGTTAAGGCTATCATCAACTTGGCTGGTGAGTCTCGCGCTAAGGCGTTCGGTCGTAAGCCAACTCGTAGTGTGACTAATCGTGCTATTTCCGGCTAAACCTCAGGCTACCAAGCGTGAAGGCTTTGCGGACCGTAGCGAGATGATTAAAGCTATGAGTGACCCTCGGTATCGCACAGATGCCAACTATCGTCGTCAAGTCGAACAGAAAGTAATCGATTCGAACTTCTAACTAGATCTGTGCTCAAAGAGGAATCTATCAAGGGCGACACGCGAATTCGATATCGCGGCCGCGATATCAAGCTTATGTAGGTGACGGTCTCGAAGCCGCGGTGCGGGTGCCAGGGCGTGCCCTTGAGTTCTCTCAGTT**GGGGGCGTAGGGTCGCCGACATGACACAAGGGGTT**AAGCTTGATATCAAACCCTATTAACGCAAGTGGAGTAGATGCTAAAGCGATTCTTTCTGCACGATTGAGTAAATCAAGACGATTAGAAAATCTCATTGCTCAGCTCCCCGGTGAGAAGAAAAATGGCTTATTTGGGAATCTCATTGCTTTGTCATTGGGTTTGACCCCTAATTTTAAATCAAATTTTGATTTGGCAGAAGATGCTAAATTACAGCTTTCAAAAGATACTTACGATGATGATTTAGATAATTTATTGGCGCAAATTGGAGATCAATATGCTGATTTGTTTTTGGCAGCTAAGAATTTATCAGATGCTATTTTACTTTCAGATATCCTAAGAGTAAATACTGAATAACTAGC

>3.1 R

CCCCCCAGAGCTCACAGTCGCTGGATAATGCGTTGACCAATCGTGACTTAGCGACCGTTAAGGCTATCATCAACTTGGCTGGTGAGTCTCGCGCTAAGGCGTTCGGTCGTAAGCCAACTCGTAGTGTGACTAATCGTGCTATTCCGGCTAAACCTCAGGCTACCAAGCGTGAAGGCTTTGCGGACCGTAGCGAGATGATTAAAGCTATGAGTGACCCTCGGTATCGCACAGATGCCAACTATCGTCGTCAAGTCGAACAGAAAGTAATCGATTCGAACTTCTAACTAGATCTGTGCTCAAAGAGGAATCTATCAAGGGCGACACGCGAATTCGATATCGCGGCCGCGATATCAAGCTTATGTAGGTGACGGTCTCGAAGCCGCGGTGCGGGTGCCAGGGCGTGCCCTTGAGTTCTCTCAGTT**GGGGGCGTAGGGTCGCCGACATGACACAAGGGGTT**AAGCTTGATATCAAACCCTATTAACGCAAGTGGAGTAGATGCTAAAGCGATTCTTTCTGCACGATTGAGTAAATCAAGACGATTAGAAAATCTCATTGCTCAGCTCCCCGGTGAGAAGAAAAATGGCTTATTTGGGAATCTCATTGCTTTGTCATTGGGTTTGACCCCTAATTTTAAATCAAATTTTGATTTGGCAGAAGATGCTAAATTACAGCTTTCAAAAGATACTTACGATGATGATTTAGATAATTTATTGGCGCAAATTGGAGATCAATATGCTGATTTGTTTTTGGCAGCTAAGAATTTATCAGATGCTATTTACTTCAGATTCGAAGAGTAATA

>3.1

CCCCCCAGAGCTCACAGTCGCTGGATAATGCGTTGACCAATCGTGACTTAGCGACCGTTAAGGCTATCATCAACTTGGCTGGTGAGTCTCGCGCTAAGGCGTTCGGTCGTAAGCCAACTCGTAGTGTGACTAATCGTGCTATTCCGGCTAAACCTCAGGCTACCAAGCGTGAAGGCTTTGCGGACCGTAGCGAGATGATTAAAGCTATGAGTGACCCTCGGTATCGCACAGATGCCAACTATCGTCGTCAAGTCGAACAGAAAGTAATCGATTCGAACTTCTAACTAGATCTGTGCTCAAAGAGGAATCTATCAAGGGCGACACGCGAATTCGATATCGCGGCCGCGATATCAAGCTTATGTAGGTGACGGT**CTCGAAGCCGCGGTGCGGGTGCCAGGGCGTGCCCTTGAGTTCTCTCAGTTGGGGGCGTAGGGTCGCCGACATGACACAAGGGGTT**AAGCTTGATATCAAACCCTATTAACGCAAGTGGAGTAGATGCTAAAGCGATTCTTTCTGCACGATTGAGTAAATCAAGACGATTAGAAAATCTCATTGCTCAGCTCCCCGGTGAGAAGAAAAATGGCTTATTTGGGAATCTCATTGCTTTGTCATTGGGTTTGACCCCTAATTTTAAATCAAATTTTGATTTGGCAGAAGATGCTAAATTACAGCTTTCAAAAGATACTTACGATGATGATTTAGATAATTTATTGGCGCAAATTGGAGATCAATATGCTGATTTGTTTTTGGCAGCTAAGAATTTATCAGATGCTATTTTACTTTCAGATATCCTAAGAGTAAATACTGAATAACTAGC

>3.2 F

CCTGGGTATGCGTTGACCATCGTGACTTAGCGACCGTTAAGGCTATCATCAACTTGGCTGGTGAGTCTCGCGCTAAGGCGTTCGGTCGTAAGCCAACTCGTAGTGTGACTAATCGTGCTATTCCGGCTAAACCTCAGGCTACCAAGCGTGAAGGCTTTGCGGACCGTAGCGAGATGATTAAAGCTATGAGTGACCCTCGGTATCGCACAGATGCCAACTATCGTCGTCAAGTCGAACAGAAAGTAATCGATTCGAACTTCTAACTAGATCTGTGCTCAAAGAGGAATCTATCAAGGGCGACACGCGAATTCGATATCGCGGCCGCGATATCAAGCTTATGTAGGTGACGGTCTCGAAGCCGCGGTGCGGGTGCCAGGGCGTGCCCTTGAGTTCTCTCAGTT**GGGGGCGTAGGGTCGCCGACATGACACAAGGGGTT**AAGCTTGATATCAAACCCTATTAACGCAAGTGGAGTAGATGCTAAAGCGATTCTTTCTGCACGATTGAGTAAATCAAGACGATTAGAAAATCTCATTGCTCAGCTCCCCGGTGAGAAGAAAAATGGCTTATTTGGGAATCTCATTGCTTTGTCATTGGGTTTGACCCCTAATTTTAAATCAAATTTTGATTTGGCAGAAGATGCTAAATTACAGCTTTCAAAAGATACTTACGATGATGATTTAGATAATTTATTGGCGCAAATTGGAGATCAATATGCTGATTTGTTTTTGGCAGCTAAGAATTTATCAGATGCTATTTTACTTTCAGATATCCTAAGAGTAAATACTGAAATACTAAGCCGCCTC

>3.2 R

CCCTAGCTCAAGTCGCTGGATAATGCGTTGACCAATCGTGACTTAGCGACCGTTAAGGCTATCATCAACTTGGCTGGTGAGTCTCGCGCTAAGGCGTTCGGTCGTAAGCCAACTCGTAGTGTGACTAATCGTGCTATTCCGGCTAAACCTCAGGCTACCAAGCGTGAAGGCTTTGCGGACCGTAGCGAGATGATTAAAGCTATGAGTGACCCTCGGTATCGCACAGATGCCAACTATCGTCGTCAAGTCGAACAGAAAGTAATCGATTCGAACTTCTAACTAGATCTGTGCTCAAAGAGGAATCTATCAAGGGCGACACGCGAATTCGATATCGCGGCCGCGATATCAAGCTTATGTAGGTGACGGTCTCGAAGCCGCGGTGCGGGTGCCAGGGCGTGCCCTTGAGTTCTCTCAGTT**GGGGGCGTAGGGTCGCCGACATGACACAAGGGGTT**AAGCTTGATATCAAACCCTATTAACGCAAGTGGAGTAGATGCTAAAGCGATTCTTTCTGCACGATTGAGTAAATCAAGACGATTAGAAAATCTCATTGCTCAGCTCCCCGGTGAGAAGAAAAATGGCTTATTTGGGAATCTCATTGCTTTGTCATTGGGTTTGACCCCTAATTTTAAATCAAATTTTGATTTGGCAGAAGATGCTAAATTACAGCTTTCAAAAGATACTTACGATGATGATTTAGATAATTTATTGGCGCAAATTGGAGATCAATATGCTGATTTGTTTTTGGCAGCTAAAGAAATTTATCAGATGCTATTTACTTCAGAATCGTAAGAGTAATG

>3.2

CCCTAGCTCAAGTCGCTGGATAATGCGTTGACCAATCGTGACTTAGCGACCGTTAAGGCTATCATCAACTTGGCTGGTGAGTCTCGCGCTAAGGCGTTCGGTCGTAAGCCAACTCGTAGTGTGACTAATCGTGCTATTCCGGCTAAACCTCAGGCTACCAAGCGTGAAGGCTTTGCGGACCGTAGCGAGATGATTAAAGCTATGAGTGACCCTCGGTATCGCACAGATGCCAACTATCGTCGTCAAGTCGAACAGAAAGTAATCGATTCGAACTTCTAACTAGATCTGTGCTCAAAGAGGAATCTATCAAGGGCGACACGCGAATTCGATATCGCGGCCGCGATATCAAGCTTATGTAGGTGACGGT**CTCGAAGCCGCGGTGCGGGTGCCAGGGCGTGCCCTTGAGTTCTCTCAGTTGGGGGCGTAGGGTCGCCGACATGACACAAGGGGTT**AAGCTTGATATCAAACCCTATTAACGCAAGTGGAGTAGATGCTAAAGCGATTCTTTCTGCACGATTGAGTAAATCAAGACGATTAGAAAATCTCATTGCTCAGCTCCCCGGTGAGAAGAAAAATGGCTTATTTGGGAATCTCATTGCTTTGTCATTGGGTTTGACCCCTAATTTTAAATCAAATTTTGATTTGGCAGAAGATGCTAAATTACAGCTTTCAAAAGATACTTACGATGATGATTTAGATAATTTATTGGCGCAAATTGGAGATCAATATGCTGATTTGTTTTTGGCAGCTAAGAATTTATCAGATGCTATTTTACTTTCAGATATCCTAAGAGTAAATACTGAAATACTAAGCCGCCTC

Expected Sequence of CE3-Int T7 genome amplified by InsertSeq-F1 and InsertSeq-R1

GGACCGTAGCGAGATGATTAAAGCTATGAGTGACCCTCGGTATCGCACAGATGCCAACTATCGTCGTCAAGTCGAACAGAAAGTAATCGATTCGAACTTCTAACTAGATCTGTGCTCAAAGAGGAATCTATCAAGGGCGACACGCGAATTCGATATCAAGCTTATGTAGGTGACGGTCTCGAAGCCGCGGTGCGGGTGCCAGGGCGTGCCCTTGAGTTCTCTCAGTTGGGGGCGTAGGGTCGCCGACATGACACAAGGGGTTAAGCTTGATATCAAACCCTATTAACGCAAGTGGAGTAGATGCTAAAGCGATTCTTTCTGCACGATTGAGTAAATCAAGACGATTAGAAAATCTCATTGCTCAGCTCCCCGGTGAGAAGAAAAATGGCTTATTTGGGAATCTCATTGCTTTGTCATTGGGTTTGACCCCTAATTTTAAATCAAATTTTGATTTGGCAGAAGATGCTAAATTACAGCTTTCAAAAGATACTTACGATGATGATTTAGATAATTTATTGGCGCAAATTGGAGATCAATATGCTGATTTGTTTTTGGCAGCTAAGAATTTATCAGATGCTATTTTACTTTCAGATATCCTAAGAGTAAATACTGAAATAACTAAGGCTCCCCTATCAGCTTCAATGATTAAACGCTACGATGAACATCATCAAGACTTGACTCTTTTAAAAGCTTTAGTTCGACAACAACTTCCAGAAAAGTATAAAGAAATCTTTTTTGATCAATCAAAAAACGGATATGCAGGTTATATTGATGGGGGAGCTAGCCAAGAAGAATTTTATAAATTTATCAAACCAATTTTAGAAAAAATGGATGGTACTGAGGAATTATTGGTGAAACTAAATCGTGAAGATTTGCTGCGCAAGCAACGGACCTTTGACAACGGCTCTATTCCCCATCAAATTCACTTGGGTGAGCTGCATGCTATTTTGAGAAGACAAGAAGACTTTTATCCATTTTTAAAAGACAATCGTGAGAAGATTGAAAAAATCTTGACTTTTCGAATTCCTTATTATGTTGGTCCATTGGCGCGTGGCAATAGTCGTTTTGCATGGATGACTCGGAAGTCTGAAGAAACAATTACCCCATGGAATTTTGAAGAAGTTGTCGATAAAGGTGCTTCAGCTCAATCATTTATTGAACGCATGACAGCGTTTGATAAAAATCTTCCAAATGAAAAAGTACTACCAAAACATAGTTTGCTTTATGAGTATTTTACGGTTTATAACGAATTGACAAAGGTCAAATATGTTACTGAAGGAATGCGAAAACCAGCATTTCTTTCAGGTGAACAGAAGAAAGCCATTGTTGATTTACTCTTCAAAACAAATCGAAAAGTAACCGTTAAGCAATTAAAAGAAGATTATTTCAAAAAAATAGAATGTTTTGATAGTGTTGAAATTTCAGGAGTTGAAGATAGATTTAATGCTTCATTAGGTACCTACCATGATTTGCTAAAAATTATTAAAGATAAAGATTTTTTGGATAATGAAGAAAATGAAGATATCTTAGAGGATATTGTTTTAACATTGACCTTATTTGAAGATAGGGAGATGATTGAGGAAAGACTTAAAACATATGCTCACCTCTTTGATGATAAGGTGATGAAACAGCTTAAACGTCGCCGTTATACTGGTTGGGGAGCGTTGTCTCGAAAATTGATTAATGGTATTAGGGATAAGCAATCTGGCAAAACAATATTAGATTTTTTGAAATCAGATGGTTTTGCCAATCGCAATTTTATGGCGCTGATCCATGATGATAGTTTGACATTTAAAGAAGACATTCAAAAAGCACAAGTGTCCGGACAAGGCGATAGTTTACATGAACATATTGCAAATTTAGCTGGTAGCCCTGCTATTAAAAAAGGTATTTTACAGACTGTAAAAGTTGTTGATGAATTGGTCAAAGTAATGGGGCGGCATAAGCCAGAAAATATCGTTATTGAAATGGCACGTGAAAATCAGACAACTCAAAAGGGCCAGAAAAATTCGCGAGAGCGTATGAAACGAATCGAAGAAGGTATCAAAGAATTAGGAAGTCAGATTCTTAAAGAGCATCCTGTTGAAAATACTCAATTGCAAAATGAAAAGCTCTATCTCTATTATCTCCAAAATGGAAGAGACATGTATGTGGACCAAGAATTAGATATTAATCGTTTAAGTGATTATGATGTCGATCACATTGTTCCACAAAGTTTCCTTAAAGACGATTCAATAGACAATAAGGTCTTAACGCGTTCTGATAAAAATCGTGGTAAATCGGATAACGTTCCAAGTGAAGAAGTAGTCAAAAAGATGAAAAACTATTGGAGACAACTTCTAAACGCCAAGTTAATCACTCAACGTAAGTTTGATAATTTAACGAAAGCTGAACGTGGAGGTTTGAGTGAACTTGATAAAGCTGGTTTTATCAAACGCCAATTGGTTGAAACTCGCGCGATCACTAAGCATGTGGCACAAATTTTGGATAGTCGCATGAATACTAAATACGATGAAAATGATAAACTTATTCGAGAGGTTAAAGTGATTACCTTAAAATCTAAATTAGTTTCTGACTTCCGAAAAGATTTCCAATTCTATAAAGTACGTGAGATTAACAATTACCATCATGCCCATGATGCGTATCTAAATGCCGTCGTTGGAACTGCTTTGAGATATCGAATTCGCGTTAATACGACTCACTATAGGGAGACCACAACGGTTTCCCTCTAGACACTCGAGTAACTAGTTAACCCCTTGGGGCCTCTAAACGGGTCTTGAGGGGTTTTTTGCTGAAAGGAGGAACTATATGCGCTCATACGATATGAACGTTGAGACTGCCGCTGAGTTATCAGCTGTGAACGACATTCTGGCGTCTATCGGTGAACCTC

>CE3-1 Part 1

GTAGACTCGGTTCGCACAGATGCCAACTATCGTCGTCAAGTCGAACAGAAAGTAATCGATTCGAACTTCTAACTAGATCTGTGCTCAAAGAGGAATCTATCAAGGGCGACACGCGAATTCGATATCGCGGCCGCGATATCAAGCTTATGTAGGTGACGGTCTCGAAGCCGCGGTGCGGGTGCCAGGGCGTGCCCTTGAGTTCTCTCAGTTGGGGGCGTAGGGTCGCCGACATGACACAAGGGGTTAAGCTTGATATCAAACCCTATTAACGCAAGTGGAGTAGATGCTAAAGCGATTCTTTCTGCACGATTGAGTAAATCAAGACGATTAGAAAATCTCATTGCTCAGCTCCCCGGTGAGAAGAAAAATGGCTTATTTGGGAATCTCATTGCTTTGTCATTGGGTTTGACCCCTAATTTTAAATCAAATTTTGATTTGGCAGAAGATGCTAAATTACAGCTTTCAAAAGATACTTACGATGATGATTTAGATAATTTATTGGCGCAAATTGGAGATCAATATGCTGATTTGTTTTTGGCAGCTAAGAATTTATCAGATGCTATTTTACTTTCAGATATCCTAAGAGTAAATACTGAAATAACTAAGGCTCCCCTATCAGCTTCAATGATTAAACGCTACGATGAACATCATCAAGACTTGACTCTTTTAAAAGCTTTAGTTCGACAACAACTTCCAGAAAAGTATAAAGAAATCTTTTTTGATCAATCAAAAAACGGATATGCAGGTTATATTGATGGGGGAGCTAGCCAAGAAGAATTTTATAAATTTATCAAACCAATTTTAGAAAAAATGGATGGTACTGAGGAATTATTGGTGAAACTAAATCGTGAAGATTTGCTGCGCAAGCAACGGACCTTTGACAACGGCTCTATTCCCATCAATTCACTTGGGTGAGCTGCTGCGATTTTTGGAAGAGAGAAGAATTTTATCCTTTTT

>CE3-1 Part 2

CGTCTATCTGATTTCCGCGCTTACGATGAACATCATCAAGACTTGACTCTTTTAAAAGCTTTAGTTCGACAACAACTTCCAGAAAAGTATAAAGAAATCTTTTTTGATCAATCAAAAAACGGATATGCAGGTTATATTGATGGGGGAGCTAGCCAAGAAGAATTTTATAAATTTATCAAACCAATTTTAGAAAAAATGGATGGTACTGAGGAATTATTGGTGAAACTAAATCGTGAAGATTTGCTGCGCAAGCAACGGACCTTTGACAACGGCTCTATTCCCCATCAAATTCACTTGGGTGAGCTGCATGCTATTTTGAGAAGACAAGAAGACTTTTATCCATTTTTAAAAGACAATCGTGAGAAGATTGAAAAAATCTTGACTTTTCGAATTCCTTATTATGTTGGTCCATTGGCGCGTGGCAATAGTCGTTTTGCATGGATGACTCGGAAGTCTGAAGAAACAATTACCCCATGGAATTTTGAAGAAGTTGTCGATAAAGGTGCTTCAGCTCAATCATTTATTGAACGCATGACAGCGTTTGATAAAAATCTTCCAAATGAAAAAGTACTACCAAAACATAGTTTGCTTTATGAGTATTTTACGGTTTATAACGAATTGACAAAGGTCAAATATGTTACTGAAGGAATGCGAAAACCAGCATTTCTTTCAGGTGAACAGAAGAAAGCCATTGTTGATTTACTCTTCAAAACAAATCGAAAAGTAACCGTTAAGCAATTAAAAGAAGATTATTTCAAAAAATAGAATGTTTTGATAGTGTTGAAATTTCAGGAGTTGAAGATAGATTTAATGCTTCATTAGGTACCTACCATGATTTGCTAAAATTATTAAAGATAAAGATTTTTGGATAATGAAGAAATGAGATATTTTAGAAGGAAATTGTTTAACTTGACTTATTGAGAAAGGGGAATATTGTTGGGGAAAGATTAAAACTATGCCACTCTTGAGGAAAAGGGGAGAACACTTAACTCCGTTAATGGGTGTGGGGGGGTTTCCCAAATGTTATGGGGGTTTGGGGAAAACATTGGAAAAAAATTAATTTTTAGAAACAGGGTTTGCACCCATTTTGGGGGCCCGCGGGGGTTTTGTTTTTAAGAAATTAAA

>CE3-1 Part 3

AAATTTTCCCAAGAGATTTTGCATAAATTTCTAGGGTTATAGGCAATCCCTAAGCCAATTATTCCCTGAGCAATCCGGATCCGACATGTCTTCAGATAGCGAGGATGCCATGTCTTCTATCTAGTACAGAAGTCGCTATTTAGCTCTTAACGACGAATCAGTCTAATTCATAGAATGTGAAGGTATCGGTGTAATCTCAGTACATGATGATAGATAGATGCATGATTCGGTACGTACCATGCTTTGCTACAAATTATTTACGATAAAGATTTTTTGGATAATGAAGAAAATGAAGATATCTTAGAGGATATTGTTCTAACATCGACCTTATTTGAAGATAGGGAGATGATTGAGGAAAGACTTAAAACATATGCTCACCTCTTTGATGATAAGGTGATGAAACAGCTTAAACGTCGCCGTTTATACTGGTTGGGGAGCGTTGTCTCGAAAATTGATTAATGGTATTAGGGATAAGCAATCTGGCAAAACAATATTAGATTTTTTGAAATCAGATGGTTTTGCCAATCGCAATTTTATGGCGCTGATCCATGATGATAGTTTGACATTTAAAGAAGACATTCAAAAAGCACAAGTGTCCGGACAAGGCGATAGTTTACATGAACATATTGCAAATTTAGCTGGTAGCCCTGCTATTAAAAAAGGTATTTTACAGACTGTAAAAGTTGTTGATGAATTGGTCAAAGTAATGGGGCGGCATAAGCCAGAAAATATCGTTATTGAAATGGCACGTGAAAATCAGACAACTCAAAAGGGCCAGAAAAATTCGCGAGAGCGTATGAAACGAATCGAAGAAGGTATCAAAGAATTAGGAAGTCAGATTCTTAAAGAGCATCCTGTTGAAAATACTCAATTGCAAAATGAAAAGCTCTATCTCTATTATCTCCAAAATGGAAGAGACATGTATGTGGACCAAGAATTAGATATTAATCGTTTAAGTGATTATGATGTCGATCACAGACACAATCAC

>CE3-1 Part 4

CTTTTAAAAAAAGGTTTTTTCCGGCCTGTAAAAGTTGTTGATGAATTGGTCAAAGTAATGGGGCGGCTTAAGCCAGAAAATTTCGTTTATGAAATGGCACGTGAAAATTCCGGCCAACCTCAAAAGGGCCAGAAAATTTCGCGAGAGCGTATGAAACGAATCGAAGGAAGGTATCAAAGAATTAGGAAGTCAGATTCTTAAAGAGCATCCTGTTGAAAATACTCAAATGCCAAATGAAAAGCTTCATCTCTCTATTATCTCCAAAATGGAAGAGACATGTATGTGGACCAAGAATTAGATATTAATCGTTTAAGTGATTATGATGTCGATCACATTGTTCCACAAAGTTTCCTTAAAGACGATTCAATAGACAATAAGGTCTTAACGCGTTCTGATAAAAATCGTGGTAAATCGGATAACGTTCCAAGTGAAGAAGTAGTCAAAAAGATGAAAAACTATTGGAGACAACTTCTAAACGCCAAGTTAATCACTCAACGTAAGTTTGATAATTTAACGAAAGCTGAACGTGGAGGTTTGAGTGAACTTGATAAAGCTGGTTTTATCAAACGCCAATTGGTTGAAACTCGCGCGATCACTAAGCATGTGGCACAAATTTTGGATAGTCGCATGAATACTAAATACGATGAAAATGATAAACTTATTCGAGAGGTTAAAGTGATTACCTTAAAATCTAAATTAGTTTCTGACTTCCGAAAAGATTTCCAATTCTATAAAGTACGTGAGATTAACAATTACCATCATGCCCATGATGCGTATCTAAATGCCGTCGTTGGAACTGCTTTGAGATATCGAATTCGCGTTAATACGACTCACTATAGGGAGACCACAACGGTTTCCCTCTAGACACTCGAGTAACTAGTTAACCCCTTGGGGCCTCTAAACGGGTCTTGAGGGGTTTTTTGCTGAAAGGAGGAACTATATGCGCTCATACGATATGAACGTTGAGACTGCCGCTAGTATCAGCTAGTC

>CE3-2 Part 1

GGTAGACCTCGGTTCGCAAGATGCCAACTATCGTCGTCAAGTCGAACAGAAAGTAATCGATTCGAACTTCTAACTAGATCTGTGCTCAAAGAGGAATCTATCAAGGGCGACACGCGAATTCGATATCGCGGCCGCGATATCAAGCTTATGTAGGTGACGGTCTCGAAGCCGCGGTGCGGGTGCCAGGGCGTGCCCTTGAGTTCTCTCAGTTGGGGGCGTAGGGTCGCCGACATGACACAAGGGGTTAAGCTTGATATCAAACCCTATTAACGCAAGTGGAGTAGATGCTAAAGCGATTCTTTCTGCACGATTGAGTAAATCAAGACGATTAGAAAATCTCATTGCTCAGCTCCCCGGTGAGAAGAAAAATGGCTTATTTGGGAATCTCATTGCTTTGTCATTGGGTTTGACCCCTAATTTTAAATCAAATTTTGATTTGGCAGAAGATGCTAAATTACAGCTTTCAAAAGATACTTACGATGATGATTTAGATAATTTATTGGCGCAAATTGGAGATCAATATGCTGATTTGTTTTTGGCAGCTAAGAATTTATCAGATGCTATTTTACTTTCAGATATCCTAAGAGTAAATACTGAAATAACTAAGGCTCCCCTATCAGCTTCAATGATTAAACGCTACGATGAACATCATCAAGACTTGACTCTTTTAAAAGCTTTAGTTCGACAACAACTTCCAGAAAAGTATAAAGAAAATCTTTTTTGATCAATCAAAAACGGATATGCAGGTTAATTGGTGGGGGGGGAGCTAGCCAAGAAGAATTTTATAAATTTATCAAACCAATTTTAGAAAAATGGAGGGACTGAAGGAATTTATGGGTGAAACTAATTCCGTGAAGATTTGCTGCGCAAGCAACGGACCTTTGACAACGGCTCTATTCCCATCAATTCACTTGGGTGACTGCAGGTATTTTGAAAAGACAAGAGAATTTATCCATTTTAAAACATCCGGGGAAGAATTGAAAAACTTGGCTTTCATTCCTTCTTATTTGGGCCTTGGGCGGGGGAAAACTTTTTTGGGGGAACCCAAAATTGAGAAAATTCCCCGGGGTTGTTTAAAGAAGTTGAAGGGGGGCCCCCCCTTTTTTAGGCGGGGCGCTGTTAAAATTCTTAAAGAGAAAACAAAATTTGTTTGTTT

>CE3-2 Part 2

CGCCAACTGGAAACCCCGCTACGATGAACATCATCAAGACTTGACTCTTTTAAAAGCTTTAGTTCGACAACAACTTCCAGAAAAGTATAAAGAAATCTTTTTTGATCAATCAAAAAACGGATATGCAGGTTATATTGATGGGGGAGCTAGCCAAGAAGAATTTTATAAATTTATCAAACCAATTTTAGAAAAAATGGATGGTACTGAGGAATTATTGGTGAAACTAAATCGTGAAGATTTGCTGCGCAAGCAACGGACCTTTGACAACGGCTCTATTCCCCATCAAATTCACTTGGGTGAGCTGCATGCTATTTTGAGAAGACAAGAAGACTTTTATCCATTTTTAAAAGACAATCGTGAGAAGATTGAAAAAATCTTGACTTTTCGAATTCCTTATTATGTTGGTCCATTGGCGCGTGGCAATAGTCGTTTTGCATGGATGACTCGGAAGTCTGAAGAAACAATTACCCCATGGAATTTTGAAGAAGTTGTCGATAAAGGTGCTTCAGCTCAATCATTTATTGAACGCATGACAGCGTTTGATAAAAATCTTCCAAATGAAAAAGTACTACCAAAGACATAGTTTGCTTTATGAGTATTTTACGGTTTATAACGAATTGACAAAGGTCAAATATGTTACTGAAGGAATGCGAAAACCAGCATTTCTTTCAGGTGAACAGAAGAAAGCCATTTGTTGATTTACTCTTCAAAACAAATCGAAAAGTAACCGTTAAGCAATTAAAAGAAGATTATTTCAAAAAATAGAATGTTTTGAAAGTGGTGAAATTTCAGGAGTTGAAGAATAGATTTAATGCTTCATTAGGTACCTACCATGATTTGCTAAAATTATAAAGATAAAGATTTTTTGGATAATGAAGAAAATGAGATATCTTAGAAGGGAATTGGTTTAACATTGACCTTATTGAGAAAAGGGGAAGGAGGTTTGGAGAAAGACTTAAAACTTATGCTCACCTCTTTGAAGAAAAGGTGATGAAACGCTTAAACGTCGCGTTTAACGGGGGTGGGGAGCTTTTCCCAAAATTGATAAGGGGTTTTAGGGATAAGCA

>CE3-2 Part 3

AAATTTCTTTTTTTCTTTTTTGGCGCCGGGCGGCAATTTTTTTGCGGAAGCTGGGGGGAAAAACACCCCCCCCCCGGGGTTTGAAATTGGAAAAGGGTTCCCCCCACCTTTTTTGACCCCGGCCGGGTTTTAAAAATTCTCCAAAAAAAAAAGTCTCCCAACACATTTTTGCTTTAGAGTATTTACGGTTTTAACGAATTGCCAAAGGTCAAATATGTTACTGAAGGAATGCGAAACCAGCATTTTCTTCAGGGAACAGAAGAAAGCCATTGTTGATTTCTCTTCAAAACAAATCGAAAAGTAACCGTTAAGCAATTAAAAGAAGATTATTTCAAAAAAATAGAATGTTTTGATAGTGTTGAAATTTCAGGAGTTGAAGATAGATTTAATGCTTCATTAGGTACCTACCATGATTTGCTAAAAATTATTAAAGATAAAGATTTTTTGGATAATGAAGAAAATGAAGATATCTTAGAGGATATTGTTTTAACATTGACCTTATTTGAAGATAGGGAGATGATTGAGGAAAGACTTAAAACATATGCTCACCTCTTTGATGATAAGGTGATGAAACAGCTTAAACGTCGCCGTTATACTGGTTGGGGAGCGTTGTCTCGAAAATTGATTAATGGTATTAGGGATAAGCAATCTGGCAAAACAATATTAGATTTTTTGAAATCAGATGGTTTTGCCAATCGCAATTTTATGGCGCTGATCCATGATGATAGTTTGACATTTAAAGAAGACATTCAAAAAGCACAAGTGTCCGGACAAGGCGATAGTTTACATGAACATATTGCAAATTTAGCTGGTAGCCCTGCTATTAAAAAAGGTATTTTACAGACTGTAAAAGTTGTTGATGAATTGGTCAAAGTAATGGGGCGGCATAAGCCAGAAAATATCGTTATTGAAATGGCACGTGAAAATCAGACAACTCAAAAGGGCCAGAAAAATTCGCGAGAGCGTATGAAACGAATCGAAGAAGGTATCAAAGAATTAGGAAGTCAGATTCTTAAAGAGCATCCTGTTGAAAATACTCAATTGCAAAATGAAAAGCTCTATCTCTATTATCTCCAAAATGGAAGAGACATGTATGTGGACCAAGAATTAGATATTAATCGTTTAAGTGATGAATTAATGTCGAAGTACATTAACACTCCATTCC

>CE3-2 Part 4

AGTAATGGCGCGCATAACCCAGAAAATTTCGTTATTGAAATGGCACGTGAAAATTCAGACAACTCAAAAGGGCCAGAAAATTCGCGAGAGCGTATGAAACGAATCGAAGAAGGTATCAAAGAATTAGGAAGTCAGATTCTTAAAGAGCATCCTGTTGAAAATACTCAATTGCAAAATGAAAAGCTCTATCTCTATTATCTCCAAAATGGAAGAGACATGTATGTGGACCAAGAATTAGATATTAATCGTTTAAGTGATTATGATGTCGATCACATTGTTCCACAAAGTTTCCTTAAAGACGATTCAATAGACAATAAGGTCTTAACGCGTTCTGATAAAAATCGTGGTAAATCGGATAACGTTCCAAGTGAAGAAGTAGTCAAAAAGATGAAAAACTATTGGAGACAACTTCTAAACGCCAAGTTAATCACTCAACGTAAGTTTGATAATTTAACGAAAGCTGAACGTGGAGGTTTGAGTGAACTTGATAAAGCTGGTTTTATCAAACGCCAATTGGTTGAAACTCGCGCGATCACTAAGCATGTGGCACAAATTTTGGATAGTCGCATGAATACTAAATACGATGAAAATGATAAACTTATTCGAGAGGTTAAAGTGATTACCTTAAAATCTAAATTAGTTTCTGACTTCCGAAAAGATTTCCAATTCTATAAAGTACGTGAGATTAACAATTACCATCATGCCCATGATGCGTATCTAAATGCCGTCGTTGGAACTGCTTTGAGATATCGAATTCGCGTTAATACGACTCACTATAGGGAGACCACAACGGTTTCCCTCTAGACACTCGAGTAACTAGTTAACCCCTTGGGGCCTCTAAACGGGTCTTGAGGGGTTTTTTGCTGAAAGGAGGAACTATATGCGCTCATACGATATGAACGTGAGACTGCCGCTAGTATCAGCGAGTC

Expected Sequence of CE4-Int T7 genome amplified by InsertSeq-F1 and InsertSeq-R1

>Expected product

GGACCGTAGCGAGATGATTAAAGCTATGAGTGACCCTCGGTATCGCACAGATGCCAACTATCGTCGTCAAGTCGAACAGAAAGTAATCGATTCGAACTTCTAACTAGATCTGTGCTCAAAGAGGAATCTATCAAGGGCGACACGCGAATTCGATATCAAGCTTATGTAGGTGACGGTCTCGAAGCCGCGGTGCGGGTGCCAGGGCGTGCCCTTGAGTTCTCTCAGTTGGGGGCGTAGGGTCGCCGACATGACACAAGGGGTTAAGCTTGATATCCAAATGAGATGGCGAAAGTAGATGATAGTTTCTTTCATCGACTTGAAGAGTCTTTTTTGGTGGAAGAAGACAAGAAGCATGAACGTCATCCTATTTTTGGAAATATAGTAGATGAAGTTGCTTATCATGAGAAATATCCAACTATCTATCATCTGCGAAAAAAATTGGTAGATTCTACTGATAAAGCGGATTTGCGCTTAATCTATTTGGCCTTAGCGCATATGATTAAGTTTCGTGGTCATTTTTTGATTGAGGGAGATTTAAATCCTGATAATAGTGATGTGGACAAACTATTTATCCAGTTGGTACAAACCTACAATCAATTATTTGAAGAAAACCCTATTAACGCAAGTGGAGTAGATGCTAAAGCGATTCTTTCTGCACGATTGAGTAAATCAAGACGATTAGAAAATCTCATTGCTCAGCTCCCCGGTGAGAAGAAAAATGGCTTATTTGGGAATCTCATTGCTTTGTCATTGGGTTTGACCCCTAATTTTAAATCAAATTTTGATTTGGCAGAAGATGCTAAATTACAGCTTTCAAAAGATACTTACGATGATGATTTAGATAATTTATTGGCGCAAATTGGAGATCAATATGCTGATTTGTTTTTGGCAGCTAAGAATTTATCAGATGCTATTTTACTTTCAGATATCCTAAGAGTAAATACTGAAATAACTAAGGCTCCCCTATCAGCTTCAATGATTAAACGCTACGATGAACATCATCAAGACTTGACTCTTTTAAAAGCTTTAGTTCGACAACAACTTCCAGAAAAGTATAAAGAAATCTTTTTTGATCAATCAAAAAACGGATATGCAGGTTATATTGATGGGGGAGCTAGCCAAGAAGAATTTTATAAATTTATCAAACCAATTTTAGAAAAAATGGATGGTACTGAGGAATTATTGGTGAAACTAAATCGTGAAGATTTGCTGCGCAAGCAACGGACCTTTGACAACGGCTCTATTCCCCATCAAATTCACTTGGGTGAGCTGCATGCTATTTTGAGAAGACAAGAAGACTTTTATCCATTTTTAAAAGACAATCGTGAGAAGATTGAAAAAATCTTGACTTTTCGAATTCCTTATTATGTTGGTCCATTGGCGCGTGGCAATAGTCGTTTTGCATGGATGACTCGGAAGTCTGAAGAAACAATTACCCCATGGAATTTTGAAGAAGTTGTCGATAAAGGTGCTTCAGCTCAATCATTTATTGAACGCATGACAGCGTTTGATAAAAATCTTCCAAATGAAAAAGTACTACCAAAACATAGTTTGCTTTATGAGTATTTTACGGTTTATAACGAATTGACAAAGGTCAAATATGTTACTGAAGGAATGCGAAAACCAGCATTTCTTTCAGGTGAACAGAAGAAAGCCATTGTTGATTTACTCTTCAAAACAAATCGAAAAGTAACCGTTAAGCAATTAAAAGAAGATTATTTCAAAAAAATAGAATGTTTTGATAGTGTTGAAATTTCAGGAGTTGAAGATAGATTTAATGCTTCATTAGGTACCTACCATGATTTGCTAAAAATTATTAAAGATAAAGATTTTTTGGATAATGAAGAAAATGAAGATATCTTAGAGGATATTGTTTTAACATTGACCTTATTTGAAGATAGGGAGATGATTGAGGAAAGACTTAAAACATATGCTCACCTCTTTGATGATAAGGTGATGAAACAGCTTAAACGTCGCCGTTATACTGGTTGGGGAGCGTTGTCTCGAAAATTGATTAATGGTATTAGGGATAAGCAATCTGGCAAAACAATATTAGATTTTTTGAAATCAGATGGTTTTGCCAATCGCAATTTTATGGCGCTGATCCATGATGATAGTTTGACATTTAAAGAAGACATTCAAAAAGCACAAGTGTCCGGACAAGGCGATAGTTTACATGAACATATTGCAAATTTAGCTGGTAGCCCTGCTATTAAAAAAGGTATTTTACAGACTGTAAAAGTTGTTGATGAATTGGTCAAAGTAATGGGGCGGCATAAGCCAGAAAATATCGTTATTGAAATGGCACGTGAAAATCAGACAACTCAAAAGGGCCAGAAAAATTCGCGAGAGCGTATGAAACGAATCGAAGAAGGTATCAAAGAATTAGGAAGTCAGATTCTTAAAGAGCATCCTGTTGAAAATACTCAATTGCAAAATGAAAAGCTCTATCTCTATTATCTCCAAAATGGAAGAGACATGTATGTGGACCAAGAATTAGATATTAATCGTTTAAGTGATTATGATGTCGATCACATTGTTCCACAAAGTTTCCTTAAAGACGATTCAATAGACAATAAGGTCTTAACGCGTTCTGATAAAAATCGTGGTAAATCGGATAACGTTCCAAGTGAAGAAGTAGTCAAAAAGATGAAAAACTATTGGAGACAACTTCTAAACGCCAAGTTAATCACTCAACGTAAGTTTGATAATTTAACGAAAGCTGAACGTGGAGGTTTGAGTGAACTTGATAAAGCTGGTTTTATCAAACGCCAATTGGTTGAAACTCGCGCGATCACTAAGCATGTGGCACAAATTTTGGATAGTCGCATGAATACTAAATACGATGAAAATGATAAACTTATTCGAGAGGTTAAAGTGATTACCTTAAAATCTAAATTAGTTTCTGACTTCCGAAAAGATTTCCAATTCTATAAAGTACGTGAGATTAACAATTACCATCATGCCCATGATGCGTATCTAAATGCCGTCGTTGGAACTGCTTTGATTAAGAAATATCCAAAACTTGAATCGGAGTTTGTCTATGGTGATTATAAAGTTTATGATGTTCGTAAAATGATTGCTAAGTCTGAGCAAGAAATAGGCAAAGCAACCGCAAAATATTTCTTTTACTCTAATATCATGAACTTCTTCAAAACAGAAATTACACTTGCAAATGGAGAGATTCGCAAACGCCCTCTAATCGAAACTAATGGGGAAACTGGAGAAATTGTCTGGGATAAAGGGCGAGATTTTGCCACAGTGCGCAAAGTATTGTCCATGCCCCAAGTCAATATTGTCAAGAAAACAGAAGTACAGACAGGCGGATTCTCGATATCGAATTCGCGTTAATACGACTCACTATAGGGAGACCACAACGGTTTCCCTCTAGACACTCGAGTAACTAGTTAACCCCTTGGGGCCTCTAAACGGGTCTTGAGGGGTTTTTTGCTGAAAGGAGGAACTATATGCGCTCATACGATATGAACGTTGAGACTGCCGCTGAGTTATCAGCTGTGAACGACATTCTGGCGTCTATCGGTGAACCTC

>CE4-1

CAAGATTAAAGCTATGAGTGACCCTCGGTATCGCACAGATGCCAACTATCGTCGTCAAGTCGAACAGAAAGTAATCGATTCGAACTTCTAACTAGATCTGTGCTCAAAGAGGAATCTATCAAGGGCGACACGCGAATTCGATATCGCGGCCGCGATATCAAGCTTATGTAGGTGACGGTCTCGAAGCCGCGGTGCGGGTGCCAGGGCGTGCCCTTGGGCTCCCCGGGCGCGTACTCCACCTCACCCATCTGGTCCATCATGATGAACGGGTCGGCTAGCCGAAATTAATACGACTCACTATAGGGAGACCACAACGGTTTCCCTCTAGACACTCGAGTAACTAGTTAACCCCTTGGGGCCTCTAAACGGGTCTTGAGGGGTTTTTTGCTGAAAGGAGGAACTATATGCGCTCATACGATATGAACGTTGAGACTGCCGCTGAGTTATCAGCTGTGAACGACATTCTGGCGCAG

>CE4-2

CAAGATTAAAGCTATGAGTGACCCTCGGTATCGCACAGATGCCAACTATCGTCGTCAAGTCGAACAGAAAGTAATCGATTCGAACTTCTAACTAGATCTGTGCTCAAAGAGGAATCTATCAAGGGCGACACGCGAATTCGATATCGCGGCCGCGATATCAAGCTTATGTAGGTGACGGTCTCGAAGCCGCGGTGCGGGTGCCAGGGCGTGCCCTTGGGCTCCCCGGGCGCGTACTCCACCTCACCCATCTGGTCCATCATGATGAACGGGTCGGCTAGCCGAAATTAATACGACTCACTATAGGGAGACCACAACGGTTTCCCTCTAGACACTCGAGTAACTAGTTAACCCCTTGGGGCCTCTAAACGGGTCTTGAGGGGTTTTTTGCTGAAAGGAGGAACTATATGCGCTCATACGATATGAACGTTGAGACTGCCGCTGAGTTATCAGCTGTGAACGACATTCTGGCGTCA
