# Supplementary material for: A novel approach for T7 bacteriophage genome integration of exogenous DNA
Source: J Biol Eng. 2020 Jan 16;14:2. doi: 10.1186/s13036-019-0224-x (PMC6966851; doi:10.1186/s13036-019-0224-x)
Supplement: Supplementary file 3 — Additional file 3. Sequencing results of engineered T7 phage and the positive recombinants harboring the exogenous DNA were documented in three files respectively, these files comprised the crude statistics as well as the processed sequences in docx format. [file 13036_2019_224_MOESM3_ESM.zip › Additional file 3/pMCBK-Int T7 sequencing/pMCBK-Int T7 sequencing.docx]

>Expected U region

CCTTGAGACGCACAACCCTGAGGCTGCACAGTCGCTGGATAATGCGTTGACCAATCGTGACTTAGCGACCGTTAAGGCTATCATCAACTTGGCTGGTGAGTCTCGCGCTAAGGCGTTCGGTCGTAAGCCAACTCGTAGTGTGACTAATCGTGCTATTCCGGCTAAACCTCAGGCTACCAAGCGTGAAGGCTTTGCGGACCGTAGCGAGATGATTAAAGCTATGAGTGACCCTCGGTATCGCACAGATGCCAACTATCGTCGTCAAGTCGAACAGAAAGTAATCGATTCGAACTTCTAACTAGATCTGTGCTCAAAGAGGAATCTATCAAGGGCGACACGCGAATTCGATATCAAGCTTATGTAGGTGACGGTCTCGAAGCCGCGGTGCGGGTGCCAGGGCGTGCCCTTGAGTTCTCTCAGTTGGGGGCGTAGGGTCGCCGACATGACACAAGGGGTTAAGCTTGATATCGAATTCGCGTTAATACGACTCACTATAGGGAGACCACAACGGTTTCCCTCTAGACACTCGAGTAACTAGTTAACCCCTTGGGGCCTCTAAACGGGTCTTGAGGGGTTTTTTGCTGAAAGGAGGAACTATATGCGCTCATACGATATGAACGTTGAGACTGCCGCTGAGTTATCAGCTGTGAACGACATTCTGGCGTCTATCGGTGAACCTCCGGTATCAACGCTGGAAGGTGACGCTAACGCAGATGCAGCGAACGCTCGGCGTATTCTCAACAAGATTAACCGACAGATTCAATCTCGTGGATGGACGTTCAACATTGAGGAAGGCATAACGCTACTACCTGATGTTTACTCCAACCTGATTGTATACAGTGACGACTATTTATCCCTAATGTCTACTTCCGGTCAATCCATCTACGTTAACCGAGGTGGCTATGTGTATGACCGAACGAGTCAATCAGACCGCTTTGACTCTGGTATTACTGTGAACATTATTCGTCTCCGCGACTACGATGAGATGCCTGAGTGCTTCCGTTACTGGATTGTCACCAAGGCTTCCCGTCAGTTCAACAACCGATTCTTTGGGGCACCGGAAGTAGAGGGTGTACTCCAAGAAGAGGAAGATGAGGCTAGACGTCTCTGCATGGAGTATGAGATGGACTACGGTGGGTACAATATGCTGGATGGAGATGCGTTCACTTCTGGTCTACTGACTCGCTAAGTTACTCGTGTGCGTCCTTAAGCGGCCGCCTGCAGTCAATACTGACGATGGTCATAGCTGTTTCCTGTCCATAGCAGAAAGTCAAAAGCCTCCGACCGGAGGCTTTTGACTTGATCGGCACGTAAGAGGTTCCAACTTTCACCATAATGAAATAAGATCACTACCGGGCGTATTTTTTGAGTTATCGAGATTTTCAGGAGCTAAGGAAGCTAAAATGAGCCATATTCAACGGGAAACGTCGAGGCCGCGATTAAATTCCAACATGGATGCTGATTTATATGGGTATAAATGGGCTCGCGATAATGTCGGGCAATCAGGTGCGACAATCTATCGCTTGTATGGGAAGCCCGATGCGCCAGAGTTGTTTCTGAAACATGGCAAAGGTAGCGTTGCCAATGAT

>Expected D region

TTTTCAGAGCAAGAGATTACGCGCAGACCAAAACGATCTCAAGAAGATCCTTTGATTTTCTACCGAAGAAAGGCCCACCCGTGAAGGTGAGCCAGTGAGTTGATTGTGTAAAACGACGGCCAGTGAATTCCTCGCTGCAGTCCTGAAGCTTCCAGGTCAGAAGCGGTTTTCGGGAGTAGTGCCCCAACTGGGGTAACCTTTGGGCTCCCCGGGCGCGTACTCCACCTCACCCATCTGGTCCATCATGATGAACGGGTCGGCTAGCCGAAATTAATACGACTCACTATAGGGAGACCACAACGGTTTCCCTCTAGACACTCGAGTAACTAGTTAACCCCTTGGGGCCTCTAAACGGGTCTTGAGGGGTTTTTTGCTGAAAGGAGGAACTGAGGCGAGTGTTACTTCAACCTGGTCTACTGACTCGCTAACATTAATAAATAAGGAGGCTCTAATGGCACTCATTAGCCAATCAATCAAGAACTTGAAGGGTGGTATCAGCCAACAGCCTGACATCCTTC

>U1-F

CATCGCTGGGATATGCGTTGACCAATCGTGACTTAGCGACCGTTAAGGCTATCATCAACTTGGCTGGTGAGTCTCGCGCTAAGGCGTTCGGTCGTAAGCCAACTCGTAGTGTGACTAATCGTGCTATTCCGGCTAAACCTCAGGCTACCAAGCGTGAAGGCTTTGCGGACCGTAGCGAGATGATTAAAGCTATGAGTGACCCTCGGTATCGCACAGATGCCAACTATCGTCGTCAAGTCGAACAGAAAGTAATCGATTCGAACTTCTAACTAGATCTGTGCTCAAAGAGGAATCTATCAAGGGCGACACGCGAATTCGATATCGCGGCCGCGATATCAAGCTTATGTAGGTGACGGTCTCGAAGCCGCGGTGCGGGTGCCAGGGCGTGCCCTTGAGTTCTCTCAGTTGGGGGCGTAGGGTCGCCGACATGACACAAGGGGTTAAGCTTGATATCGAATTCGCGTTAATACGACTCACTATAGGGAGACCACAACGGTTTCCCTCTAGACACTCGAGTAACTAGTTAACCCCTTGGGGCCTCTAAACGGGTCTTGAGGGGTTTTTTGCTGAAAGGAGGAACTATATGCGCTCATACGATATGAACGTTGAGACTGCCGCTGAGTTATCAGCTGTGAACGACATTCTGGCGTCTATCGGTGAACCTCCGGTATCAACGCTGGAAGGTGACGCTAACGCAGATGCAGCGAACGCTCGGCGTATTCTCAACAAGATTAACCGACAGATTCAATCTCGTGGATGGACGTTCAACATTGAGGAAGGCATAACGCTACTACCTGATGTTTACTCCAACCTGATTGTATACAGTGACGACTATTTATCCCTAATGTCTACTTCCGGTCAATCCATCTACGTTAACCGAGGTGGCTATGTGTATGACCGAACGAGTCAATCAGACCGCTTTGACTCTGGTATTACTGTGAACATTATTCGTCTCCGCGACTACAATG

>U2-R

TCGATATGAACGTTGAGACTGCCGCTGAGTTATCAGCTGTGAACGACATTCTGGCGTCTATCGGTGAACCTCCGGTATCAACGCTGGAAGGTGACGCTAACGCAGATGCAGCGAACGCTCGGCGTATTCTCAACAAGATTAACCGACAGATTCAATCTCGTGGATGGACGTTCAACATTGAGGAAGGCATAACGCTACTACCTGATGTTTACTCCAACCTGATTGTATACAGTGACGACTATTTATCCCTAATGTCTACTTCCGGTCAATCCATCTACGTTAACCGAGGTGGCTATGTGTATGACCGAACGAGTCAATCAGACCGCTTTGACTCTGGTATTACTGTGAACATTATTCGTCTCCGCGACTACGATGAGATGCCTGAGTGCTTCCGTTACTGGATTGTCACCAAGGCTTCCCGTCAGTTCAACAACCGATTCTTTGGGGCACCGGAAGTAGAGGGTGTACTCCAAGAAGAGGAAGATGAGGCTAGACGTCTCTGCATGGAGTATGAGATGGACTACGGTGGGTACAATATGCTGGATGGAGATGCGTTCACTTCTGGTCTACTGACTCGCTAAGTTACTCGTGTGCGTCCTTAAGCGGCCGCCTGCAGTCAATACTGACGATGGTCATAGCTGTTTCCTGTCCATAGCAGAAAGTCAAAAGCCTCCGACCGGAGGCTTTTGACTTGATCGGCACGTAAGAGGTTCCAACTTTCACCATAATGAAATAAGATCACTACCGGGCGTATTTTTTGAGTTATCGAGATTTTCAGGAGCTAAGGAAGCTAAAATGAGCCATATTCAACGGGAAACGTCTTGCTCTAGGCCGCGATTAAATTCCAACATGGATGCTGATTTATATGGGTATAAATGGGCTCGCGATAATGTCGGGCAATCAGGTGCGACAATCTATCGATTGTATGGGAAGCCCGAGCGCCAGAGTTTT

>U1

CATCGCTGGGATATGCGTTGACCAATCGTGACTTAGCGACCGTTAAGGCTATCATCAACTTGGCTGGTGAGTCTCGCGCTAAGGCGTTCGGTCGTAAGCCAACTCGTAGTGTGACTAATCGTGCTATTCCGGCTAAACCTCAGGCTACCAAGCGTGAAGGCTTTGCGGACCGTAGCGAGATGATTAAAGCTATGAGTGACCCTCGGTATCGCACAGATGCCAACTATCGTCGTCAAGTCGAACAGAAAGTAATCGATTCGAACTTCTAACTAGATCTGTGCTCAAAGAGGAATCTATCAAGGGCGACACGCGAATTCGATATCGCGGCCGCGATATCAAGCTTATGTAGGTGACGGT**CTCGAAGCCGCGGTGCGGGTGCCAGGGCGTGCCCTTGAGTTCTCTCAGTTGGGGGCGTAGGGTCGCCGACATGACACAAGGGGTT**AAGCTTGATATCGAATTCGCGTTAATACGACTCACTATAGGGAGACCACAACGGTTTCCCTCTAGACACTCGAGTAACTAGTTAACCCCTTGGGGCCTCTAAACGGGTCTTGAGGGGTTTTTTGCTGAAAGGAGGAACTATATGCGCTCATACGATATGAACGTTGAGACTGCCGCTGAGTTATCAGCTGTGAACGACATTCTGGCGTCTATCGGTGAACCTCCGGTATCAACGCTGGAAGGTGACGCTAACGCAGATGCAGCGAACGCTCGGCGTATTCTCAACAAGATTAACCGACAGATTCAATCTCGTGGATGGACGTTCAACATTGAGGAAGGCATAACGCTACTACCTGATGTTTACTCCAACCTGATTGTATACAGTGACGACTATTTATCCCTAATGTCTACTTCCGGTCAATCCATCTACGTTAACCGAGGTGGCTATGTGTATGACCGAACGAGTCAATCAGACCGCTTTGACTCTGGTATTACTGTGAACATTATTCGTCTCCGCGACTACGATGAGATGCCTGAGTGCTTCCGTTACTGGATTGTCACCAAGGCTTCCCGTCAGTTCAACAACCGATTCTTTGGGGCACCGGAAGTAGAGGGTGTACTCCAAGAAGAGGAAGATGAGGCTAGACGTCTCTGCATGGAGTATGAGATGGACTACGGTGGGTACAATATGCTGGATGGAGATGCGTTCACTTCTGGTCTACTGACTCGCTAAGTTACTCGTGTGCGTCCTTAAGCGGCCGCCTGCAGTCAATACTGACGATGGTCATAGCTGTTTCCTGTCCATAGCAGAAAGTCAAAAGCCTCCGACCGGAGGCTTTTGACTTGATCGGCACGTAAGAGGTTCCAACTTTCACCATAATGAAATAAGATCACTACCGGGCGTATTTTTTGAGTTATCGAGATTTTCAGGAGCTAAGGAAGCTAAAATGAGCCATATTCAACGGGAAACGTCTTGCTCTAGGCCGCGATTAAATTCCAACATGGATGCTGATTTATATGGGTATAAATGGGCTCGCGATAATGTCGGGCAATCAGGTGCGACAATCTATCGATTGTATGGGAAGCCCGAGCGCCAGAGTTTT

>U2-F

CTTCGCCTGGGATATGCGTTGACCAATCGTGACTTAGCGACCGTTAAGGCTATCATCAACTTGGCTGGTGAGTCTCGCGCTAAGGCGTTCGGTCGTAAGCCAACTCGTAGTGTGACTAATCGTGCTATTCCGGCTAAACCTCAGGCTACCAAGCGTGAAGGCTTTGCGGACCGTAGCGAGATGATTAAAGCTATGAGTGACCCTCGGTATCGCACAGATGCCAACTATCGTCGTCAAGTCGAACAGAAAGTAATCGATTCGAACTTCTAACTAGATCTGTGCTCAAAGAGGAATCTATCAAGGGCGACACGCGAATTCGATATCGCGGCCGCGATATCAAGCTTATGTAGGTGACGGT**CTCGAAGCCGCGGTGCGGGTGCCAGGGCGTGCCCTTGAGTTCTCTCAGTTGGGGGCGTAGGGTCGCCGACATGACACAAGGGGTT**AAGCTTGATATCGAATTCGCGTTAATACGACTCACTATAGGGAGACCACAACGGTTTCCCTCTAGACACTCGAGTAACTAGTTAACCCCTTGGGGCCTCTAAACGGGTCTTGAGGGGTTTTTTGCTGAAAGGAGGAACTATATGCGCTCATACGATATGAACGTTGAGACTGCCGCTGAGTTATCAGCTGTGAACGACATTCTGGCGTCTATCGGTGAACCTCCGGTATCAACGCTGGAAGGTGACGCTAACGCAGATGCAGCGAACGCTCGGCGTATTCTCAACAAGATTAACCGACAGATTCAATCTCGTGGATGGACGTTCAACATTGAGGAAGGCATAACGCTACTACCTGATGTTTACTCCAACCTGATTGTATACAGTGACGACTATTTATCCCTAATGTCTACTTCCGGTCAATCCATCTACGTTAACCGAGGTGGCTATGTGTATGACCGAACGAGTCAATCAGACCGCTTTGACTCTGGTATTACTGTGAA

>U2-R

TTGAACGTTGAGACTGCCGCTGAGTTATCAGCTGTGAACGACATTCTGGCGTCTATCGGTGAACCTCCGGTATCAACGCTGGAAGGTGACGCTAACGCAGATGCAGCGAACGCTCGGCGTATTCTCAACAAGATTAACCGACAGATTCAATCTCGTGGATGGACGTTCAACATTGAGGAAGGCATAACGCTACTACCTGATGTTTACTCCAACCTGATTGTATACAGTGACGACTATTTATCCCTAATGTCTACTTCCGGTCAATCCATCTACGTTAACCGAGGTGGCTATGTGTATGACCGAACGAGTCAATCAGACCGCTTTGACTCTGGTATTACTGTGAACATTATTCGTCTCCGCGACTACGATGAGATGCCTGAGTGCTTCCGTTACTGGATTGTCACCAAGGCTTCCCGTCAGTTCAACAACCGATTCTTTGGGGCACCGGAAGTAGAGGGTGTACTCCAAGAAGAGGAAGATGAGGCTAGACGTCTCTGCATGGAGTATGAGATGGACTACGGTGGGTACAATATGCTGGATGGAGATGCGTTCACTTCTGGTCTACTGACTCGCTAAGTTACTCGTGTGCGTCCTTAAGCGGCCGCCTGCAGTCAATACTGACGATGGTCATAGCTGTTTCCTGTCCATAGCAGAAAGTCAAAAGCCTCCGACCGGAGGCTTTTGACTTGATCGGCACGTAAGAGGTTCCAACTTTCACCATAATGAAATAAGATCACTACCGGGCGTATTTTTTGAGTTATCGAGATTTTCAGGAGCTAAGGAAGCTAAAATGAGCCATATTCAACGGGAAACGTCTTGCTCTAGGCCGCGATTAAATTCCAACATGGATGCTGATTTATATGGGTATAAATGGGCTCGCGATAATGTCGGGCAATCAGGTGCGACAATCTATCGATTGTATGGGAAGCCCGAGCGCCCCAGATTTT

>U2

CTTCGCCTGGGATATGCGTTGACCAATCGTGACTTAGCGACCGTTAAGGCTATCATCAACTTGGCTGGTGAGTCTCGCGCTAAGGCGTTCGGTCGTAAGCCAACTCGTAGTGTGACTAATCGTGCTATTCCGGCTAAACCTCAGGCTACCAAGCGTGAAGGCTTTGCGGACCGTAGCGAGATGATTAAAGCTATGAGTGACCCTCGGTATCGCACAGATGCCAACTATCGTCGTCAAGTCGAACAGAAAGTAATCGATTCGAACTTCTAACTAGATCTGTGCTCAAAGAGGAATCTATCAAGGGCGACACGCGAATTCGATATCGCGGCCGCGATATCAAGCTTATGTAGGTGACGGTCTCGAAGCCGCGGTGCGGGTGCCAGGGCGTGCCCTTGAGTTCTCTCAGTTGGGGGCGTAGGGTCGCCGACATGACACAAGGGGTTAAGCTTGATATCGAATTCGCGTTAATACGACTCACTATAGGGAGACCACAACGGTTTCCCTCTAGACACTCGAGTAACTAGTTAACCCCTTGGGGCCTCTAAACGGGTCTTGAGGGGTTTTTTGCTGAAAGGAGGAACTATATGCGCTCATACGATATGAACGTTGAGACTGCCGCTGAGTTATCAGCTGTGAACGACATTCTGGCGTCTATCGGTGAACCTCCGGTATCAACGCTGGAAGGTGACGCTAACGCAGATGCAGCGAACGCTCGGCGTATTCTCAACAAGATTAACCGACAGATTCAATCTCGTGGATGGACGTTCAACATTGAGGAAGGCATAACGCTACTACCTGATGTTTACTCCAACCTGATTGTATACAGTGACGACTATTTATCCCTAATGTCTACTTCCGGTCAATCCATCTACGTTAACCGAGGTGGCTATGTGTATGACCGAACGAGTCAATCAGACCGCTTTGACTCTGGTATTACTGTGAACATTATTCGTCTCCGCGACTACGATGAGATGCCTGAGTGCTTCCGTTACTGGATTGTCACCAAGGCTTCCCGTCAGTTCAACAACCGATTCTTTGGGGCACCGGAAGTAGAGGGTGTACTCCAAGAAGAGGAAGATGAGGCTAGACGTCTCTGCATGGAGTATGAGATGGACTACGGTGGGTACAATATGCTGGATGGAGATGCGTTCACTTCTGGTCTACTGACTCGCTAAGTTACTCGTGTGCGTCCTTAAGCGGCCGCCTGCAGTCAATACTGACGATGGTCATAGCTGTTTCCTGTCCATAGCAGAAAGTCAAAAGCCTCCGACCGGAGGCTTTTGACTTGATCGGCACGTAAGAGGTTCCAACTTTCACCATAATGAAATAAGATCACTACCGGGCGTATTTTTTGAGTTATCGAGATTTTCAGGAGCTAAGGAAGCTAAAATGAGCCATATTCAACGGGAAACGTCTTGCTCTAGGCCGCGATTAAATTCCAACATGGATGCTGATTTATATGGGTATAAATGGGCTCGCGATAATGTCGGGCAATCAGGTGCGACAATCTATCGATTGTATGGGAAGCCCGAGCGCCCCAGATTTT

>D1-F

AGAATGATCCTTTGATTTTCTACCGAAGAAAGGCCCACCCGTGAAGGTGAGCCAGTGAGTTGATTGTGTAAAACGACGGCCAGTGAATTCCTCGCTGCAGTCCTGAAGCTTCCAGGTCAGAAGCGGTTTTCGGGAGTAGTGCCCCAACTGGGGTAACCTTTGGGCTCCCCGGGCGCGTACTCCACCTCACCCATCTGGTCCATCATGATGAACGGGTCGGCTAGCCGAAATTAATACGACTCACTATAGGGAGACCACAACGGTTTCCCTCTAGACACTCGAGTAACTAGTTAACCCCTTGGGGCCTCTAAACGGGTCTTGAGGGGTTTTTTGCTGAAAGGAGGAACTGAGGCGAGTGTTACTTCAACCTGGTCTACTGACTCGCTAACATTAATAAATAAGGAGGCTCTAATGGCACTCATTAGCCAATCAATCAAGAACTTGAAGGGTGGTATCAGCCAACAGCCTGACATTTCCTTCAAG

>D1-R

TTTTTCAGAGAACCAGAGATTACGCGCAGACCAAAACGATCTCAAGAAGATCCTTTGATTTTCTACCGAAGAAAGGCCCACCCGTGAAGGTGAGCCAGTGAGTTGATTGTGTAAAACGACGGCCAGTGAATTCCTCGCTGCAGTCCTGAAGCTTCCAGGTCAGAAGCGGTTTTCGGGAGTAGTGCCCCAACTGGGGTAACCTTTGGGCTCCCCGGGCGCGTACTCCACCTCACCCATCTGGTCCATCATGATGAACGGGTCGGCTAGCCGAAATTAATACGACTCACTATAGGGAGACCACAACGGTTTCCCTCTAGACACTCGAGTAACTAGTTAACCCCTTGGGGCCTCTAAACGGGTCTTGAGGGGTTTTTTGCTGAAAGGAGGAACTGAGGCGAGTGTTACTTCAACCTGGTCTACTGACTCGCTAACATTAATAAATAAGGAGGCTCTAATGGCACTCATTAGCCAATCAATCAAGACGTAAGGGTG

>D1

TTTTTCAGAGAACCAGAGATTACGCGCAGACCAAAACGATCTCAAGAAGATCCTTTGATTTTCTACCGAAGAAAGGCCCACCCGTGAAGGTGAGCCAGTGAGTTGATTGTGTAAAACGACGGCCAGTGAATTCCTCGCTGCAGTCCTGAAGCTT**CCAGGTCAGAAGCGGTTTTCGGGAGTAGTGCCCCAACTGGGGTAACCTTTGGGCTCCCCGGGCGCGTACTCCACCTCACCCATC**TGGTCCATCATGATGAACGGGTCGGCTAGCCGAAATTAATACGACTCACTATAGGGAGACCACAACGGTTTCCCTCTAGACACTCGAGTAACTAGTTAACCCCTTGGGGCCTCTAAACGGGTCTTGAGGGGTTTTTTGCTGAAAGGAGGAACTGAGGCGAGTGTTACTTCAACCTGGTCTACTGACTCGCTAACATTAATAAATAAGGAGGCTCTAATGGCACTCATTAGCCAATCAATCAAGAACTTGAAGGGTGGTATCAGCCAACAGCCTGACATTTCCTTCAAG

>D2-F

ACATGTATGAGATCCTTTGATTTTCTACCGAAGAAAGGCCCACCCGTGAAGGTGAGCCAGTGAGTTGATTGTGTAAAACGACGGCCAGTGAATTCCTCGCTGCAGTCCTGAAGCTTCCAGGTCAGAAGCGGTTTTCGGGAGTAGTGCCCCAACTGGGGTAACCTTTGGGCTCCCCGGGCGCGTACTCCACCTCACCCATCTGGTCCATCATGATGAACGGGTCGGCTAGCCGAAATTAATACGACTCACTATAGGGAGACCACAACGGTTTCCCTCTAGACACTCGAGTAACTAGTTAACCCCTTGGGGCCTCTAAACGGGTCTTGAGGGGTTTTTTGCTGAAAGGAGGAACTGAGGCGAGTGTTACTTCAACCTGGTCTACTGACTCGCTAACATTAATAAATAAGGAGGCTCTAATGGCACTCATTAGCCAATCAATCAAGAACTTGAAGGGTGGTATCAGCCAACAGCCTACATTTTCCTTCAAGT

>D2-R

TTTTTCAGAAAACCAGAGATTACGCGCAGACCAAAACGATCTCAAGAAGATCCTTTGATTTTCTACCGAAGAAAGGCCCACCCGTGAAGGTGAGCCAGTGAGTTGATTGTGTAAAACGACGGCCAGTGAATTCCTCGCTGCAGTCCTGAAGCTTCCAGGTCAGAAGCGGTTTTCGGGAGTAGTGCCCCAACTGGGGTAACCTTTGGGCTCCCCGGGCGCGTACTCCACCTCACCCATCTGGTCCATCATGATGAACGGGTCGGCTAGCCGAAATTAATACGACTCACTATAGGGAGACCACAACGGTTTCCCTCTAGACACTCGAGTAACTAGTTAACCCCTTGGGGCCTCTAAACGGGTCTTGAGGGGTTTTTTGCTGAAAGGAGGAACTGAGGCGAGTGTTACTTCAACCTGGTCTACTGACTCGCTAACATTAATAAATAAGGAGGCTCTAATGGCACTCATTAGCCAATCAATCAAGAGCGAAGGGGT

>D2

TTTTTCAGAAAACCAGAGATTACGCGCAGACCAAAACGATCTCAAGAAGATCCTTTGATTTTCTACCGAAGAAAGGCCCACCCGTGAAGGTGAGCCAGTGAGTTGATTGTGTAAAACGACGGCCAGTGAATTCCTCGCTGCAGTCCTGAAGCTT**CCAGGTCAGAAGCGGTTTTCGGGAGTAGTGCCCCAACTGGGGTAACCTTTGGGCTCCCCGGGCGCGTACTCCACCTCACCCATC**TGGTCCATCATGATGAACGGGTCGGCTAGCCGAAATTAATACGACTCACTATAGGGAGACCACAACGGTTTCCCTCTAGACACTCGAGTAACTAGTTAACCCCTTGGGGCCTCTAAACGGGTCTTGAGGGGTTTTTTGCTGAAAGGAGGAACTGAGGCGAGTGTTACTTCAACCTGGTCTACTGACTCGCTAACATTAATAAATAAGGAGGCTCTAATGGCACTCATTAGCCAATCAATCAAGAACTTGAAGGGTGGTATCAGCCAACAGCCTACATTTTCCTTCAAGT
