# Supplementary material for: A novel approach for T7 bacteriophage genome integration of exogenous DNA
Source: J Biol Eng. 2020 Jan 16;14:2. doi: 10.1186/s13036-019-0224-x (PMC6966851; doi:10.1186/s13036-019-0224-x)
Supplement: Supplementary file 3 — Additional file 3. Sequencing results of engineered T7 phage and the positive recombinants harboring the exogenous DNA were documented in three files respectively, these files comprised the crude statistics as well as the processed sequences in docx format. [file 13036_2019_224_MOESM3_ESM.zip › Additional file 3/T7DG10G11-attB sequencing/T7DG10G11-attB sequencing.docx]

>Expected Sequence

AGTAATCGATTCGAACTTCTAACTAGATCTGTGCTCAAAGAGGAATCTATCAAGGGCGACACGCGAATTCGATATCGCGGCCGCCGATATCAAGCTTATGTAGGTGACGGT**CTCGAAGCCGCGGTGCGGGTGCCAGGGCGTGCCCTTGGGCTCCCCGGGCGCGTACTCCACCTCACCCATC**TGGTCCATCATGATGAACGGGTCGGCTAGCCGAAA**TTAATACGACTCACTATAGG**GAGACCACAACGGTTTCCCTCTAGACACTCGAGTAACTAGTTAACCCCTTGGGGCCTCTAAACGGGTCTTGAGGGGTTTTTTGCTGAAAGGAGGAACTGAGGCGAGTGTTACTTCAACCTGGTCTACTGACTCGCTAACATTAATAAATAAGGAGGCTCTAATGGCACTCATTAGCCAATCAATCAAGAACTTGAAGGGTGGTATCAGCCAACAGCCTGACATCCTTC

>C1 F

GTTCGTAAGATGATTGGCTAATGAGTGCCATTAGAGCCTCCTTATTTATTAATGTTAGCGAGTCAGTAGACCAGGTTGAAGTAACACTCGCCTCAGTTCCTCCTTTCAGCAAAAAACCCCTCAAGACCCGTTTAGAGGCCCCAAGGGGTTAACTAGTTACTCGAGTGTCTAGAGGGAAACCGTTGTGGTCTCCCTATAGTGAGTCGTATTAATTTCGGCTAGCCGACCCGTTCATCATGATGGACCAGATGGGTGAGGTGGAGTACGCGCCCGGGGAGCCCAAGGGCACGCCCTGGCACCCGCACCGCGGCTTCGAGACCGTCACCTACATAAGCTTGATATCGCGGCCGCGATATCGAATTCGCGTGTCGCCCTTGATAGATTCCTCTTTGAGCACAGATCTAGTTAGAAGTTCGAATCGATTACTTTCTGTTCGACTTGACGACGATAGTTGGCATCTGG

CCAGATGCCAACTATCGTCGTCAAGTCGAACAGAAAGTAATCGATTCGAACTTCTAACTAGATCTGTGCTCAAAGAGGAATCTATCAAGGGCGACACGCGAATTCGATATCGCGGCCGCGATATCAAGCTTATGTAGGTGACGGT**CTCGAAGCCGCGGTGCGGGTGCCAGGGCGTGCCCTTGGGCTCCCCGGGCGCGTACTCCACCTCACCCATC**TGGTCCATCATGATGAACGGGTCGGCTAGCCGAAAT**TAATACGACTCACTATAGG**GAGACCACAACGGTTTCCCTCTAGACACTCGAGTAACTAGTTAACCCCTTGGGGCCTCTAAACGGGTCTTGAGGGGTTTTTTGCTGAAAGGAGGAACTGAGGCGAGTGTTACTTCAACCTGGTCTACTGACTCGCTAACATTAATAAATAAGGAGGCTCTAATGGCACTCATTAGCCAATCATCTTACGAAC

>C1 R

CGTAACTTCTAACTAGATCTGTGCTCAAAGAGGAATCTATCAAGGGCGACACGCGAATTCGATATCGCGGCCGCGATATCAAGCTTATGTAGGTGACGGT**CTCGAAGCCGCGGTGCGGGTGCCAGGGCGTGCCCTTGGGCTCCCCGGGCGCGTACTCCACCTCACCCATC**TGGTCCATCATGATGAACGGGTCGGCTAGCCGAAAT**TAATACGACTCACTATAGG**GAGACCACAACGGTTTCCCTCTAGACACTCGAGTAACTAGTTAACCCCTTGGGGCCTCTAAACGGGTCTTGAGGGGTTTTTTGCTGAAAGGAGGAACTGAGGCGAGTGTTACTTCAACCTGGTCTACTGACTCGCTAACATTAATAAATAAGGAGGCTCTAATGGCACTCATTAGCCAATCAATCAAGAACTTGAAGGGTGGTATCAGCCAACAGCCC

>C1

CCAGATGCCAACTATCGTCGTCAAGTCGAACAGAAAGTAATCGATTCGAACTTCTAACTAGATCTGTGCTCAAAGAGGAATCTATCAAGGGCGACACGCGAATTCGATATCGCGGCCGCGATATCAAGCTTATGTAGGTGACGGT**CTCGAAGCCGCGGTGCGGGTGCCAGGGCGTGCCCTTGGGCTCCCCGGGCGCGTACTCCACCTCACCCATC**TGGTCCATCATGATGAACGGGTCGGCTAGCCGAAAT**TAATACGACTCACTATAGG**GAGACCACAACGGTTTCCCTCTAGACACTCGAGTAACTAGTTAACCCCTTGGGGCCTCTAAACGGGTCTTGAGGGGTTTTTTGCTGAAAGGAGGAACTGAGGCGAGTGTTACTTCAACCTGGTCTACTGACTCGCTAACATTAATAAATAAGGAGGCTCTAATGGCACTCATTAGCCAATCAATCAAGAACTTGAAGGGTGGTATCAGCCAACAGCCC

>C2 F

GGATTGATTGGCTAATGAGTGCCATTAGAGCCTCCTTATTTATTAATGTTAGCGAGTCAGTAGACCAGGTTGAAGTAACACTCGCCTCAGTTCCTCCTTTCAGCAAAAAACCCCTCAAGACCCGTTTAGAGGCCCCAAGGGGTTAACTAGTTACTCGAGTGTCTAGAGGGAAACCGTTGTGGTCTCCCTATAGTGAGTCGTATTAATTTCGGCTAGCCGACCCGTTCATCATGATGGACCAGATGGGTGAGGTGGAGTACGCGCCCGGGGAGCCCAAGGGCACGCCCTGGCACCCGCACCGCGGCTTCGAGACCGTCACCTACATAAGCTTGATATCGCGGCCGCGATATCGAATTCGCGTGTCGCCCTTGATAGATTCCTCTTTGAGCACAGATCTAGTTAGAAGTTCGAATCGATTACTTTCTGTTCGACTTGACGACGATAGTTGGCATCTTCGAATACCGAAA

TTTCGGTATTCGAAGATGCCAACTATCGTCGTCAAGTCGAACAGAAAGTAATCGATTCGAACTTCTAACTAGATCTGTGCTCAAAGAGGAATCTATCAAGGGCGACACGCGAATTCGATATCGCGGCCGCGATATCAAGCTTATGTAGGTGACGGT**CTCGAAGCCGCGGTGCGGGTGCCAGGGCGTGCCCTTGGGCTCCCCGGGCGCGTACTCCACCTCACCCATC**TGGTCCATCATGATGAACGGGTCGGCTAGCCGAAAT**TAATACGACTCACTATAGG**GAGACCACAACGGTTTCCCTCTAGACACTCGAGTAACTAGTTAACCCCTTGGGGCCTCTAAACGGGTCTTGAGGGGTTTTTTGCTGAAAGGAGGAACTGAGGCGAGTGTTACTTCAACCTGGTCTACTGACTCGCTAACATTAATAAATAAGGAGGCTCTAATGGCACTCATTAGCCAATCAATCC

>C2 R

CTCATTCGAACTTCTAACTAGATCTGTGCTCAAAGAGGAATCTATCAAGGGCGACACGCGAATTCGATATCGCGGCCGCGATATCAAGCTTATGTAGGTGACGGT**CTCGAAGCCGCGGTGCGGGTGCCAGGGCGTGCCCTTGGGCTCCCCGGGCGCGTACTCCACCTCACCCATC**TGGTCCATCATGATGAACGGGTCGGCTAGCCGAAAT**TAATACGACTCACTATAGG**GAGACCACAACGGTTTCCCTCTAGACACTCGAGTAACTAGTTAACCCCTTGGGGCCTCTAAACGGGTCTTGAGGGGTTTTTTGCTGAAAGGAGGAACTGAGGCGAGTGTTACTTCAACCTGGTCTACTGACTCGCTAACATTAATAAATAAGGAGGCTCTAATGGCACTCATTAGCCAATCAATCAAGAACTTGAAGGGTGGTATCAGCCAACAGCCTGCCATTTCCTTCAAG

>C2

TTTCGGTATTCGAAGATGCCAACTATCGTCGTCAAGTCGAACAGAAAGTAATCGATTCGAACTTCTAACTAGATCTGTGCTCAAAGAGGAATCTATCAAGGGCGACACGCGAATTCGATATCGCGGCCGCGATATCAAGCTTATGTAGGTGACGGT**CTCGAAGCCGCGGTGCGGGTGCCAGGGCGTGCCCTTGGGCTCCCCGGGCGCGTACTCCACCTCACCCATC**TGGTCCATCATGATGAACGGGTCGGCTAGCCGAAAT**TAATACGACTCACTATAGG**GAGACCACAACGGTTTCCCTCTAGACACTCGAGTAACTAGTTAACCCCTTGGGGCCTCTAAACGGGTCTTGAGGGGTTTTTTGCTGAAAGGAGGAACTGAGGCGAGTGTTACTTCAACCTGGTCTACTGACTCGCTAACATTAATAAATAAGGAGGCTCTAATGGCACTCATTAGCCAATCAATCAAGAACTTGAAGGGTGGTATCAGCCAACAGCCTGCCATTTCCTTCAAG

>C3 F

ATGTCGATCTGATTGATTGGCTAATGAGTGCCATTAGAGCCTCCTTATTTATTAATGTTAGCGAGTCAGTAGACCAGGTTGAAGTAACACTCGCCTCAGTTCCTCCTTTCAGCAAAAAACCCCTCAAGACCCGTTTAGAGGCCCCAAGGGGTTAACTAGTTACTCGAGTGTCTAGAGGGAAACCGTTGTGGTCTCCCTATAGTGAGTCGTATTAATTTCGGCTAGCCGACCCGTTCATCATGATGGACCAGATGGGTGAGGTGGAGTACGCGCCCGGGGAGCCCAAGGGCACGCCCTGGCACCCGCACCGCGGCTTCGAGACCGTCACCTACATAAGCTTGATATCGCGGCCGCGATATCGAATTCGCGTGTCGCCCTTGATAGATTCCTCTTTGAGCACAGATCTAGTTAGAAGTTCGAATCGATTACTTTCTGTTCGACTTGACGACGATAGTTGGCATCTTCGGA

TCCGAAGATGCCAACTATCGTCGTCAAGTCGAACAGAAAGTAATCGATTCGAACTTCTAACTAGATCTGTGCTCAAAGAGGAATCTATCAAGGGCGACACGCGAATTCGATATCGCGGCCGCGATATCAAGCTTATGTAGGTGACGGT**CTCGAAGCCGCGGTGCGGGTGCCAGGGCGTGCCCTTGGGCTCCCCGGGCGCGTACTCCACCTCACCCATC**TGGTCCATCATGATGAACGGGTCGGCTAGCCGAAAT**TAATACGACTCACTATAGG**GAGACCACAACGGTTTCCCTCTAGACACTCGAGTAACTAGTTAACCCCTTGGGGCCTCTAAACGGGTCTTGAGGGGTTTTTTGCTGAAAGGAGGAACTGAGGCGAGTGTTACTTCAACCTGGTCTACTGACTCGCTAACATTAATAAATAAGGAGGCTCTAATGGCACTCATTAGCCAATCAATCAGATCGACAT

>C3 R

CGATTCGAACTTCTAACTAGATCTGTGCTCAAAGAGGAATCTATCAAGGGCGACACGCGAATTCGATATCGCGGCCGCGATATCAAGCTTATGTAGGTGACGGT**CTCGAAGCCGCGGTGCGGGTGCCAGGGCGTGCCCTTGGGCTCCCCGGGCGCGTACTCCACCTCACCCATC**TGGTCCATCATGATGAACGGGTCGGCTAGCCGAAAT**TAATACGACTCACTATAGG**GAGACCACAACGGTTTCCCTCTAGACACTCGAGTAACTAGTTAACCCCTTGGGGCCTCTAAACGGGTCTTGAGGGGTTTTTTGCTGAAAGGAGGAACTGAGGCGAGTGTTACTTCAACCTGGTCTACTGACTCGCTAACATTAATAAATAAGGAGGCTCTAATGGCACTCATTAGCCAATCAATCAAGAACTTGAAGGGTGGTATCAGCCAACAGCC

>C3

TCCGAAGATGCCAACTATCGTCGTCAAGTCGAACAGAAAGTAATCGATTCGAACTTCTAACTAGATCTGTGCTCAAAGAGGAATCTATCAAGGGCGACACGCGAATTCGATATCGCGGCCGCGATATCAAGCTTATGTAGGTGACGGT**CTCGAAGCCGCGGTGCGGGTGCCAGGGCGTGCCCTTGGGCTCCCCGGGCGCGTACTCCACCTCACCCATC**TGGTCCATCATGATGAACGGGTCGGCTAGCCGAAAT**TAATACGACTCACTATAGG**GAGACCACAACGGTTTCCCTCTAGACACTCGAGTAACTAGTTAACCCCTTGGGGCCTCTAAACGGGTCTTGAGGGGTTTTTTGCTGAAAGGAGGAACTGAGGCGAGTGTTACTTCAACCTGGTCTACTGACTCGCTAACATTAATAAATAAGGAGGCTCTAATGGCACTCATTAGCCAATCAATCAAGAACTTGAAGGGTGGTATCAGCCAACAGCC

>C4 F

AATCGAGTGCCATTAGAGCCTCCTTATTTATTAATGTTAGCGAGTCAGTAGACCAGGTTGAAGTAACACTCGCCTCAGTTCCTCCTTTCAGCAAAAAACCCCTCAAGACCCGTTTAGAGGCCCCAAGGGGTTAACTAGTTACTCGAGTGTCTAGAGGGAAACCGTTGTGGTCTCCCTATAGTGAGTCGTATTAATTTCGGCTAGCCGACCCGTTCATCATGATGGACCAGATGGGTGAGGTGGAGTACGCGCCCGGGGAGCCCAAGGGCACGCCCTGGCACCCGCACCGCGGCTTCGAGACCGTCACCTACATAAGCTTGATATCGCGGCCGCGATATCGAATTCGCGTGTCGCCCTTGATAGATTCCTCTTTGAGCACAGATCTAGTTAGAAGTTCGAATCGATTACTTTCTGTTCGACTTGACGACGATAGTTGGCATCTGTCGCATACCGAA

TTCGGTATGCGACAGATGCCAACTATCGTCGTCAAGTCGAACAGAAAGTAATCGATTCGAACTTCTAACTAGATCTGTGCTCAAAGAGGAATCTATCAAGGGCGACACGCGAATTCGATATCGCGGCCGCGATATCAAGCTTATGTAGGTGACGGT**CTCGAAGCCGCGGTGCGGGTGCCAGGGCGTGCCCTTGGGCTCCCCGGGCGCGTACTCCACCTCACCCATC**TGGTCCATCATGATGAACGGGTCGGCTAGCCGAAAT**TAATACGACTCACTATAGG**GAGACCACAACGGTTTCCCTCTAGACACTCGAGTAACTAGTTAACCCCTTGGGGCCTCTAAACGGGTCTTGAGGGGTTTTTTGCTGAAAGGAGGAACTGAGGCGAGTGTTACTTCAACCTGGTCTACTGACTCGCTAACATTAATAAATAAGGAGGCTCTAATGGCACTCGATT

>C4 R

CGCAACTTTCTAACTAGATCTGTGCTCAAAGAGGAATCTATCAAGGGCGACACGCGAATTCGATATCGCGGCCGCGATATCAAGCTTATGTAGGTGACGGT**CTCGAAGCCGCGGTGCGGGTGCCAGGGCGTGCCCTTGGGCTCCCCGGGCGCGTACTCCACCTCACCCATC**TGGTCCATCATGATGAACGGGTCGGCTAGCCGAAAT**TAATACGACTCACTATAGG**GAGACCACAACGGTTTCCCTCTAGACACTCGAGTAACTAGTTAACCCCTTGGGGCCTCTAAACGGGTCTTGAGGGGTTTTTTGCTGAAAGGAGGAACTGAGGCGAGTGTTACTTCAACCTGGTCTACTGACTCGCTAACATTAATAAATAAGGAGGCTCTAATGGCACTCATTAGCCAATCAATCAAGAACTTGAAGGGTGGTATCAGCCAACAGCCTGCCAATCCTTCCAG

>C4

TTCGGTATGCGACAGATGCCAACTATCGTCGTCAAGTCGAACAGAAAGTAATCGATTCGAACTTCTAACTAGATCTGTGCTCAAAGAGGAATCTATCAAGGGCGACACGCGAATTCGATATCGCGGCCGCGATATCAAGCTTATGTAGGTGACGGT**CTCGAAGCCGCGGTGCGGGTGCCAGGGCGTGCCCTTGGGCTCCCCGGGCGCGTACTCCACCTCACCCATC**TGGTCCATCATGATGAACGGGTCGGCTAGCCGAAAT**TAATACGACTCACTATAGG**GAGACCACAACGGTTTCCCTCTAGACACTCGAGTAACTAGTTAACCCCTTGGGGCCTCTAAACGGGTCTTGAGGGGTTTTTTGCTGAAAGGAGGAACTGAGGCGAGTGTTACTTCAACCTGGTCTACTGACTCGCTAACATTAATAAATAAGGAGGCTCTAATGGCACTCATTAGCCAATCAATCAAGAACTTGAAGGGTGGTATCAGCCAACAGCCTGCCAATCCTTCCAG

>C5 F

AATCGAGTGCCATTAGAGCCTCCTTATTTATTAATGTTAGCGAGTCAGTAGACCAGGTTGAAGTAACACTCGCCTCAGTTCCTCCTTTCAGCAAAAAACCCCTCAAGACCCGTTTAGAGGCCCCAAGGGGTTAACTAGTTACTCGAGTGTCTAGAGGGAAACCGTTGTGGTCTCCCTATAGTGAGTCGTATTAATTTCGGCTAGCCGACCCGTTCATCATGATGGACCAGATGGGTGAGGTGGAGTACGCGCCCGGGGAGCCCAAGGGCACGCCCTGGCACCCGCACCGCGGCTTCGAGACCGTCACCTACATAAGCTTGATATCGCGGCCGCGATATCGAATTCGCGTGTCGCCCTTGATAGATTCCTCTTTGAGCACAGATCTAGTTAGAAGTTCGAATCGATTACTTTCTGTTCGACTTGACGACGATAGTTGGCATCTGGCTAATACCGAAAC

GTTTCGGTATTAGCCAGATGCCAACTATCGTCGTCAAGTCGAACAGAAAGTAATCGATTCGAACTTCTAACTAGATCTGTGCTCAAAGAGGAATCTATCAAGGGCGACACGCGAATTCGATATCGCGGCCGCGATATCAAGCTTATGTAGGTGACGGT**CTCGAAGCCGCGGTGCGGGTGCCAGGGCGTGCCCTTGGGCTCCCCGGGCGCGTACTCCACCTCACCCATC**TGGTCCATCATGATGAACGGGTCGGCTAGCCGAAAT**TAATACGACTCACTATAGG**GAGACCACAACGGTTTCCCTCTAGACACTCGAGTAACTAGTTAACCCCTTGGGGCCTCTAAACGGGTCTTGAGGGGTTTTTTGCTGAAAGGAGGAACTGAGGCGAGTGTTACTTCAACCTGGTCTACTGACTCGCTAACATTAATAAATAAGGAGGCTCTAATGGCACTCGATT

>C5 R

CTCAAGTATCGATTCGAACTTCTAACTAGATCTGTGCTCAAAGAGGAATCTATCAAGGGCGACACGCGAATTCGATATCGCGGCCGCGATATCAAGCTTATGTAGGTGACGGT**CTCGAAGCCGCGGTGCGGGTGCCAGGGCGTGCCCTTGGGCTCCCCGGGCGCGTACTCCACCTCACCCATC**TGGTCCATCATGATGAACGGGTCGGCTAGCCGAAAT**TAATACGACTCACTATAGG**GAGACCACAACGGTTTCCCTCTAGACACTCGAGTAACTAGTTAACCCCTTGGGGCCTCTAAACGGGTCTTGAGGGGTTTTTTGCTGAAAGGAGGAACTGAGGCGAGTGTTACTTCAACCTGGTCTACTGACTCGCTAACATTAATAAATAAGGAGGCTCTAATGGCACTCATTAGCCAATCAATCAAGAACTTGAAGGGTGGTATCAGCCAACAGCCTGCCATTCCTTCAAG

>C5

GTTTCGGTATTAGCCAGATGCCAACTATCGTCGTCAAGTCGAACAGAAAGTAATCGATTCGAACTTCTAACTAGATCTGTGCTCAAAGAGGAATCTATCAAGGGCGACACGCGAATTCGATATCGCGGCCGCGATATCAAGCTTATGTAGGTGACGGT**CTCGAAGCCGCGGTGCGGGTGCCAGGGCGTGCCCTTGGGCTCCCCGGGCGCGTACTCCACCTCACCCATC**TGGTCCATCATGATGAACGGGTCGGCTAGCCGAAAT**TAATACGACTCACTATAGG**GAGACCACAACGGTTTCCCTCTAGACACTCGAGTAACTAGTTAACCCCTTGGGGCCTCTAAACGGGTCTTGAGGGGTTTTTTGCTGAAAGGAGGAACTGAGGCGAGTGTTACTTCAACCTGGTCTACTGACTCGCTAACATTAATAAATAAGGAGGCTCTAATGGCACTCATTAGCCAATCAATCAAGAACTTGAAGGGTGGTATCAGCCAACAGCCTGCCATTCCTTCAAG
