# Supplementary material for: A novel approach for T7 bacteriophage genome integration of exogenous DNA
Source: J Biol Eng. 2020 Jan 16;14:2. doi: 10.1186/s13036-019-0224-x (PMC6966851; doi:10.1186/s13036-019-0224-x)
Supplement: Supplementary file 2 — Additional file 2. Extra information about the development of T7 in vivo integration system as well as the plasmids construction schemes were described in “Development of T7 in vivo integration system.docx” and “Plasmids construction scheme.docx”, respectively. The relevant experiment results were documented in file “Figures of supplementary materials”. [file Fig. S3.pdf › Additional file 2/Figures of supplementary materials/Fig. S3.pdf]

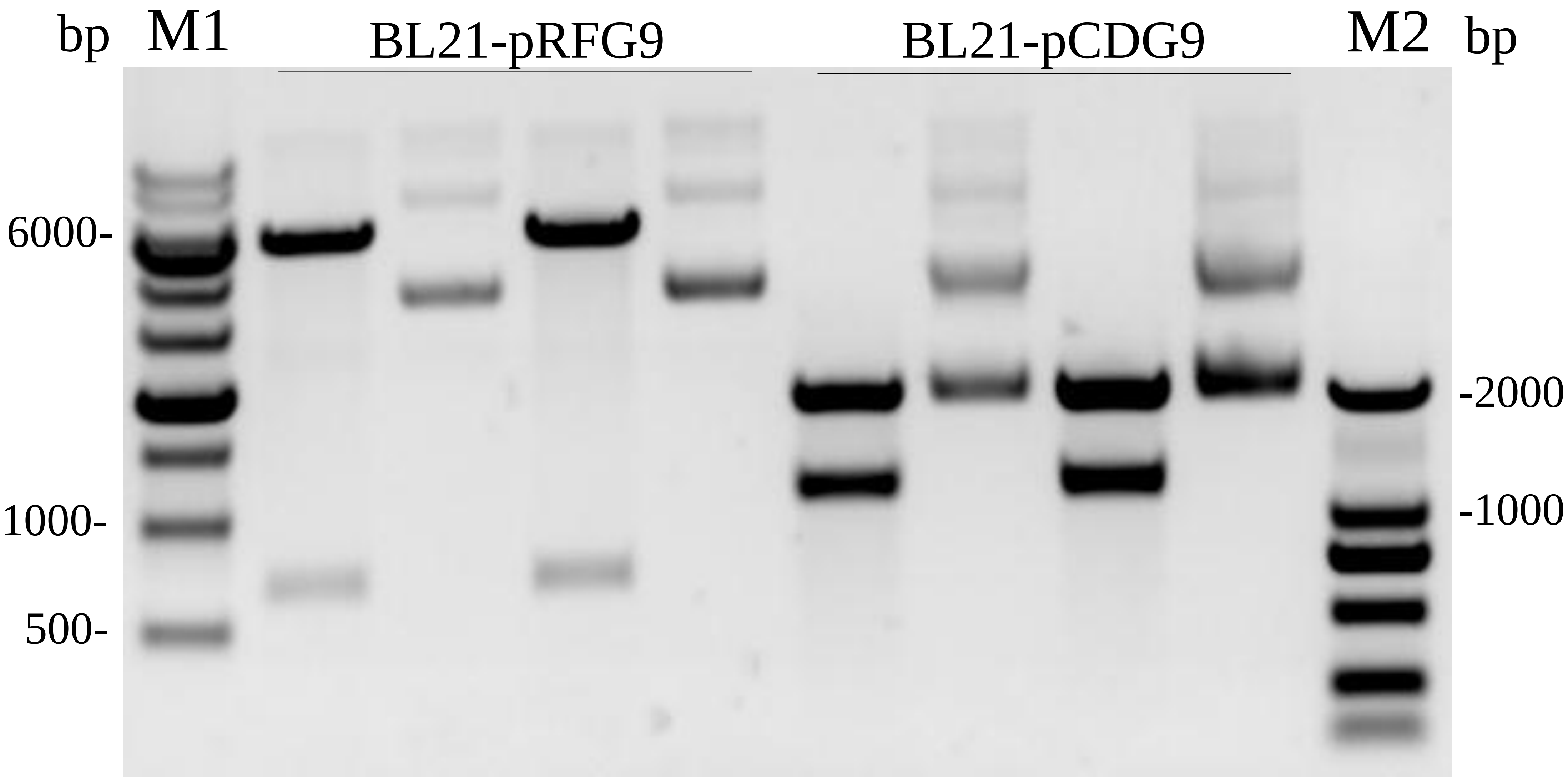
bp
M1
BL21-pRFG9
BL21-pCDG9
M2
bp
6000-
1000-
500-
-2000
-1000
